# Whole genome sequencing of human *Borrelia burgdorferi* isolates reveals linked blocks of accessory genome elements located on plasmids and associated with human dissemination

**DOI:** 10.1371/journal.ppat.1011243

**Published:** 2023-08-31

**Authors:** Jacob E. Lemieux, Weihua Huang, Nathan Hill, Tjasa Cerar, Lisa Freimark, Sergio Hernandez, Matteo Luban, Vera Maraspin, Petra Bogovič, Katarina Ogrinc, Eva Ruzič-Sabljič, Pascal Lapierre, Erica Lasek-Nesselquist, Navjot Singh, Radha Iyer, Dionysios Liveris, Kurt D. Reed, John M. Leong, John A. Branda, Allen C. Steere, Gary P. Wormser, Franc Strle, Pardis C. Sabeti, Ira Schwartz, Klemen Strle

**Affiliations:** 1 Massachusetts General Hospital, Harvard Medical School, Boston, Massachusetts, United States of America; 2 Broad Institute of MIT and Harvard, Cambridge, Massachusetts, United States of America; 3 New York Medical College, Valhalla, New York, United States of America; 4 East Carolina University, Greenville, North Carolina, United States of America; 5 University of Ljubljana, Ljubljana, Slovenia; 6 Wadsworth Center, New York State Department of Health, Albany, New York, United States of America; 7 University Medical Center Ljubljana, Ljubljana, Slovenia; 8 University of Wisconsin, Madison, Wisconsin, United States of America; 9 Tufts University School of Medicine, Boston, Massachusetts, United States of America; 10 Harvard University, Cambridge, Massachusetts, United States of America; 11 Harvard T.H.Chan School of Public Health, Boston, Massachusetts, United States of America; University of Montana, UNITED STATES

## Abstract

Lyme disease is the most common vector-borne disease in North America and Europe. The clinical manifestations of Lyme disease vary based on the genospecies of the infecting *Borrelia burgdorferi* spirochete, but the microbial genetic elements underlying these associations are not known. Here, we report the whole genome sequence (WGS) and analysis of 299 *B*. *burgdorferi* (*Bb*) isolates derived from patients in the Eastern and Midwestern US and Central Europe. We develop a WGS-based classification of *Bb* isolates, confirm and extend the findings of previous single- and multi-locus typing systems, define the plasmid profiles of human-infectious *Bb* isolates, annotate the core and strain-variable surface lipoproteome, and identify loci associated with disseminated infection. A core genome consisting of ~900 open reading frames and a core set of plasmids consisting of lp17, lp25, lp36, lp28-3, lp28-4, lp54, and cp26 are found in nearly all isolates. Strain-variable (accessory) plasmids and genes correlate strongly with phylogeny. Using genetic association study methods, we identify an accessory genome signature associated with dissemination in humans and define the individual plasmids and genes that make up this signature. Strains within the RST1/WGS A subgroup, particularly a subset marked by the OspC type A genotype, have increased rates of dissemination in humans. OspC type A strains possess a unique set of strongly linked genetic elements including the presence of lp56 and lp28-1 plasmids and a cluster of genes that may contribute to their enhanced virulence compared to other genotypes. These features of OspC type A strains reflect a broader paradigm across *Bb* isolates, in which near-clonal genotypes are defined by strain-specific clusters of linked genetic elements, particularly those encoding surface-exposed lipoproteins. These clusters of genes are maintained by strain-specific patterns of plasmid occupancy and are associated with the probability of invasive infection.

## Author summary

Different genotypes of *B*. *burgdorferi* have been associated with different rates of dissemination, but the genetic basis of these differences is not known. We report the genomes of 299 *B*. *burgdorferi* isolates from patients with Lyme disease. We find that whole genome sequence (WGS) type A isolates are a genetically divergent group of isolates characterized by an enlarged pan-genome, an expanded surface lipoproteome encoded on a unique set of plasmids, including lp28-1 and lp56, and increased rates of dissemination. Using genome-wide association methods applied to the *B*. *burgdorferi* pan-genome, we identify loci associated with dissemination. The near-clonal nature of *B*. *burgdorferi* populations means that relationships of individual loci to dissemination are relatively weak after adjusting for the lineage structure among the isolates, implying that experimental studies and larger cohorts are needed to identify the causal alleles within a lineage mediating these effects. Across the isolates studied, an increasing number of surface-expressed lipoproteins was associated with an increased probability of dissemination in humans. The results underscore how strain-specific genetic variation—particularly among surface lipoproteins located on plasmids—is linked to the phenotype of human dissemination. More broadly, this approach provides a foundation for future studies linking spirochete genotype to the diverse clinical phenotypes of Lyme disease in humans.

## Introduction

Lyme disease is a heterogeneous illness caused by spirochetes of the *Borrelia burgdorferi* sensu lato (*Bbsl*, sensu lato meaning ‘in the broad sense’) complex. *Bbsl* contains over 20 subspecies (also termed genospecies, genomic species), four of which cause the majority of infections in humans: *B*. *burgdorferi* sensu stricto (*Bbss*, sensu stricto meaning in the strict sense*)*, *B*. *afzelii*, *B*. *garinii*, and *B*. *bavariensis* [1]. Nearly all Lyme disease in the US is caused by *Bbss*. In Europe, most infections are caused by *B*. *afzelii*, *B*. *garinii*, or *B*. *bavariensis*. Some authors have proposed reclassifying Lyme disease spirochetes as *Borreliella* [2], while others prefer to retain the use of the *Bbsl* designation [3,4]. We focus here on *Bbss*, and refer to this group of spirochetes throughout the manuscript as *Bb*.

Infection with *Bb* usually presents as an expanding skin lesion, erythema migrans (EM), at the site of the tick-bite. If untreated, spirochetes may disseminate to secondary sites, primarily other skin sites, the nervous system and joints [1,5]. In addition to clinical variation caused by different *Bbsl* species, differences in virulence have also been noted among genotypes within *Bb* [6–8], and such phenotypic differences have been recapitulated in murine models [9–11]. These associations imply that microbial genetic loci influence the clinical manifestations of Lyme disease. Despite such evidence linking microbial genotype to clinical phenotype, the specific genes or loci responsible for the clinical manifestations of Lyme disease have not yet been identified.

*Bb* genome analysis has been limited to date due to technical challenges of sequencing and assembly and difficulties of obtaining isolates from patients with Lyme disease [12]. The *Bb* genome consists of a roughly one megabase of core genome (~900Kb chromosome and the plasmids cp26 and lp54), as well as numerous (>15) additional circular and linear extrachromosomal DNA elements (colloquially termed plasmids) [13,14]. Subsets of plasmids have high levels of homology (as exemplified by seven 32 kilobase circular plasmids (cp32) [15] and four 28-kilobase linear plasmids (lp28) [14] in the B31 reference isolate), which have diversified through duplication, recombination, and other primordial evolutionary events [16]. The sheer number of plasmids and their extreme homology has made sequencing and assembly of complete *Bb* genomes a major challenge, particularly with widely-used short read sequencing methods [17].

The technical challenges of sequencing and assembly are compounded by the difficulty of obtaining isolates from human disease. However, it is possible to culture *Bb* from EM lesions in many cases and successful cultivation of *Bb* from blood of infected patients has also been reported. Culture requires specialized techniques which are rarely used in routine clinical practice. The spirochete has occasionally been cultured from cerebrospinal fluid (CSF) in patients with meningitis, but rarely from synovial fluid in patients with Lyme arthritis, the most common late disease manifestation in the US. Thus, the great majority of available *Bb* isolates are from patients with EM, an early disease manifestation. As a result of these challenges, only a small number of human clinical isolates have been sequenced and analyzed. To our knowledge, no large whole genome sequence (WGS) studies of human isolates have been conducted. Fewer than 50 human isolates analyzed by WGS have been publicly reported, either sporadically or included in cohorts consisting primarily of tick-derived isolates and in the majority of studies limited or no clinical information was reported to allow for genotype to phenotype comparisons [18–23].

Genotyping systems have been developed to subclassify *Bb* strains using single or multiple genomic regions (reviewed in [24]). Two of the most commonly used typing methods are based on restriction-fragment length polymorphisms in the 16S-23S ribosomal RNA spacer region [25,26], termed ribosomal spacer type (RST), and on sequence variation of outer surface protein C (OspC), one of the most variable *Bb* proteins [27,28]. RST typing subdivides *Bb* into 3 types, referred to as RST1, RST2, and RST3 [9], whereas OspC typing subdivides *Bb* into ~30 OspC genotypes of which >24 cause infection in humans [29–31]. RST and OspC are in linkage disequilibrium on the core genome, and each RST genotype is generally associated with particular OspC types (e.g., RST1 mostly corresponds to OspC types A and B and RST2 corresponds primarily to OspC types F, H, K and N) [31]), whereas RST3 is the most variable and correlates with the remaining OspC types. In addition to these genotyping methods, multilocus sequence typing (MLST), which is based on eight chromosomal housekeeping genes, has been used to further sub-stratify the strains [31,32]. According to the *Borrelia* MLST database (https://pubmlst.org/borrelia/), >900 MLST sequence types have been identified.

Application of targeted genotyping methods has previously established a link between *Bb* microbial genotype and several phenotypic properties including dissemination in humans, disease severity, immunogenicity, and the type of clinical presentation [1,6,8,9,11,30,31,33–36]. For example, using RST and OspC genotyping we previously showed that RST1 OspC type A strains have greater propensity to disseminate [7,8], are more immunogenic [6], are associated with more symptomatic early infection [6], and with a greater frequency of post-infectious Lyme arthritis (also referred to as antibiotic-refractory Lyme arthritis) [6,37]. However, these approaches lack the resolution to reconstruct a detailed evolutionary history or to define individual genes or loci underlying phenotypic variability. The limitations of previous studies have been further compounded by the absence of large cohorts of patient-derived isolates accompanied by detailed clinical information.

In this study, we used WGS to characterize in detail the genomes–including the core genome and associated plasmids–of 299 patient-derived *Bb* strains. The isolates were collected primarily from patients with EM, over three decades across Northeastern and Midwestern US and Central Europe. Although most isolates were from skin (the site from which *Bb* is most commonly isolated), we assessed dissemination using established methods [7,34] that incorporate clinical signs of dissemination as well as the presence of *Bb* at extra-cutaneous sites as assessed by a positive blood PCR or a positive blood culture (see Methods). We hypothesized that genetic variation in *Bb* open reading frames (ORFs) and plasmids among strains was associated with differences in dissemination in humans. We carried out phylogenetic and phylogeographic analysis, and identified particular *Bb* genomic groups, plasmids, and individual ORFs associated with disseminated human disease.

## Materials and methods

### Ethics statement

This study involves secondary use of deidentified archival clinical isolates and patient data collected in previous studies and was approved by the Massachusetts General Hospital Institutional Review Board (IRB) under protocol 2019P001864. Analysis of deidentified patient data was carried out under a waiver of consent.

### Selection of *B*. *burgdorferi* isolates (see S1 Table)

In total, 299 *Bb* isolates collected from 299 patients over a 30-year period (1992–2021) were included in this study: 201 from the Northeastern US, 62 from the Midwestern US and 36 from Slovenia (Central Europe). The majority (97%) of isolates were derived from skin (n = 287); 9 were from CSF and 2 were from blood. Isolates were cultured in BSK or MKP medium [38,39]. All patients met the US Centers for Disease Control and Prevention (CDC) criteria for Lyme disease [40]. Only low passage isolates (passage <5) were used for WGS.

### Northeastern united states

The 201 isolates from the Northeastern US were collected at two geographic locations: 113 from New England (primarily from contiguous regions of Massachusetts, Rhode Island, and Connecticut) and 88 from New York State. The New York strains belong to a larger collection of more than 400 clinical isolates, collected between 1992–2005, that had been previously typed at the *rrs-rrlA* IGS and *ospC* loci [7,35]. To account for the full diversity of *Bb* genotypes found in the collection, isolates with the best sequence quality from each OspC major group were selected for this study in accordance with their prevalence in the entire collection. All of the latter isolates were cultured from skin biopsies of infected patients, rather than from blood or CSF (S1 and S2 Tables).

#### Midwestern united states

The 62 isolates from the Midwestern US were derived from skin and CSF specimens submitted to the Marshfield Laboratories (Marshfield, WI) for *Borrelia* culture from 1993 to 2003 (S1 and S2 Tables).

#### Central europe (Slovenia)

The 36 isolates from Slovenia represent all *Bb* isolates that were cultured from patients over a 27-year period (1994–2021), who were evaluated at the Lyme borreliosis outpatient clinic at the University Medical Center Ljubljana (UMCL).

#### Selection of patients

Patients included in this study were diagnosed with early Lyme disease and were classified as having either localized or disseminated infection. Early Lyme disease was defined by the presence of at least one EM skin lesion or symptoms consistent with Lyme neuroborreliosis along with a positive CSF culture. Localized infection was defined by a single culture positive EM skin lesion in the absence of clinical and/or microbiological evidence of dissemination to a secondary site. Disseminated infection was defined by a positive blood or CSF culture or a positive PCR on a blood sample, the presence of multiple EM lesions, and/or signs of neurological involvement. Of the 299 isolates, 291 (97.3%) were classified as Disseminated or Localized by these criteria; clinical records were not available to classify the remaining 8 of the 299 (2.7%), and, therefore, these isolates were excluded from analyses of dissemination. A measure of bloodstream dissemination was available for 212/299 (70.9%) of isolates, with blood PCR testing results available for 106/299 (35.4%) and blood culture available for a disjoint set of 106/299 (35.4%) of all isolates. Multiple EM skin lesions were present in 57/290 (19.7%); among patients with a single EM, 23/88 (26.1%) had a positive blood culture and 28/86 (32.6%) had a positive PCR on a blood sample. Lyme neuroborreliosis was defined by clinical criteria and based on assessment by the treating clinician. In Europe, CSF pleocytosis and intrathecal production of *Borrelia* antibodies were required for diagnostic determination of Lyme neuroborreliosis, following guidelines of the European Federation of the Neurological Societies [41]. Summary statistics of isolates by group is provided in S1 Table. The list of isolates and associated metadata is provided in S2 Table.

#### WGS

*Bb* DNA was isolated from the cultured isolates with either the IsoQuick kit (Orca Research, Bothell, WA), the Gentra PureGene DNA Isolation Kit (Qiagen Inc., Valencia, CA), or the DNEasy kit (Qiagen Inc, Valencia, CA). Short-read next-generation sequencing (NGS) library construction was performed using the Nextera XT Library Prep Kit (Illumina, San Diego, CA). DNA quantification was performed in a 96-well microplate using the SpectraMax Quant dsDNA Assay Kit and the Gemini XPS Fluorometer (Molecular Devices, San Jose, CA), or in a single tube using the Qubit 2.0 fluorometer (Thermo Fisher Scientific, Springfield Township, NJ). Library quality was examined using the 4200 TapeStation and D1000 ScreenTape (Agilent, Santa Clara, CA). Paired-end sequencing (2 × 150 or 250 cycles) was performed using the NextSeq 550 or MiSeq system (Illumina).

#### Bioinformatics data analysis

Trimmomatic v0.39 [42] was used for trimming and cleaning of raw sequence reads; SPAdes v3.14.1 [43] for *de novo* genome assembly; QUAST [44] for quality assessment and assembly visualization; Kraken2 [45] v2.1.1 for digital cleaning of assembled genomic sequence by using taxonomy classification; mlst v2.19.0 (https://github.com/tseemann) for MLST [46] identification from assembled sequences; k-mer weighted inner product (kWIP) [47] v0.2.0 for alignment-free, k-mer-based relatedness analysis; prokka v1.14.6 [48] for genome sequence annotation; Roary [49] for core- and pan-genome analysis; FastTree v2.1.11 [50] and IQtree [51,52] for phylogeny tree generation; the latter tool was used to generate maximum-likelihood (ML) trees with bootstrap support. Bioconductor [53] packages in R [54] v4.1.1 and/or RStudio v2021.09.0+351, such as ggplot2 [55], ggtree [56], ggtreeExtra, and ggstar, were also used for phylogeny tree plotting. Association of homology groups with dissemination was conducted using PySeer 1.3.10 using the lineage model. MLST definitions were downloaded from pubMLST. Multidimensional scaling (MDS) was calculated on the kWIP distances using the command mdscale() in R. Fisher’s exact test was used for pairwise comparison of categorical variables as implemented with the fisher.test() function in R. The MiniKraken2 database was constructed for Kraken2 from complete bacterial, archaeal, and viral genomes in RefSeq as of March 12, 2020.

Bayesian trees were constructed by running BEAST directly on the core genome alignment from Roary using an HKY substitution model. We constructed maximum clade credibility trees using TreeAnnotator [57]. To construct OspC trees, we extracted annotated OspC sequences from the *de novo* assemblies, filtered for full-length sequences, aligned them using MAFFT [58] and constructed a phylogenetic tree using BEAST v.1.10.4 [57]. We obtained at least 10,000,000 samples from the posterior distribution and inspected the posterior traces for convergence.

To characterize the plasmid content of individual isolates, we took two approaches. We first aligned the contigs to the B31 reference and quantified a plasmid as present or absent if greater than 50% of the reference genome plasmid was covered by contigs. Because homology alone does not necessarily indicate that a plasmid is present [14], as a complementary approach, we built a hidden Markov model (HMM) of PFam32 genes using HMMer [59] and searched the resulting profile against the assemblies to identify PFam32 genes. We then aligned the resulting putative PFam32 genes against a set of canonical PFam32 genes, kindly provided by Dr. Sherwood Casjens, that have been used to determine plasmid types in published reports [12]. For each putative PFam32 gene, if a match with >95% amino acid identity was present in the list of annotated PFam32 genes, we marked the isolate as having a copy of the closest-matching PFam32 based on sequence identity. If no PFam32 within these thresholds could be identified, the closest PFam32 family member was considered unknown and not assigned in this analysis.

## Results

### Whole-genome sequencing of human Borrelia burgdorferi sensu stricto isolates

To gain insight into the evolution, population structure, and pathogenesis of *Bb* in human infection, we sequenced 299 isolates of *Bb* from human cases of early Lyme disease. The *de novo* assemblies produced high-quality, genome assemblies with a median total length of 1.34 megabases (Mb) (IQR 1.30–1.37 Mb). Final assemblies had a median GC content of 28.12% (IQR 27.96–28.22), similar to the 28.18% GC content of the B31 reference strain [13]; contained a median of 107 contigs per isolate (IQR 88.0–137.5); and had a median N50 of 213,476 bases (IQR 80,809–221,506 bases). Median coverage of the genome assemblies was 57.6x (interquartile range [IQR] 27.6x – 130.8x). We were unable to finish assembly of plasmids due to repetitive plasmid sequences. Assembly statistics are given in S3 Table.

**Fig 1:**
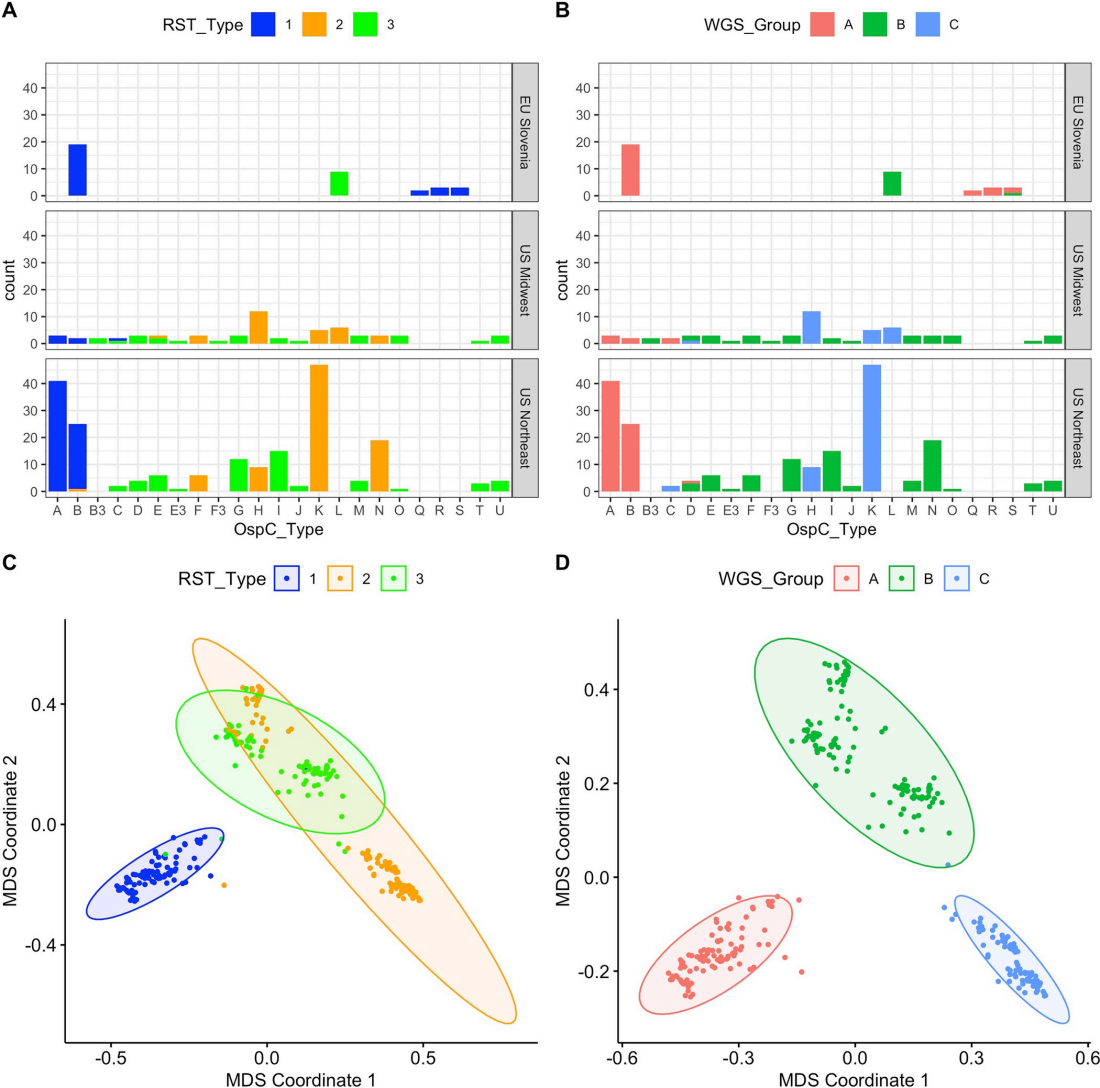
**A.** Counts of samples according to RST and OspC type. Top, middle, and lower panels show samples from different geographic regions. X-axis gives OspC type. Bars are colored according to RST type. **B.** Plots as in (A) but with bars colored according to the WGS group. **C**. Multidimensional scaling (MDS) of 299 *Bb* genomes, with RST type annotated. **D**. MDS of 299 *Bb* genomes, with WGS type annotated.

As an initial characterization of divergence between strains, we applied alignment-free, kmer-based analysis (kWIP) to the WGS data and identified three major clusters based on their genetic distances (Figs 1C, 1D, and S1). This unbiased distance analysis (without any reference or annotation) revealed that a single lineage (WGS A) was divergent from all of the other isolates (Fig 1C and 1D). The remaining isolates are grouped into two stable clusters (WGS groups B and C). RST type 1 was divergent from the other two WGS groups, but RST 2 and 3 were mixed between WGS groups B and C (Fig 1C and 1D).

**Fig 2:**
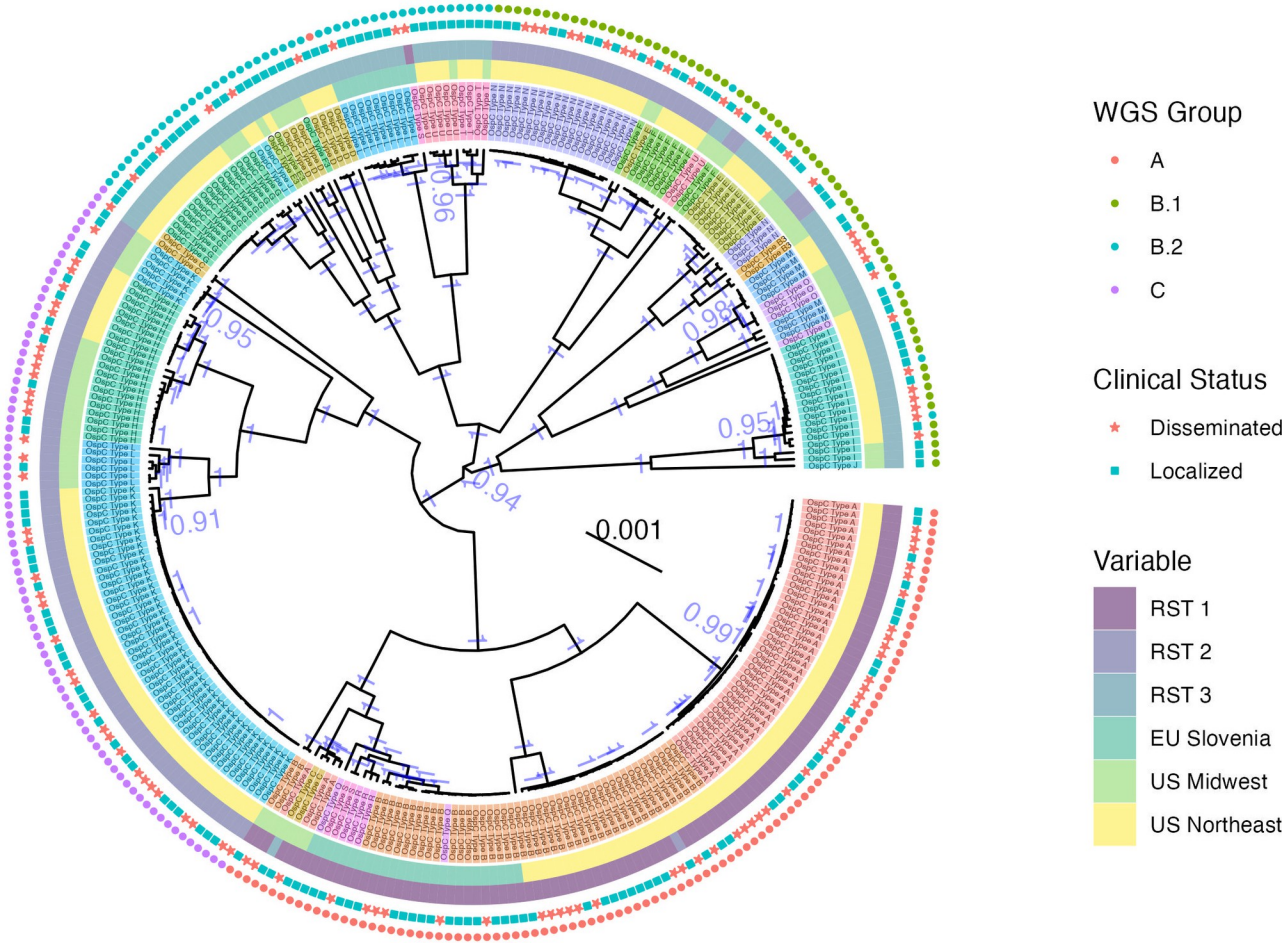
Maximum clade credibility (MCC) core genome phylogenetic tree with metadata annotated adjacent to the tips. OspC types are displayed in color and annotated with text. The region of collection and RST type are labeled by colored boxes adjacent to the tips. Dissemination status is denoted with a star (disseminated isolates) or square (localized). WGS group is labeled by colored points on the outer rim of the. The posterior support for all nodes > 0.9 has been labeled in blue text. The tree scale is in nucleotide substitutions per site.

We next constructed maximum clade credibility (MCC) (Fig 2) and maximum-likelihood (ML) (S2A Fig) phylogenetic trees using core genome elements (as defined by Roary [49], see Methods) from WGS. WGS groups defined by k-mer distance corresponded to the clade structure on the core-genome tree and the associated OspC types (Figs 2 and S2). In addition, they revealed substructure within these groups, particularly WGS group B, which we split into subclusters B.1 and B.2 (Figs 2 and S3B). ML and MCC trees were in broad agreement, and the posterior probability of all nodes separating WGS groups was > 0.99 (similarly, bootstrap support >99% on the ML tree), indicating that the distance-based clustering was phylogenetically well-supported.

We compared WGS-based classification to existing targeted typing methods. WGS groups were strongly associated with RST (Fig 1A and 1B, Fisher’s exact test, p < 1 x 10^−6^) and OspC type (Figs 1A, 1B, and S1; Fisher’s exact test, p < 1 x 10^−6^). RST1 / Osp C type A/B sequences consistently clustered as a single clade in the core genome phylogenetic tree and MDS of k-mer distances (Figs 1C and S1), demonstrating agreement between typing methods. In contrast, RST2 and RST3 were both polyphyletic in the WGS data and contained within separate WGS groups (Fig 1C and 1D). Similarly, OspC types were monophyletic on the WGS tree (Fig 2) and on a tree built from OspC sequences (S2C Fig), but there were some instances of closely related OspC sequences that were part of distinct WGS groups (S2C and S2D Fig). For example, the OspC type L isolates from the Midwestern US and Slovenia are on different branches of the core genome phylogenetic tree (Figs 2, S2C, and S2D). Thus, RST and OspC typing methods identify substructure in *Bb* genomes, and largely agree on the divergent RST1 / OspC A/B clade, but RST does not capture fine-grain genetic structure, and the frequency of recombination at the OspC locus means that there are instances in which the genetic distances between OspC sequences is a poor measure of core genome distance.

### Population geographic structure

We next explored the relationship between genetic markers and geography. WGS group was strongly associated with broad geographic region (US Northeast, US Midwest, EU Slovenia) (Fisher’s exact test, p < 1 x 10^−6^), similar to the findings with previously evaluated genetic markers including RST (Fisher’s exact test, p < 1 x 10^−6^) and OspC type (Fisher’s exact test, p < 1 x 10^−6^) (counts by geographic region are shown in Fig 1A and 1B).

**Fig 3:**
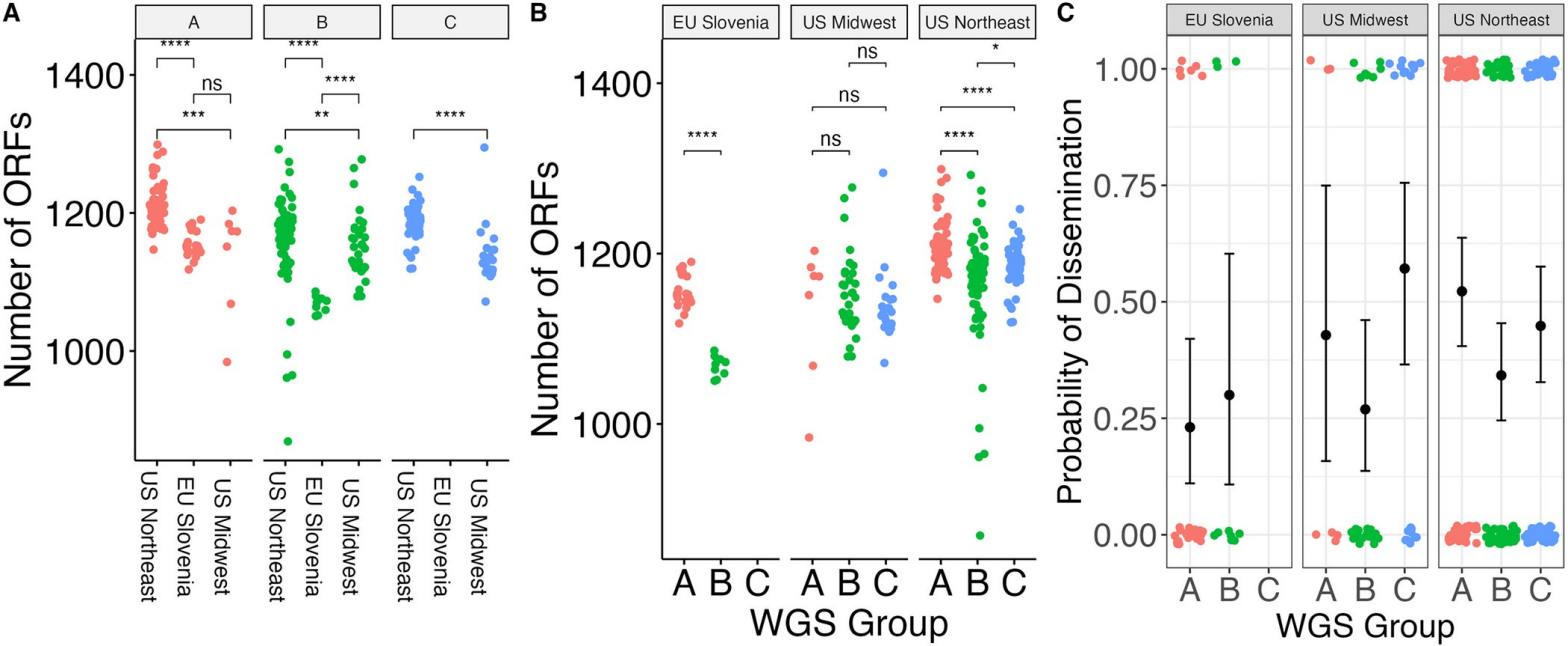
**A.** Number of ORFs by geographic region in different WGS groups. * denotes p < 0.05; ** denotes p < 0.01; *** denotes p < 0.001; **** denotes p < 0.0001; ns—not significant (as assessed by Wilcoxon rank-sum test). **B.** Number of ORFs by WGS group in different geographic regions, with assessment of statistical significance as in (**A**). **C.** Probability of dissemination by genomic group. Each point represents a sample. Points are colored by WGS group. The samples that disseminated have been plotted at y = 1; those that did not have been plotted at y = 0. Random noise has been added to the x- and y- coordinate to display the points. The mean +/- 95% binomial confidence interval is shown for each group with error bars.

The number of ORFs in the genome differed significantly by region within a given WGS group (Fig 3A). In the US Northeast and in Slovenia, WGS groups differed significantly by the number of ORFs (Fig 3B). These differences are not attributable to reference genome bias because the ORF counts were derived from annotated *de novo* assemblies. As core genome size is relatively constant among strains regardless of geographic location, the differences in accessory genome size across different populations, even within a given genomic group with a single common ancestor, suggests that the diversification of accessory genome size may be one mechanism by which strains adapt to distinct ecological factors in each geographic region. Slovenian isolates were clustered in two well-defined monophyletic groups (Figs 2 and S2C), suggesting at least two inter-continental exchanges (Figs 2 and S2C), consistent with a previous report [19].

### Associations between genotype and Bb dissemination in patients

A primary goal of sequencing clinical isolates is to identify bacterial genetic associations with clinical phenotypes. We hypothesized that certain genetic elements are associated with spirochetal dissemination in humans. Dissemination is a prerequisite for the progression of disease from an EM skin lesion to more severe Lyme disease complications such as meningitis, carditis, and arthritis. Given the previously-reported associations between single-locus genetic markers and dissemination [7,8,11,34], we investigated the relationship between genotype and dissemination in humans. We scored isolates as either disseminated or localized based on specific clinical characteristics of the patients from whom they were obtained, particularly presence of multiple vs a solitary EM skin lesion and neurologic signs and symptoms of Lyme disease, as well as having positive culture or PCR results for *Bb* in blood.

WGS groups differed from each other in their propensity to disseminate (p = 0.059 for 3 groups; p = 0.012 for 4 groups, Fisher’s exact test) (Figs 3C and S3C and S4 Table). Slovenian isolates disseminated at a lower rate (25%) than US isolates (42.7%) (p = 0.045, Fisher’s exact test), and the relationship between WGS groups and human dissemination was slightly stronger when testing US isolates only (p = 0.02 for 3 groups; p = 0.004 for 4 groups, Fisher’s exact test). WGS group A isolates from the US, which correlate with OspC type A and RST1 strains, showed the highest rate of human dissemination (51.4%) whereas US WGS group B isolates had the lowest rate of human dissemination (32.4%). Within WGS group B, there was evidence of substructure (S3 Fig). US B.1 isolates disseminated at a higher rate (40.0%) than B.2 isolates (18.4%) (S3C Fig).

Consistent with previous observations [6,7] and with the general alignment of WGS, RST, and OspC type, RST type was also associated with dissemination (p = 0.010, Fisher’s exact test), with RST1 having the greatest propensity to disseminate and RST3 the lowest [7,8] (S4B Fig). OspC type A was also associated with dissemination (p = 0.008, Fisher’s exact test, S4A Fig). A significant association with dissemination could not be detected when OspC type was tested as a categorical variable with 23 categories (p = 0.3, Fisher’s exact test, S4 Fig), likely because of the reduced power due to many categories.

The propensity to disseminate varied greatly among the US and Slovenian isolates, which is likely due to the major genetic differences in isolates between the two regions (Fig 3C). In Slovenia, the predominant WGS group A isolates are OspC type B and all the WGS B.2 isolates are OspC type L (S4 Fig). This correlation was particularly notable for WGS A strains, which were recovered from patients with disseminated Lyme disease at a rate of 51.4% in the US vs 23.1% in Slovenia. WGS-B.2 isolates in the US were associated with the lowest dissemination rate (18.4%), whereas those from Slovenia showed a higher dissemination rate of 30% (Figs 3C and S4A). Taken together, these data confirm that rates of dissemination vary by genotype and demonstrate that WGS A/RST1, particularly a subset distinguished by OspC type A strains, is a genetically distinct lineage with higher rates of dissemination.

### Plasmid associations with WGS profiles

**Fig 4:**
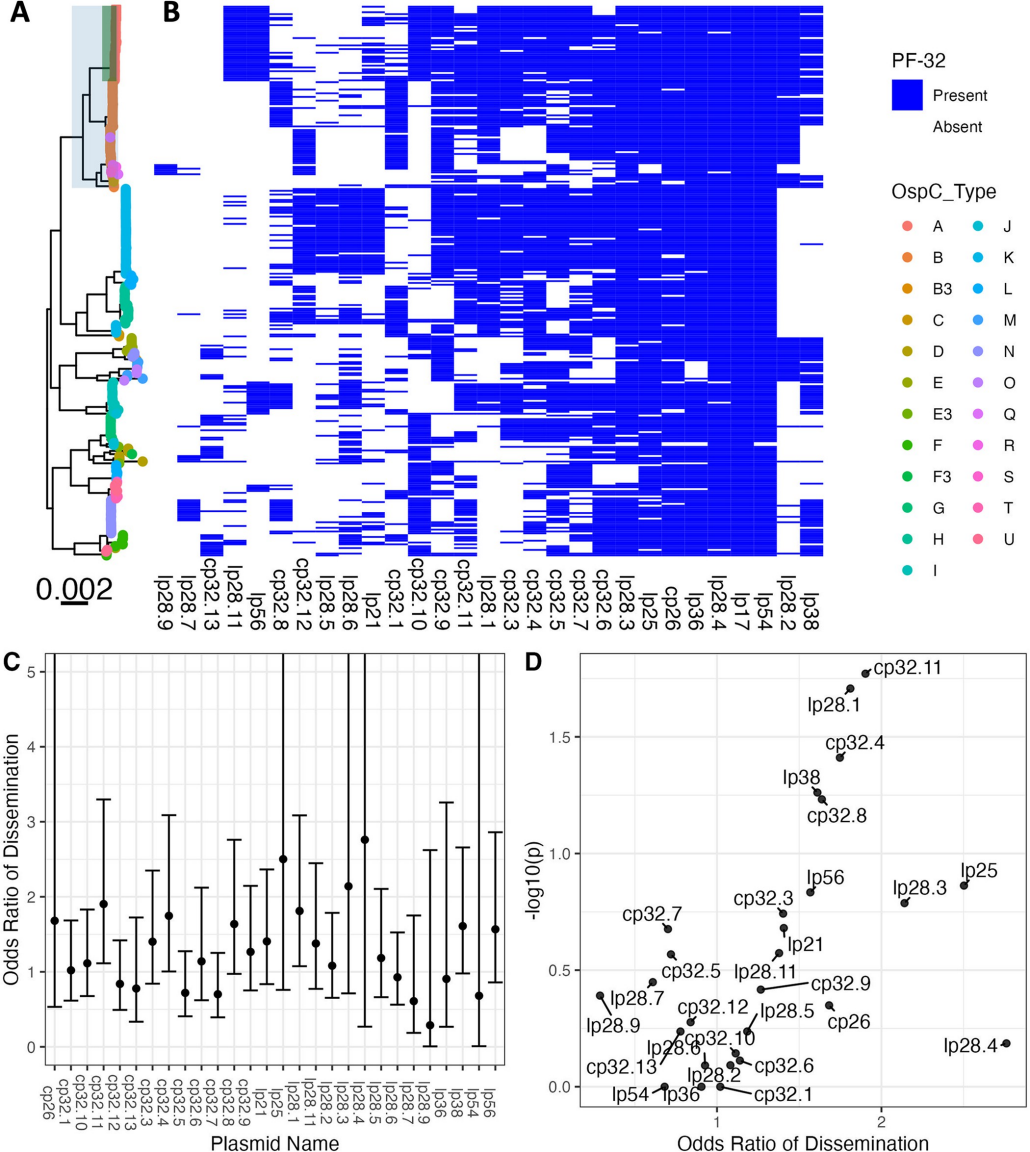
**A.** Core genome maximum likelihood phylogeny with tips colored by OspC type. The clade corresponding to RST1 is shaded in light blue and the clade corresponding to OspC type A is shaded in green. **B.** The matrix at the right shows the presence or absence of individual plasmids using the presence or absence of PFam32 plasmid-compatibility genes as a proxy. The columns of the matrix have been clustered using hierarchical clustering. The rows of the matrix are ordered according to the midpoint rooted maximum likelihood phylogeny shown at left. **C.** Odds ratio of dissemination and confidence interval by plasmid, inferred by PFam32 sequences. **D.** Volcano plot displaying the -log10 P value (as calculated using Fisher’s exact test) and the odds ratio of dissemination for each plasmid, inferred by Pfam32 sequences.

As most of the genetic variation in *Bb* occurs on plasmids [12,60,61], we investigated the variation in plasmid content across genotypes. Assembly and analysis of plasmid sequences is challenging because the length of repeated sequences in plasmids is greater than the read length generated by the short-read Illumina sequencing technology used in this study [17]. To circumvent this, we exploited the relationship between plasmid partition genes (paralogous family 32; PFam32) and plasmid types [12,16], putatively identifying the presence or absence of a plasmid by the presence/absence of unique PFam32 sequences (Fig 4). After annotating all PFam32 genes in the assemblies using an HMM, we linked each putative PFam32 to a plasmid by finding the closest match by sequence homology from a curated list of PFam32 protein sequences (see Methods).

Applying this method to each strain, we created a provisional map of plasmids across *Bb* strains (Fig 4A and 4B). While a few plasmids are found more broadly, distinct genotypes and WGS groups contain unique collections of plasmids. Several plasmids, including cp26, lp54, lp17, lp36, lp25, lp28-4, lp28-3 are found in nearly all isolates (Fig 4A and 4B) and others such as cp32-7, cp32-5, cp32-6, cp32-9, and cp32-3 are found in most strains. Other plasmids were more variable and only found in certain genotypes. OspC type A strains possessed a distinct plasmid profile, containing lp56 and a unique version of lp28-1 (marked by the lp28-1 PFam32 as well as a previously-annotated “orphan” PFam32 sequence, BB_F13. When found in isolation, BB_F13 defines an lp28-11 plasmid [12], so was annotated as such. However, in many cases it may signify a subtype of lp28-1 rather than an entirely new plasmid, especially OspC type A isolates whose sequence is likely similar to the B31 reference strain [13,14]). Based on PFam32 sequences, WGS A strains also contained lp28-2 and most also contained lp38. OspC type K strains also contained a relatively homogenous subset of plasmids including lp21, lp28-5, lp28-6, cp32-12. WGS-A/ RST1 genotypes were the least heterogeneous with respect to plasmid diversity and OspC type, whereas WGS-B and WGS-C groups (RST2 and RST3) were more diverse, although the subset of RST2 strains consisting of OspC type K isolates was also relatively homogenous. Curiously, lp28-9 was found only in Slovenian RST1 isolates (Fig 4), the majority of which were OspC type B (Fig 1); cp32-12, cp32-9, and cp32-1 were also found more commonly in Slovenian isolates.

Many plasmids (e.g. lp28-1, lp28-2, lp38 and numerous others) were found in multiple distinct branches of the phylogenetic tree suggesting a complex inheritance pattern of polyphyletic loss and/or recombination. This is consistent with the observed reassortment between core genome elements and OspC (S2C and S2D Fig). For example, OspC types B and N both contained cp32-8, whereas OspC type K genotype is most closely correlated with the lp21, lp28-5 and cp32-12 pattern. lp56 is associated with OspC type A and OspC type I.

Specific plasmids showed significant associations with dissemination. The presence of lp28-1 was associated with dissemination (OR 1.8, p = 0.02, Fisher’s exact test), as was cp32-11 (OR 1.9, p = 0.02) and cp32-4 (OR 1.7, p = 0.04) (Fig 4C and 4D and S5 Table). In addition, the lp38 plasmid is present in roughly half of US isolates but absent in all Slovenian isolates and demonstrated a trend toward being associated with dissemination (OR 1.6, p = 0.05) which may explain the lower frequency of dissemination generally observed with European *Bb*.

To confirm the accuracy of these plasmid differences across genotype, we also constructed a map of plasmid occupancy across strains by an alternate approach. We aligned contigs from assembled genomes to the B31 reference sequence and annotated a plasmid as “present” if the assembled contigs covered a majority of the reference plasmid sequence (S5A–S5C Fig). Only plasmids present in the B31 reference genome are considered in this analysis. These results were qualitatively similar to those obtained using the PFam32 sequences (S5 Fig and S6 Table) suggesting that cp26, lp54, lp17, lp28-3, lp28-4 and lp36 were present in nearly all strains whereas other plasmids were more variable.

Together, these analyses reveal a core set of plasmids present across *Bb* strains as well as strain-variable plasmids that are associated with distinct geographic and clinical features (i.e., propensity to disseminate) of *Bb*, suggesting that they contain individual bacterial genetic elements that may underlie distinct disease phenotypes.

### Strain variation in core, accessory, and surface lipoproteome

**Fig 5:**
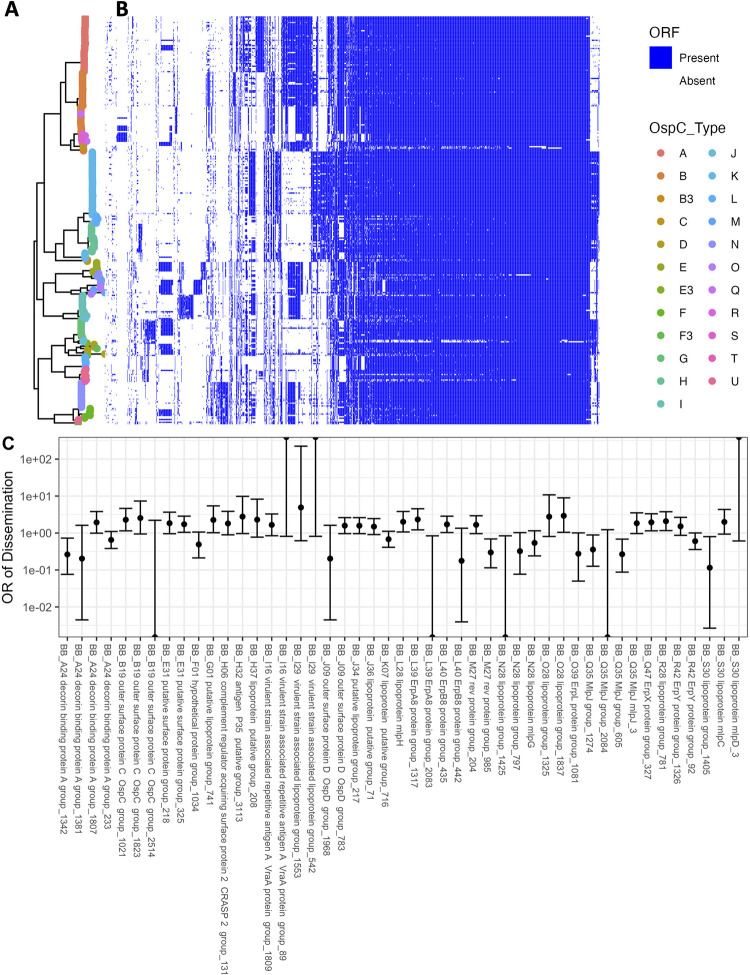
**A.** Core genome phylogeny with tips colored by OspC type. **B.** The phylogeny is plotted alongside a matrix of presence (blue) or absence (white) for genes in the accessory genome. The rows of the matrix are ordered by the phylogenetic tree in A. The columns of the matrix are ordered using hierarchical clustering such that genes with similar patterns of presence/absence across the sequenced isolates are grouped close together. **C.** Odds ratio (OR) of dissemination and 95% confidence interval for homology groups encoding surface-exposed lipoproteins and for which the unadjusted p-value for association with dissemination (by Fisher’s exact test) is < 0.15.

In an effort to implicate individual genetic elements in dissemination, we identified the core and accessory genome elements in each of the sequenced isolates and annotated and clustered all ORFs in the *de novo* assemblies using BLAST, splitting clusters whose BLAST homology was < 95% (Fig 5). Plotting the presence or absence of a given core or accessory genome element adjacent to each isolate in the phylogeny reveals consistent patterns of ORF presence/absence across closely related groups of isolates. Each of the genomic groups contained unique clusters of ORFs in the accessory genome (Fig 5). The accessory genome phylogenetic tree provided an alternative and more natural clustering of accessory genome elements and PFam32 sequences (S6A and S6B Fig).

**Fig 6:**
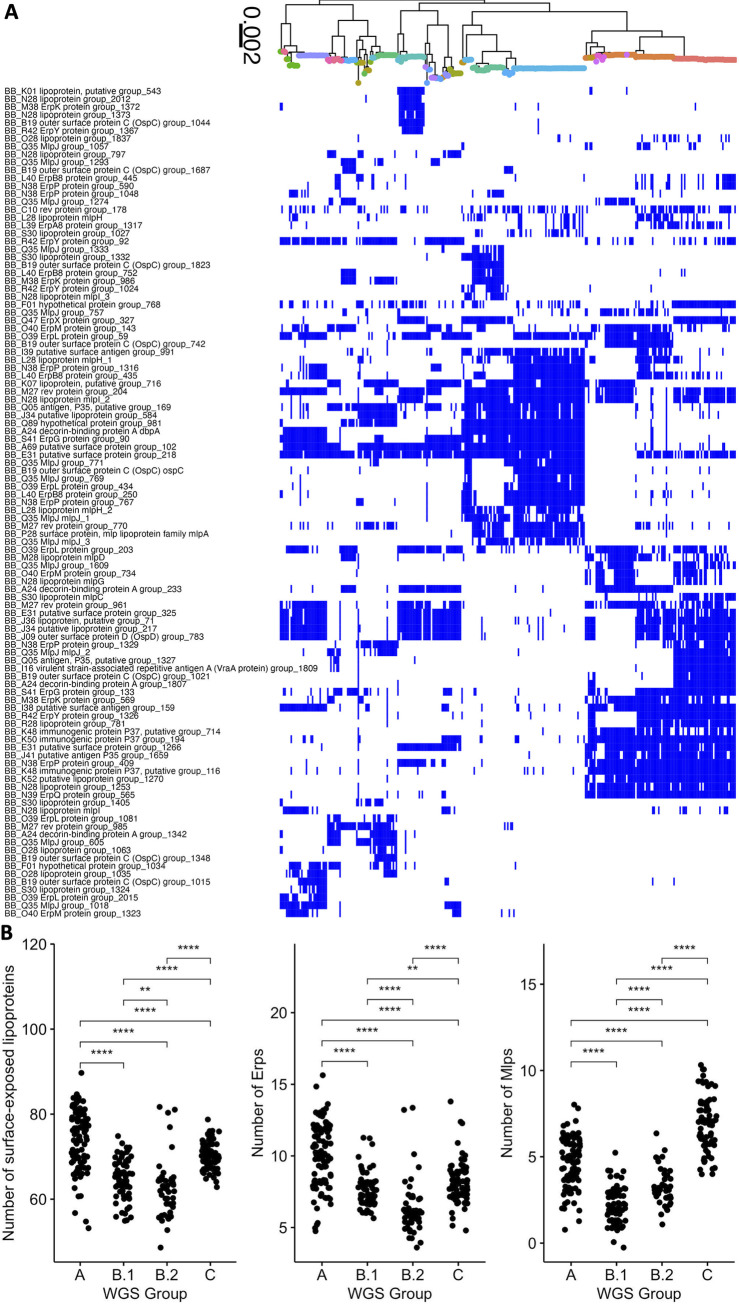
**A.** *Bb* strain-variable (accessory) surface lipoproteome: Core genome phylogeny with tips colored by OspC type (colored according to the scheme in Fig 5) with a matrix of presence (blue) or absence (white) for surface lipoproteins. Surface-exposed lipoproteins present in between 5% and 80% of strains were considered to be part of the strain-variable (accessory) lipoproteome. B. The number of surface-exposed lipoproteins (left panel), Erps (middle panel), and Mlps (right panel) by WGS group. ** denotes p < 0.01; *** denotes p < 0.001; **** denotes p < 0.0001, as assessed by Wilcoxon rank-sum test.

**Fig 7:**
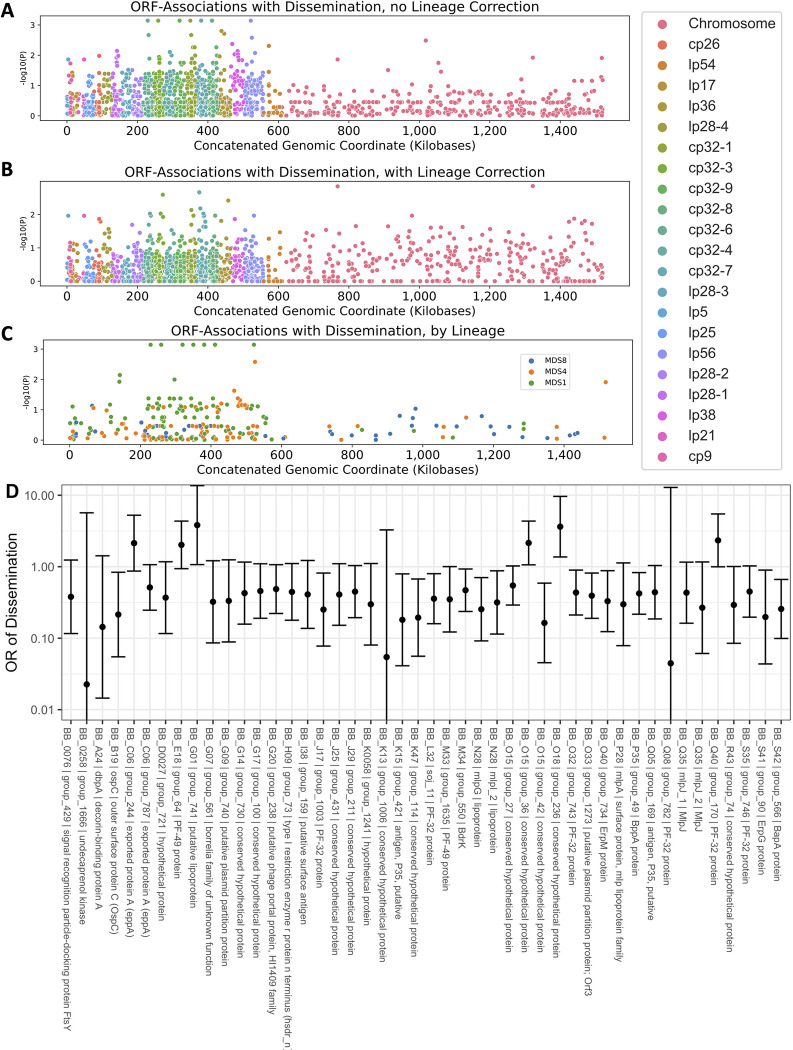
Manhattan Plots showing the association of individual ORF homology groups with the phenotype of dissemination. The Y axis plots the P-value for tests of association between each homology group and the phenotype of dissemination are shown. For ORFs that aligned to the B31 reference genome, the x axis denotes the annotated position in the genome. **A.** P-values from univariate logistic regression by genomic position for each ORF. **B.** P-values from regression estimates that include lineage correction. **C.** Manhattan plot showing loci associated with each lineage for the lineages associated with phenotype. **D.** Odds ratios (OR) (exp(beta)) with 95% confidence interval are shown for dissemination for the lineage-adjusted model. ORFs with p < 0.1 and allele frequency > 0.1 and < 0.9 are displayed.

We prioritized surface-expressed lipoproteins (Figs 5C and 6) for further analysis because of their important roles in Lyme disease pathogenesis and immunity (reviewed in [1,62]). We focused on the subset of all lipoprotein ORFs demonstrated to be located on the surface of the spirochete [63] and divided them into core (S7A Fig) and strain-variable (Fig 6A). The *Bb* core lipoproteome (S7A Fig) consists of approximately 45 surface lipoprotein groups that are present in almost every isolate. These include OspA and B, complement regulator acquiring surface proteins (CRASPS), as well as several other lipoproteins whose functions are less well-understood. The accessory lipoproteome (Fig 6A) consists of approximately 100 lipoprotein groups that are strain-variable. These include lipoproteins found in only subsets of isolates, such as BB_A69 and BB_E31, and others, such as Decorin binding protein A (BB_A24) and OspC (BB_B19), which we found in almost every isolate but broken into separate ortholog groups because of extensive allelic diversity. Strain-specific clusters were also present in major gene families of Erps [64,65] (S7B Fig) and Mlps [66,67] (S7C Fig). We found larger numbers of these multi-gene family members in more invasive WGS groups (A and C) (Fig 6B). The number of lipoproteins in a given isolate was associated with the probability of dissemination (β_1_ = 0.037 +/- 0.017, p = 0.03, logistic regression, Fig 7E). We observed a stronger effect for Erps (β_1_ = 0.087 +/- 0.053, logistic regression, Fig 7E) with a trend toward significance (p = 0.1). In contrast, the total number of ORFs and the number of Mlp alleles were not significant in logistic regression models (p = 0.45 and p = 0.38, respectively, Fig 7E). Aggregating mean effects by OspC types (S7F Fig) showed similar trends to individual isolates, i.e. OspC types with greater numbers of lipoproteins were more likely to disseminate.

Several lipoprotein groups, such as BBK32, BBK07, and BBK52 were found in almost all strains, but were not found in a subset of closely related genotypes. Notably, CspZ (BBH_06) and two other lipoproteins encoded on lp28-3, BB_H37 and BB_H32, were lost in two divergent subsets of Slovenian isolates (S7A Fig), suggesting multiple independent loss events in evolutionary history. Interestingly, these two subsets were either WGS-A or WGS-B.2, strains with the greatest and least probability of dissemination (S3 Fig). The increased frequency of loss of lp28-3 in Slovenian isolates implies that this plasmid is likely non-essential for human infection.

Many genes had evidence of recurrent loss or gain. For example, one cluster that shows this pattern in Fig 5B contains the lipoproteins BB_J45, BB_J34, and BB_J36 along with 12 other genes annotated on the lp38 in B31, suggesting that these lipoproteins had been lost or gained multiple times in the evolutionary tree as a part of a pattern that involved most or all of lp38.

### Associations between accessory genome elements, Genotype, and dissemination

The genetic basis of the phenotypic differences between these strains most likely includes nucleotide-level variation in chromosomal and plasmid DNA as well as variation in gene presence or absence in the accessory genome (which is primarily plasmid-borne). While it is not feasible to resolve these associations definitively in this study, we attempted to identify preliminary ORF-level associations by clustering ORFs according to homology using Roary [49]. We then applied linear mixed models genome-wide study approaches to identify homologous groups of ORFs associated with disseminated infection (Fig 7A and 7B). We used the approach of Lees et. al [68] to adjust for lineage effects by identifying lineages that were associated with a phenotype.

Three lineages, defined by principal components of the distance matrix between isolates, were significantly associated with the phenotype of dissemination (MDS1, p = 0.004, MDS2, p = 0.03, and MDS8, p = 0.04, Wald’s test). In ancestry-adjusted association logistic regression analysis in which principal components were included as covariates [69], only a handful of loci were associated with phenotype, and their genomic position was distributed throughout the genome with no strong spatial pattern (Fig 7B). The uncorrected association statistics showed somewhat stronger correlations that were concentrated in the plasmids (Fig 7A). The results of all analyses are reported in S7 Table and lipoprotein-specific analyses in S8 Table.

**Fig 8:**
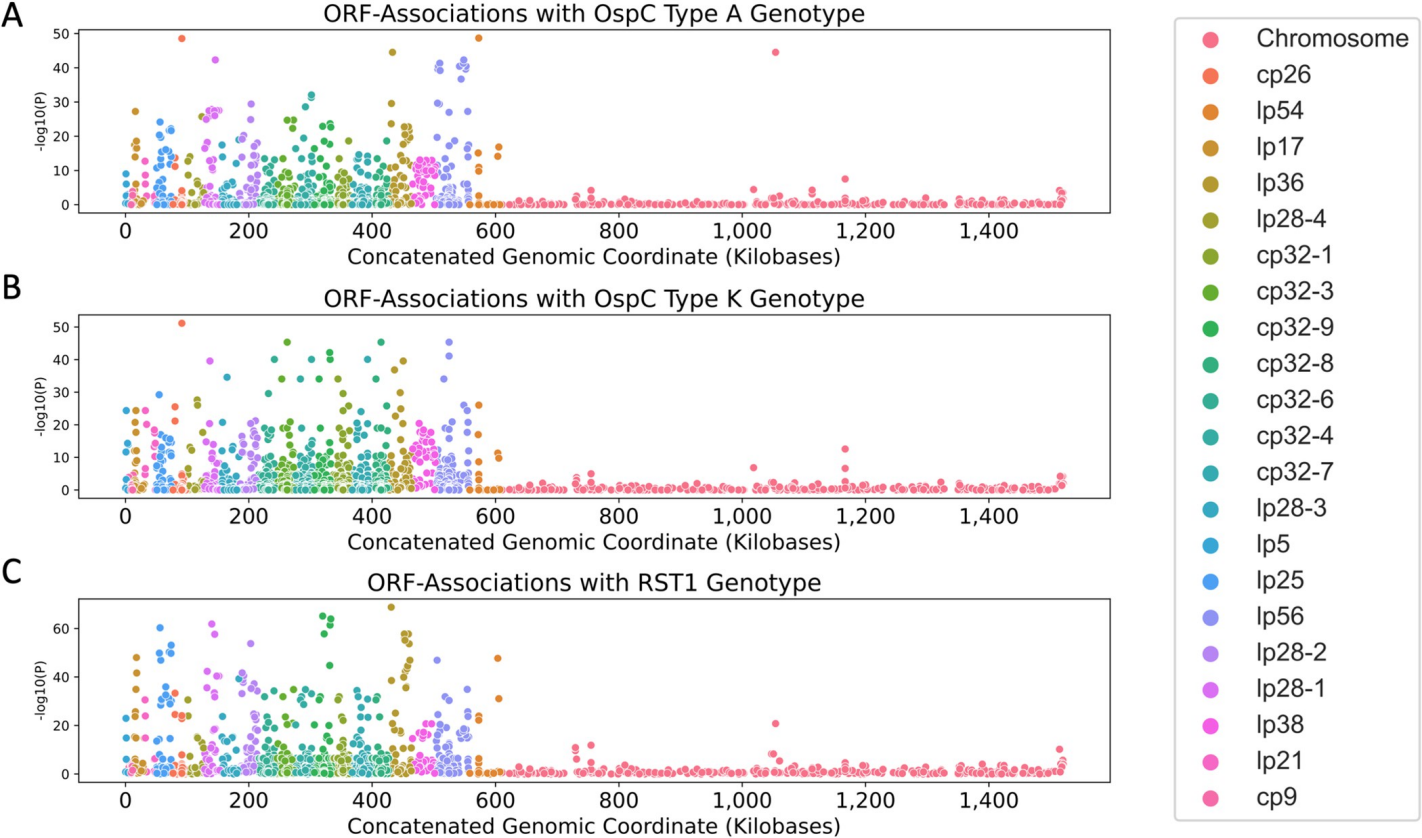
Manhattan Plots showing the association of individual ORF homologous groups with OspC type A (panel **A**), Osp C type K (panel **B**), and RST1 (panel **C**). The Y axis plots the P-value for tests of association between each homology group and the lineage marker as shown. For ORFs that aligned to the B31 reference genome, the x axis denotes the annotated position in the genome.

We also used the pan-genome association approach to identify associations between ORF homology groups and single-locus genetic markers. Single-locus genetic markers were strongly linked to genetic variation in ORF homology groups, particularly those ORFs encoded on plasmids (Fig 8; S9 Table for OspC Type A; S10 Table for OspC Type K; S11 Table for RST1). The strongest effects were seen among surface-exposed lipoproteins [63] (S8 Fig). Together, these results, along with those found in Fig 6, demonstrate that individual *Bb* genotypes represent tightly-linked sets of genes that confer distinct surface lipoproteomes.

Due to the structural patterns of genetic diversity in *Bb*, ORFs associated with phenotype without ancestry correction (Figs 5C and 7A) should not be ignored. Due to the near-complete linkage (e.g. Fig 8) between genetic elements in the accessory genome, individual loci with strong, causal effects on a given phenotype may not be separable from their set of linked variants, i.e. their background lineage. OspC type A strains, which have the highest rates of dissemination in this study (S4 Fig) and previous reports in mice and humans [6,7], and have been linked to more severe symptoms of Lyme disease [6] (S4C Fig), are strongly associated with a set of approximately 75 loci (OR > 50) including a DbpA homology group (OR 4964, p = 2.1 x 10^−49^, likelihood ratio test), an OspC homology group (OR 2951, p = 2.9 x 10^−49^, likelihood ratio test), and BB_H26 (OR 2186, p = 6.3 x 10^−40^, likelihood ratio test) (S9 Table). These and other linked alleles were strongly correlated with one another (r = 0.94, p < 2.2 x10^-16^ for DbpA/group1807 and OspC/group1021; r = 0.85, p < 2.2 x 10^−16^ for DbpA/group and BB_H26). In many cases this linkage is physical due to presence on the same replicon (e.g. the BB_J alleles on lp38), but strongly linked allelic groups may also be present on distinct replicons (e.g. DbpA on lp54 and OspC on cp26). While the strong correlations between individual alleles make it difficult to separate the statistical effects of individual alleles, such correlations are also the characteristic and defining feature of *Bb* lineages.

## Discussion

The sequencing and analysis of 299 human *Bb* clinical isolates adds insight to the genetic, geographic, and phenotypic diversity of *Bb* strains causing Lyme disease in several ways. First, our results confirm and extend previous findings on the microbial genetic basis of disease manifestations in humans. The surprising quality of single-locus typing systems for capturing the relevant genetic structure of *Bb* derives from the near-clonal population and resulting strong linkage among *Bb* genetic elements, a phenomenon which was observed in previous studies [70,71] and which was similarly observed in this large collection of human isolates.

Second, WGS goes beyond single-locus typing systems by revealing the specific genetic elements that contribute to strain-specific genetic and phenotypic variation. The presence of homoplasy among a subset of accessory genome elements (i.e. genes that are present or absent in multiple branches of the phylogeny in Fig 5B) means that single-locus markers are an imperfect proxy for strain-specific genetic differences. Thus, association studies linking genotype to phenotype benefit from WGS typing. In addition, while highlighting the fidelity and usefulness of single-locus typing, WGS also reveals their limitations. For RST, the main limitation is that marker subtypes are polyphyletic with respect to the core genome phylogeny, although the intermixing of WGS groups B and C in RST types 2 and 3 has not been a major issue in practice because the phenotypes (for example, the relative rate of dissemination in humans) of those groups appear more similar than the genomically and phenotypically divergent RST1 / WGS A group. For OspC typing, the main limitation is that there are many types, and the proliferation of closely-related subtypes reduces power in genetic association studies; furthermore, because of the frequency of recombination at the OspC locus [72,73], the distance between OspC sequences is not a reliable measure of the distance between strains. Unless there is a clear order or distance among the types (a condition which is not met by OspC types), the usefulness of a discrete typing system declines as the number of types increases.

Prior studies have identified genetic markers and correlated their presence with specific clinical findings [1,6,8,9,11,30,31,33–36]. Our findings support the idea that WGS A / RST1—particularly the subtype defined by OspC type A—is genetically distinct [31,71,74,75] and associated with an increased probability of dissemination in humans. We identified specific genetic features associated with this lineage, including having a larger number of ORFs than other lineages. These ORFs are found on a strain-specific collection of plasmids, including lp28-1 and lp56. This is consistent with previous findings that have linked the presence of lp28-1 to infectivity in mouse models [76–79]. Importantly, these results extend previous findings which showed that RST1 OspC type A strains are associated with more severe Lyme disease [6], by identifying candidate plasmids lp28-1 and lp56 as potential genetic factors that mediate the greater virulence of these *Bb* genotypes in patients.

Why are OspC type A strains more virulent? While an association does not establish causality, we report here that lipoprotein number is associated with the probability of invasion; we speculate that the larger collections of surface lipoproteins in virulent strains such as OspC types A and H may enable such spirochetes to defend more effectively against the host immune response or invade host tissues. Surface lipoproteins are known to be important in immunity, pathogenesis, and *Bb-*host interactions (reviewed in [1,62]).

Both gene dosage and allelic variation among lipoproteins present in the same quantity may be important. For example, at the level of allelic variation, distinct homology groups of OspC and DbpA are associated with the OspC type A genotype in this study. Previous experimental work has shown that specific allelic variants of DpbA promote dissemination and alter tissue tropism in a mouse model of Lyme disease [80]. Moreover, allelic variation in OspC alters binding to extracellular matrix components, promotes joint invasion, and modulates joint colonization [81]; OspC has also been shown to bind to plasminogen [82,83], promote resistance in serum killing assays [84], and its role in causing infection can be, under certain circumstances, partially complemented by other surface lipoproteins [85,86]. Homology groups of DbpA (BB_A24), and specific members of the Erp (BB_M38, BB_L39) and Mlp (BB_Q35) (S8 Table and S7B–S7D Fig) families are associated with dissemination, and the genetic differences among these homology groups represent potential candidates for evaluation in follow-up studies.

At the level of gene dosage, differences were particularly notable among multi-copy gene families such as Erps and Mlp proteins. The statistically-significant relationship between lipoprotein number and probability of dissemination in humans and the borderline-significant relationships for copy number of Erps and Mlps (S7E and S7F Fig) suggest that varying the amount and diversity of linked clusters of surface lipoproteins—which, individually or in combination, may promote survival in the presence of immune defenses, binding to mammalian host tissues and through other pathogenic mechanisms—may be a general mechanism to facilitate vertebrate infection and, consequently, may underlie strain-specific virulence of *Bb* in humans. Erps are divided into three families that each bind to distinct host components (extracellular matrix, complement component, or complement regulatory protein) [65,87–90]; it is possible that the strain-variable clusters of Erps (S7B, S7E and S7F Fig) may influence clinical manifestations by modulating strain-specific properties of tissue adhesion or resistance to complement-mediated killing of spirochetes. The functions of Mlp proteins and many other strain-variable lipoproteins remain largely unknown.

The microbial genetic association studies presented here begin to resolve the individual genetic elements underlying certain phenotypes of Lyme disease. We hypothesized that specific genetic elements were associated with dissemination in patients. Our findings support this hypothesis by identifying groups of genes associated with dissemination in humans, but due to the near-clonal population structure of *Bb*, it is not possible to resolve the specific genetic elements within these groups without further investigation. Using unadjusted, univariate associate models, virtually all dissemination-associated genes were found on plasmids. However, after correction for spirochete genetic structure due to lineage, only weak locus-specific associations were observed. Distinguishing causal alleles from non-causal, linked alleles requires statistical reassortment, usually in the form of recombination, and/or experimental data. Because reassortment does occur (lineages are not perfectly clonal), larger sample sizes can help narrow the list of potential causal loci. Improved statistical models that explicitly incorporate the joint distribution of covariates among isolates would also help. In the near term, until much larger collections of isolates are available, pinpointing causal alleles will depend on experiments using reverse genetic tools. The results shown in Figs 6 and 7 and S7 Table are helpful in narrowing down the candidate loci and genetic elements that may predispose to or protect from dissemination in humans.

The complex structure of the *Bb* genome further complicates the identification of causal loci because the genes in dissemination-associated clusters are predominantly found on plasmids. Integrating plasmid maps with associations at the level of individual ORFs provides a clearer view of the potential determinants of distinct phenotypes. While we cannot yet resolve the causative loci on lp28-1 or lp56 that enhance the pathogenicity of OspC type A strains, we highlight candidate loci and quantify the statistical evidence for each locus considered. ORFs on these plasmids such as BB_Q67 (which encodes a restriction enzyme modification system [91,92]), BB_Q09, BB_Q05, BB_Q06, BB_Q07, and other plasmids such as BB_J31, BB_J41 (S7 and S8 Tables) are among tightly linked to the OspC type A genotype and are candidates for further experimental examination. However, without complete plasmid sequences, the spatial context of these associations and the physical structure of linkage are not resolved. Long-read sequencing will be necessary to define these relationships and establish a definitive map of plasmids because of the frequent, complex exchanges of genes and gene blocks among plasmids [14,16].

Finally, our analysis highlights how strain genetic diversity, which is shaped by geographical location and evolutionary history, contributes to clinical heterogeneity in Lyme disease. In the context of known associations between genotype and clinical disease, the differences in genetic markers across geographic areas may help explain why some clinical phenotypes are more common in certain geographic locations. For example, Lyme arthritis is more common in the US compared to Europe, probably because the infection in the US is due predominantly to *Bb* strains which are more arthritogenic [93]. OspC type A strains appear to be more common among patients in the US Northeast [30,31].

This report has several limitations. First, plasmids pose a unique challenge for assembly and annotation [14,16]. As others have shown [17], complete plasmid assembly with short read sequences is not possible. We devised two bioinformatic methods to overcome these challenges and infer plasmid presence/absence from short read sequencing, but neither is perfect. Our PFam32 analysis is limited by an uncertainty as to which gene sequences are contained on the plasmid associated with the PFam32 sequence [16]. A complementary analysis based on the B31 reference sequence relies on a high-quality pre-existing assembly but cannot account for genes/plasmids absent from the B31 reference strain. We also cannot exclude the possibility of plasmid loss during culture, although isolates were passaged fewer than five times before genome sequencing to minimize this possibility.

Second, there are limitations due to analysis of isolates collected over time by different groups at different sites. In particular, we may underestimate dissemination because an assessment of spirochetemia (blood PCR or blood culture) was only available for 71% of isolates (S2 Table) and the absence of positive culture or blood PCR from a single time point does not rule out the possibility that dissemination from the initial skin lesion may have occurred or may occur at a later time point if patients were not treated with antibiotics. Further, because we did not genotype blood isolates for this study, we cannot rule out that the strain that disseminated to blood was different than those cultured from the EM skin biopsies. However, based on past experience at NYMC, where skin and blood cultures were frequently obtained from the same patient, the majority (>90%) of *Bb* genotypes recovered from blood matched those in skin (personal communication: I.S. and G.W.).

Third, there are statistical limitations related to the *Bb* genome and study size. We did not study all types of genetic variation. In particular, copy number variants (CNVs) and single nucleotide polymorphisms (SNPs) were not considered here. Short read methods are not ideal for studying CNVs. SNPs are incorporated indirectly through the measure of overall sequence similar (BLAST identity) used to split homologous group clusters, but a detailed association study of SNPs requires a larger sample size which is not currently available.

Fourth, models that naively correlate a given gene with the phenotype of interest will produce spurious associations due to the confounding effect of lineage and may overstate the effect from single loci, a problem which is well known in human genome-wide association studies [94]. Corrections for lineage and population structure are often applied to human [95,96] and bacterial [68,69] association studies. However, *Bb* underscores the challenges to these approaches, both because lineages appear to be *defined* by the exchange of blocks of genes rather than single genes, and because the coarse tree structure differs for the core and accessory genomes, implying that a single similarity measure to capture the pairwise dissimilarity between strains may not be adequate. Larger studies with more isolates, statistical methods that incorporate the joint distribution between genetic markers, and plasmid assemblies finished by long read sequencing are required as a next step. Until complete assemblies are available, we regard plasmid assignment for each strain as provisional because both of the methods we used to infer the presence/absence of plasmids have limitations related to the extensive homology among plasmids and the imperfect linkage between PFam32 sequences and the other genes on the plasmid [16].

Fifth, the present study includes isolates collected by different investigators over the past 30 years. Due to the logistical complexity and cost of collecting *Bb* isolates from patients in clinical studies, substantially larger studies of isolates of *Bb* from patients may not be feasible in the near term; however, long-read sequencing approaches have improved in accuracy, availability, and cost, and are a logical next step to completing the genomes of existing isolates in our collection.

Taken together, our results indicate that each *Bb* genotype represents a tightly-linked set of strain-specific variation that occurs primarily in plasmids, much of it involving surface-exposed lipoproteins. OspC type A strains—with their enlarged pan-genome, distinct plasmids, and expanded surface lipoproteome—represent the most dramatic example of this genetic signature that is associated with distinct phenotypes of Lyme disease in humans. Given the shared principles of genome organization and strong linkage between microbial genotype and phenotype across all Lyme borrelia, this pattern may generally be true for all agents of Lyme disease.

## Supporting information

S1 Table Summary table of isolates and phenotypes. (DOCX)

S2 Table List of isolates and phenotypes. (TSV)

S3 Table Assembly statistics. (TSV)

S4 Table Contingency tables and association statistics for dissemination. (CSV)

S5 Table Association statistics for plasmids, as inferred from PFam32 types. (TSV)

S6 Table Association statistics for plasmids, as inferred from B31 reference. (TSV)

S7 Table Association statistics for lineage model. (CSV)

S8 Table Association statistics for lineage model restricted to surface lipoproteins. (CSV)

S9 Table Association statistics for OspC type A associations. (CSV)

S10 Table Association statistics for OspC type K associations. (CSV)

S11 Table Association statistics for RST1 associations. (CSV)

S1 File List of ortholog groups with reference sequences. (FA)

S2 File High resolution version of presence/absence matrix for accessory genome elements. (PDF)

S1 Fig Multidimensional scaling (MDS) analysis of the 299 *Bb* isolates with covariates colored according to A. OspC Type B. RST Type, C. Dissemination status, and D. Geographic region.
(PDF)

S2 Fig A. Maximum likelihood phylogenetic tree of core genome sequences. The tree was constructed using iqtree and ultrafast bootstrap support is labeled for nodes. Only nodes with bootstrap support > 90% are labeled. OspC types are displayed in color and also annotated with text. The region of collection and RST type are labeled by colored boxes adjacent to the tips. Dissemination status is denoted with a star (disseminated isolates) or square (localized). WGS group is labeled by colored points on the outer rim of the figure. The bootstrap support for all nodes > 0.9 has been labeled in blue text. The tree scale is in nucleotide substitutions per site. B. MCC WGS tree constructed using BEAST (left) and ML tree constructed with IQtree (right) with identical tips connected by strain lines, colored by WGS group. Internal nodes with posterior support > 0.9 (left) or ultrafast bootstrap support > 90% have been colored. C. OspC phylogenetic BEAST MCC tree with metadata annotated adjacent to the tips. OspC types are displayed in color and annotated with text. The region of collection and RST type are labeled by colored boxes adjacent to the tips. Dissemination status is denoted with a star (disseminated isolates) or square (localized). WGS group is labeled by colored points on the outer rim of the figure. The posterior support for all nodes > 0.9 has been labeled in blue text. The tree scale is in amino acid substitutions per site. D. BEAST MCC WGS tree (left) and ML WGS tree with identical tips connected by strain lines, colored by WGS group. Internal nodes with posterior support > 0.9 (MCC tree) and ultrafast bootstrap (UFBoot) support > 90% are labeled. The scale is in nucleotide substitutions; the scale on the right is in amino acid substitutions per site and has been reduced by a factor of 50 for visualization purposes.
(PDF)

S3 Fig A. Core genome phylogenetic tree colored by WGS groups A-C with group B divided into B.1 and B2; accessory genome presence/absence matrix is shown at right to highlight accessory genome elements that correlate with B.1 and B.2 sublineages. The clade corresponding to RST1 is shaded in light blue and the clade corresponding to OspC type A is shaded in green. B. MDS plot with group B divided into B.1 and B.2. C. Probability of dissemination by genomic group using the four groups including B.1 and B.2.
(PDF)

S4 Fig Probability of dissemination by (A) OspC type and (B) RST. C. Severity of Lyme disease by OspC type with WGS group shown by color.
(PDF)

S5 Fig Inferred presence / absence of a plasmid based on alignment of assembled contigs to the B31 reference. A plasmid is inferred as ‘present’ in the isolate if > 50% of the length is covered by aligned contigs in the de novo assembly for the genome of the corresponding isolate. The clade corresponding to RST1 is shaded in light blue and the clade corresponding to OspC type A is shaded in green. B. Odds ratio of dissemination and confidence interval by plasmid, inferred by PFam32 sequences. C. Volcano plot displaying the—log10 P value (as calculated using Fisher’s exact test) and the odds ratio of dissemination for each plasmid, inferred by alignment of assembled contigs to the B31 reference sequence.
(PDF)

S6 Fig A. Phylogenetic tree created from the accessory genome using Roary with accessory genome elements plotted according to their presence/absence in individual strains. B. Phylogenetic tree created from the accessory genome with PFam32 plasmid compatibility sequences plotted according to the presence/absence in individual strains.
(PDF)

S7 Fig A. *Bb* core surface lipoproteome: Core genome phylogeny with tips colored by OspC type (colored according to the scheme in Fig 5) with a matrix of presence (blue) or absence (white) for surface lipoproteins. Surface-exposed lipoproteins present in at least 80% of strains were considered to be part of the core lipoproteome. B and C. Core genome phylogeny with presence/absence of Erp (C) homology groups and Mlp (D) homology group. D. The number of surface-exposed lipoproteins (top panel), Erps (middle panel), and Mlps (bottom panel) by OspC type. E. Logistic regression modeling the probability of dissemination by number of ORF (top left, regression coefficient for slope, β1 = 0.002 +/- 0.002, p = 0.450), number of surface-exposed lipoproteins (top right, β1 = 0.037 +/- 0.017, p = 0.03, logistic regression), number of Erps (bottom left, β1 = 0.087 +/- 0.053, p = 0.10, logistic regression), and number of Mlps (bottom right, β1 = 0.048 +/- 0.055 p = 0.38, logistic regression). The observed data used to build the regression model are plotted. Each isolate is a point whose y-value has been assigned 1 to denote a disseminated phenotype or 0 to denote a non-disseminated phenotype. A small amount of noise has been added to the y-coordinate to display overlapping points. F. For each OspC type, mean probability of dissemination vs mean number of ORF (top left), mean number of surface-exposed lipoproteins (top right), mean number of Erps (bottom left), and mean number of Mlps (bottom right).
(PDF)

S8 Fig Manhattan Plots showing the association of individual lipoproteins with OspC type A (top panel), Osp C type K (middle panel), and RST1 (bottom panel). Individual lipoproteins are annotated by their localization. The scale is in 1,000,000 base pairs, with the ordering of plasmids and the chromosome as in Fig 7. P-IM: Periplasmic inner membrane. POM: Periplasmic outer membrane. S: surface.
(PDF)
