# Supplementary material for: Whole genome sequencing of human Borrelia burgdorferi isolates reveals linked blocks of accessory genome elements located on plasmids and associated with human dissemination
Source: PLoS Pathog. 2023 Aug 31;19(8):e1011243. doi: 10.1371/journal.ppat.1011243 (PMC10470944; doi:10.1371/journal.ppat.1011243)
Supplement: S1 Table — (DOCX) [file ppat.1011243.s001.docx]

| **Variable** | **Slovenia** | **US Northeast (CT, RI, MA)** | **US Northeast (NY)** | **US Midwest** | **Totals** |
| --- | --- | --- | --- | --- | --- |
| Total number of Bb isolates assessed | 36 | 113 | 88 | 62 | 299 |
| No. of patients with BCx neg | 26 | 0 | 43 | 0 | 69 |
| No. of patients with BCx pos | 3 | 0 | 34 | 0 | 37 |
| No. of patients with PCR neg | 0 | 66 | 0 | 0 | 66 |
| No. of patients with PCR pos | 0 | 40 | 0 | 0 | 40 |
| No. of patients with MEM absent | 32 | 92 | 72 | 37 | 233 |
| No. of patients with MEM present | 4 | 20 | 16 | 17 | 57 |
| No. of isolates from blood | 2 | 0 | 0 | 0 | 2 |
| No. of isolates from CSF | 4 | 0 | 0 | 5 | 9 |
| No. of isolates from skin | 30 | 113 | 88 | 57 | 288 |
| No. of patients with single EM and BCx NA | 6 | 92 | 10 | 37 | 145 |
| No. of patients with single EM and BCx neg | 25 | 0 | 40 | 0 | 65 |
| No. of patients with single EM and BCx pos | 1 | 0 | 22 | 0 | 23 |
| No. of patients with single EM and PCR NA | 32 | 6 | 72 | 37 | 147 |
| No. of patients with single EM and PCR neg | 0 | 58 | 0 | 0 | 58 |
| No. of patients with single EM and PCR pos | 0 | 28 | 0 | 0 | 28 |
| No. of patients with single EM and with PCR and BCx NA | 6 | 6 | 10 | 37 | 59 |

**Supplemental Table 1:** The number of isolates assessed by site of collection, method of collection, and clinical and laboratory markers of dissemination. Abbreviations: No. - Number, Bb - *Borrelia burgdorferi*, BCx - blood culture, pos - positive, neg - negative, EM - erythema migrans, MEM - multiple erythema migrans, PCR - polymerase chain reaction, NA - not available, CSF - cerebrospinal fluid.
