# Supplementary figures and images for: Whole genome sequencing of human Borrelia burgdorferi isolates reveals linked blocks of accessory genome elements located on plasmids and associated with human dissemination
Source: PLoS Pathog. 2023 Aug 31;19(8):e1011243. doi: 10.1371/journal.ppat.1011243 (PMC10470944; doi:10.1371/journal.ppat.1011243)

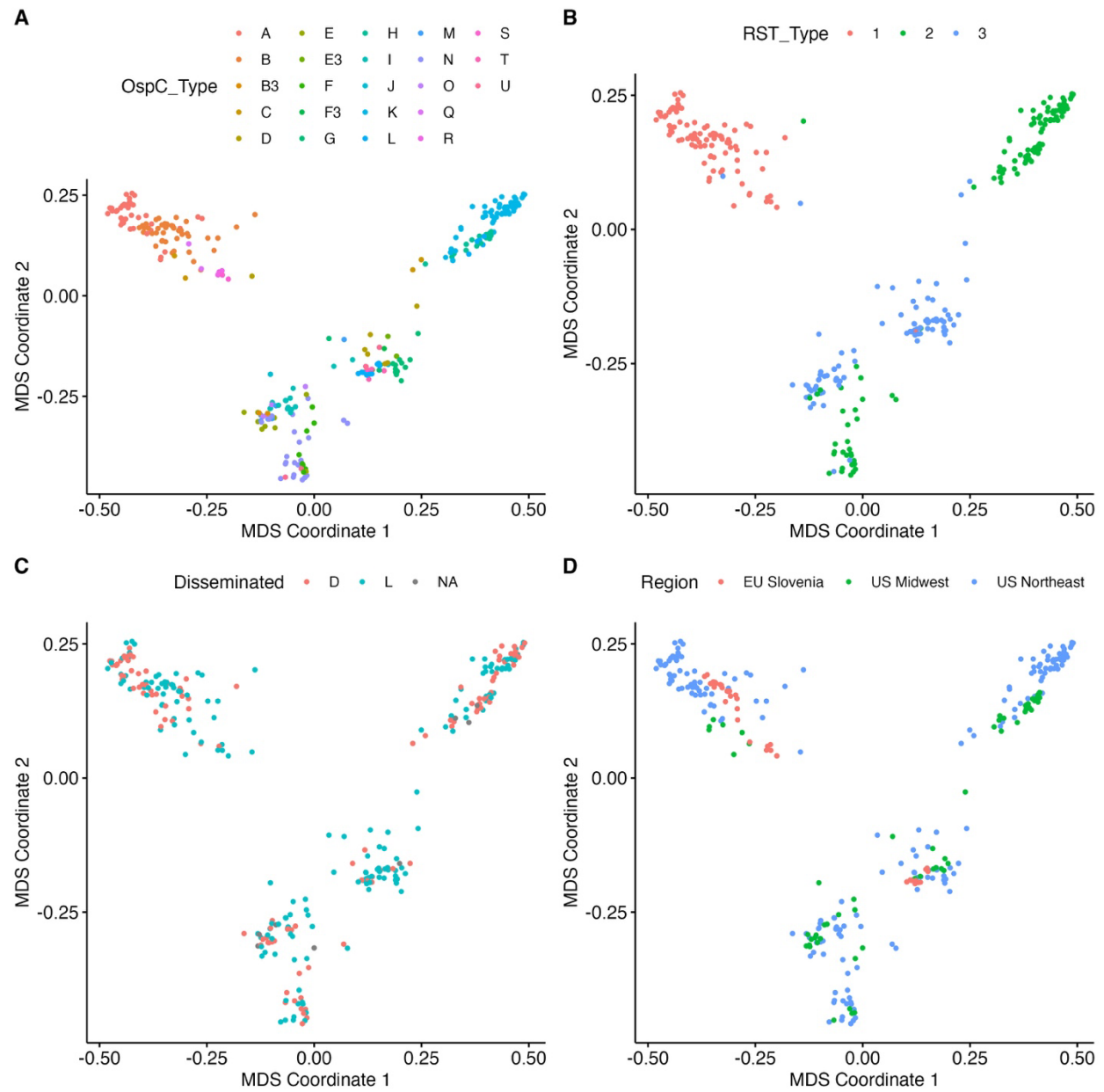

**S1 Fig**

Supplement: S1 Fig — OspC Type B. RST Type, C. Dissemination status, and D. Geographic region. (PDF) [file ppat.1011243.s014.pdf]

A

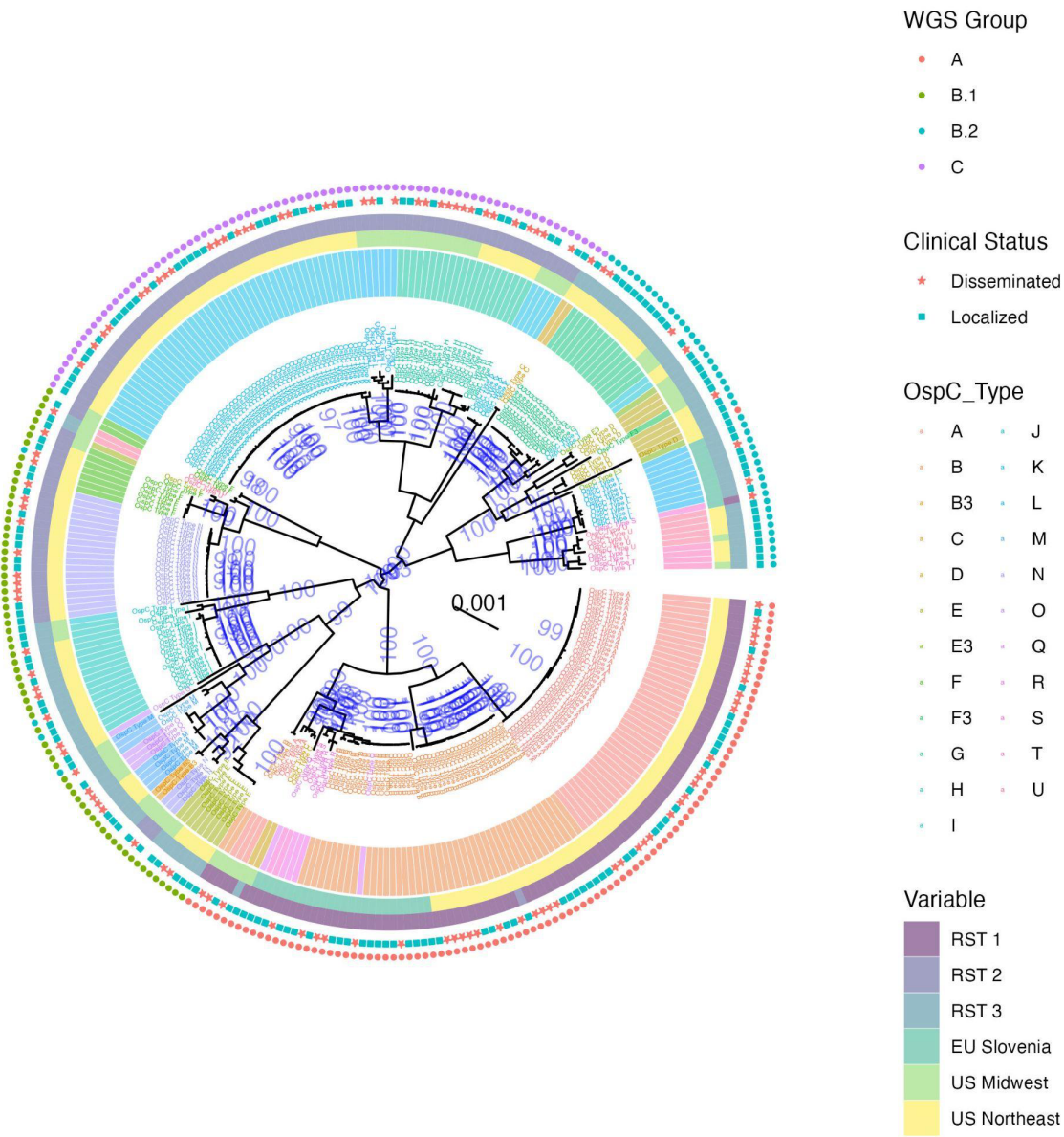

**B**

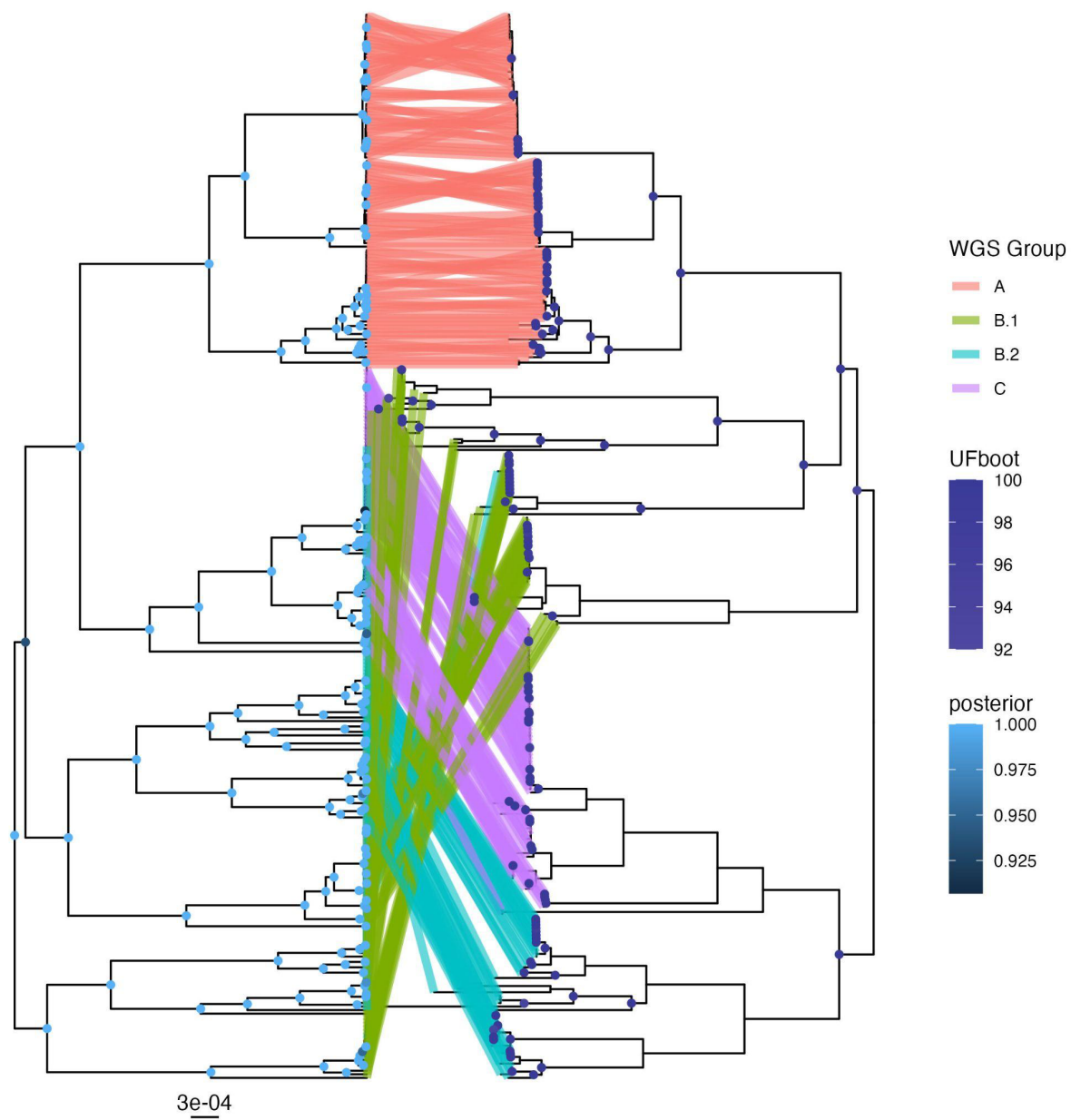

C

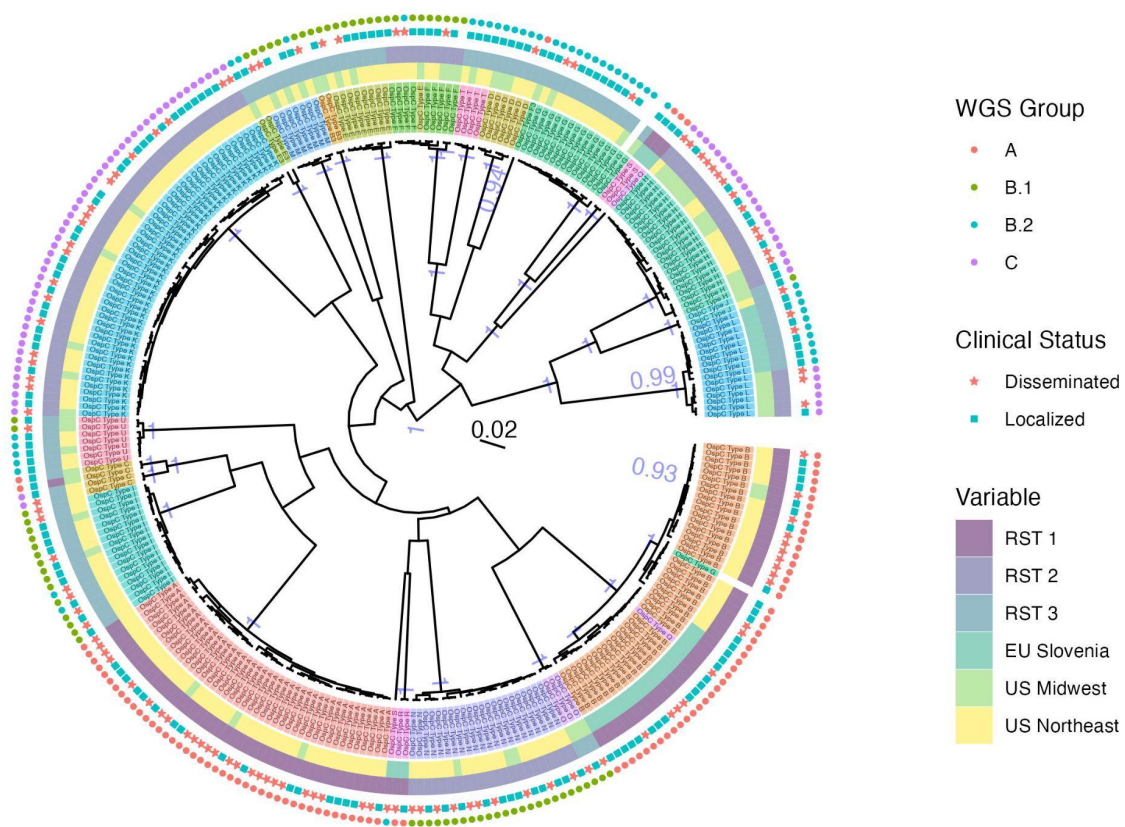

D

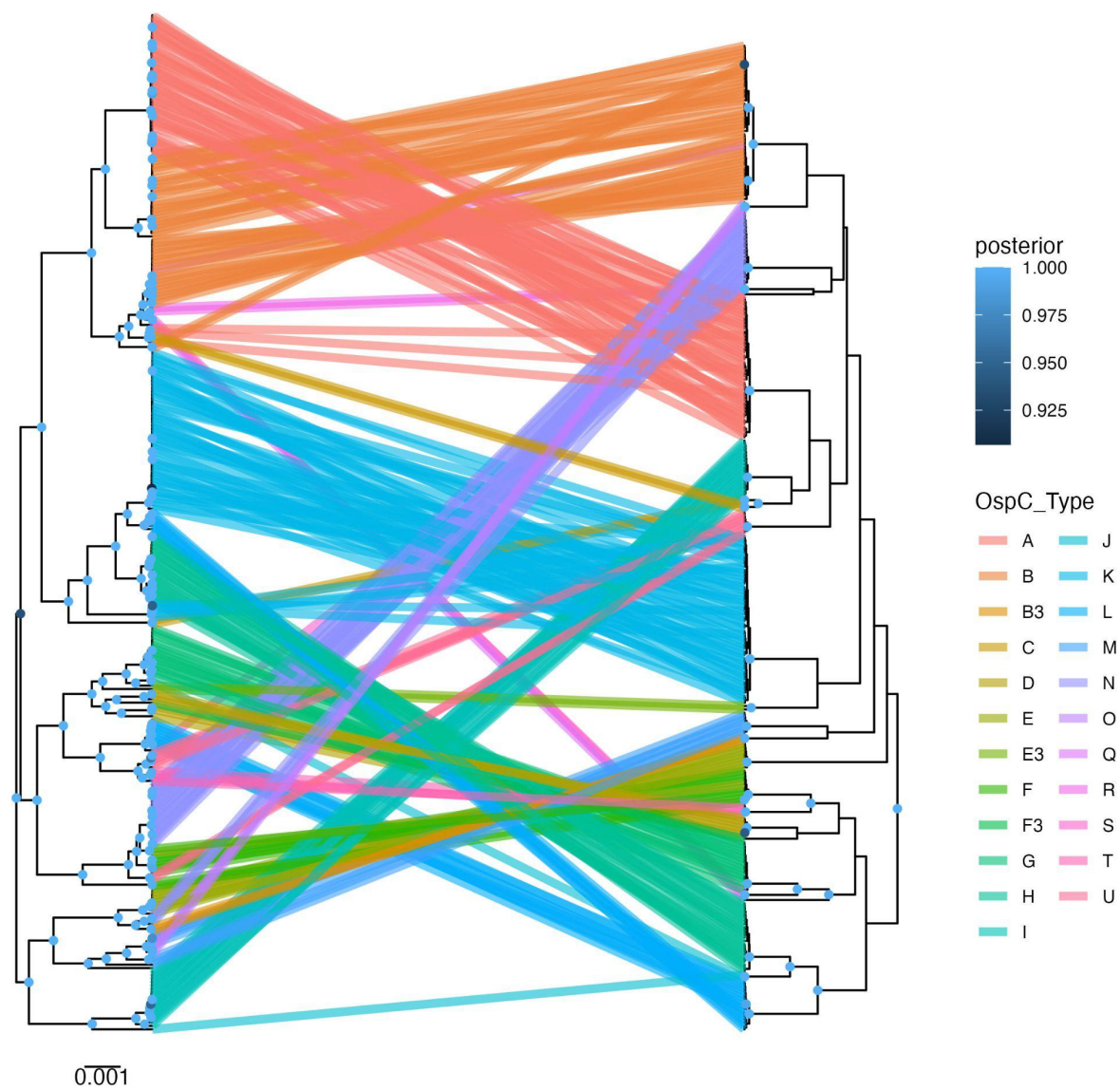

S2 Fig

Supplement: S2 Fig — A. Maximum likelihood phylogenetic tree of core genome sequences. The tree was constructed using iqtree and ultrafast bootstrap support is labeled for nodes. Only nodes with bootstrap support > 90% are labeled. OspC types are displayed in color and also annotated with text. The region of collection and RST type are labeled by colored boxes adjacent to the tips. Dissemination status is denoted with a star (disseminated isolates) or square (localized). WGS group is labeled by colored points on the outer rim of the figure. The bootstrap support for all nodes > 0.9 has been labeled in blue text. The tree scale is in nucleotide substitutions per site. B. MCC WGS tree constructed using BEAST (left) and ML tree constructed with IQtree (right) with identical tips connected by strain lines, colored by WGS group. Internal nodes with posterior support > 0.9 (left) or ultrafast bootstrap support > 90% have been colored. C. OspC phylogenetic BEAST MCC tree with metadata annotated adjacent to the tips. OspC types are displayed in color and annotated with text. The region of collection and RST type are labeled by colored boxes adjacent to the tips. Dissemination status is denoted with a star (disseminated isolates) or square (localized). WGS group is labeled by colored points on the outer rim of the figure. The posterior support for all nodes > 0.9 has been labeled in blue text. The tree scale is in amino acid substitutions per site. D. BEAST MCC WGS tree (left) and ML WGS tree with identical tips connected by strain lines, colored by WGS group. Internal nodes with posterior support > 0.9 (MCC tree) and ultrafast bootstrap (UFBoot) support > 90% are labeled. The scale is in nucleotide substitutions; the scale on the right is in amino acid substitutions per site and has been reduced by a factor of 50 for visualization purposes. (PDF) [file ppat.1011243.s015.pdf]

**A**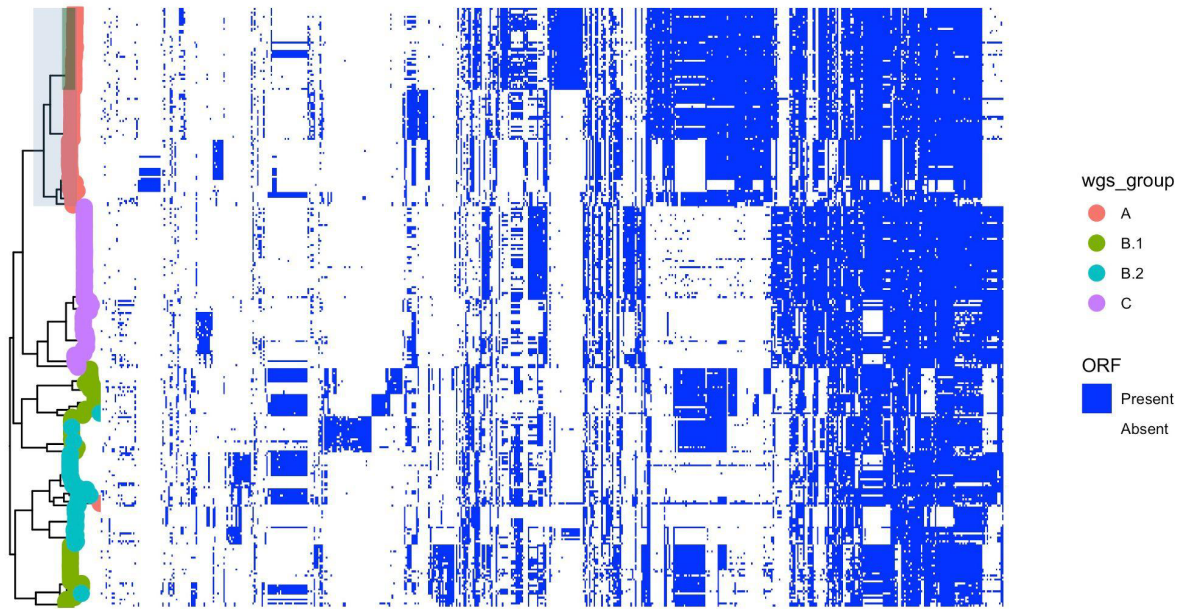**B**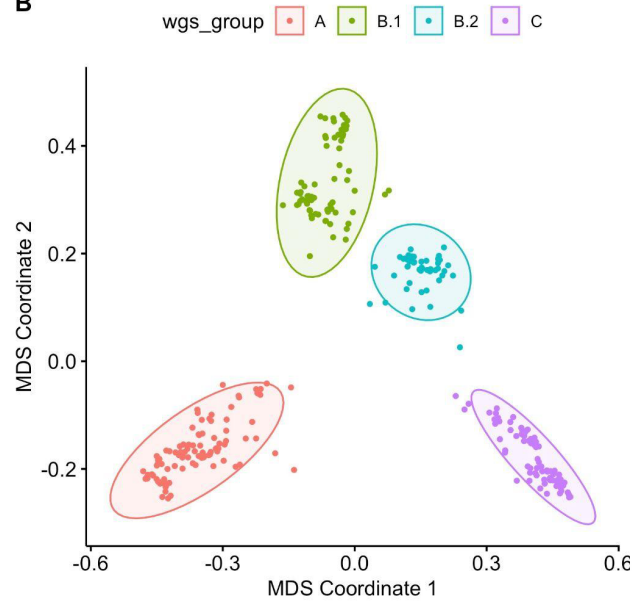**C**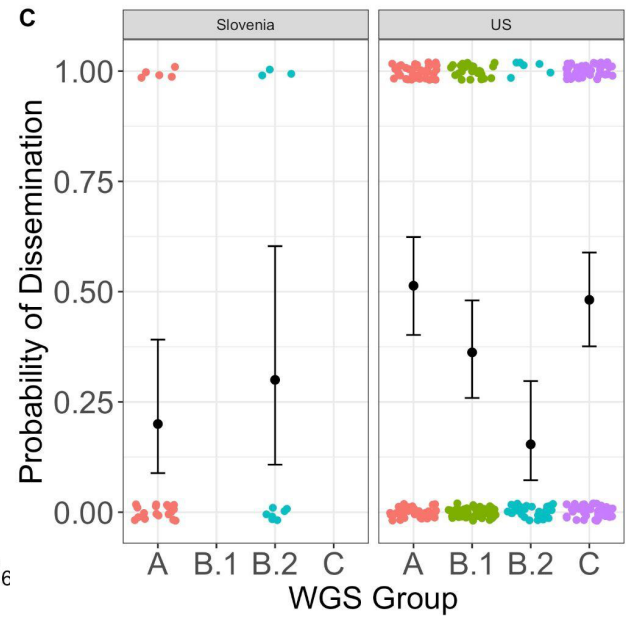**S3 Fig**

Supplement: S3 Fig — A. Core genome phylogenetic tree colored by WGS groups A-C with group B divided into B.1 and B2; accessory genome presence/absence matrix is shown at right to highlight accessory genome elements that correlate with B.1 and B.2 sublineages. The clade corresponding to RST1 is shaded in light blue and the clade corresponding to OspC type A is shaded in green. B. MDS plot with group B divided into B.1 and B.2. C. Probability of dissemination by genomic group using the four groups including B.1 and B.2. (PDF) [file ppat.1011243.s016.pdf]

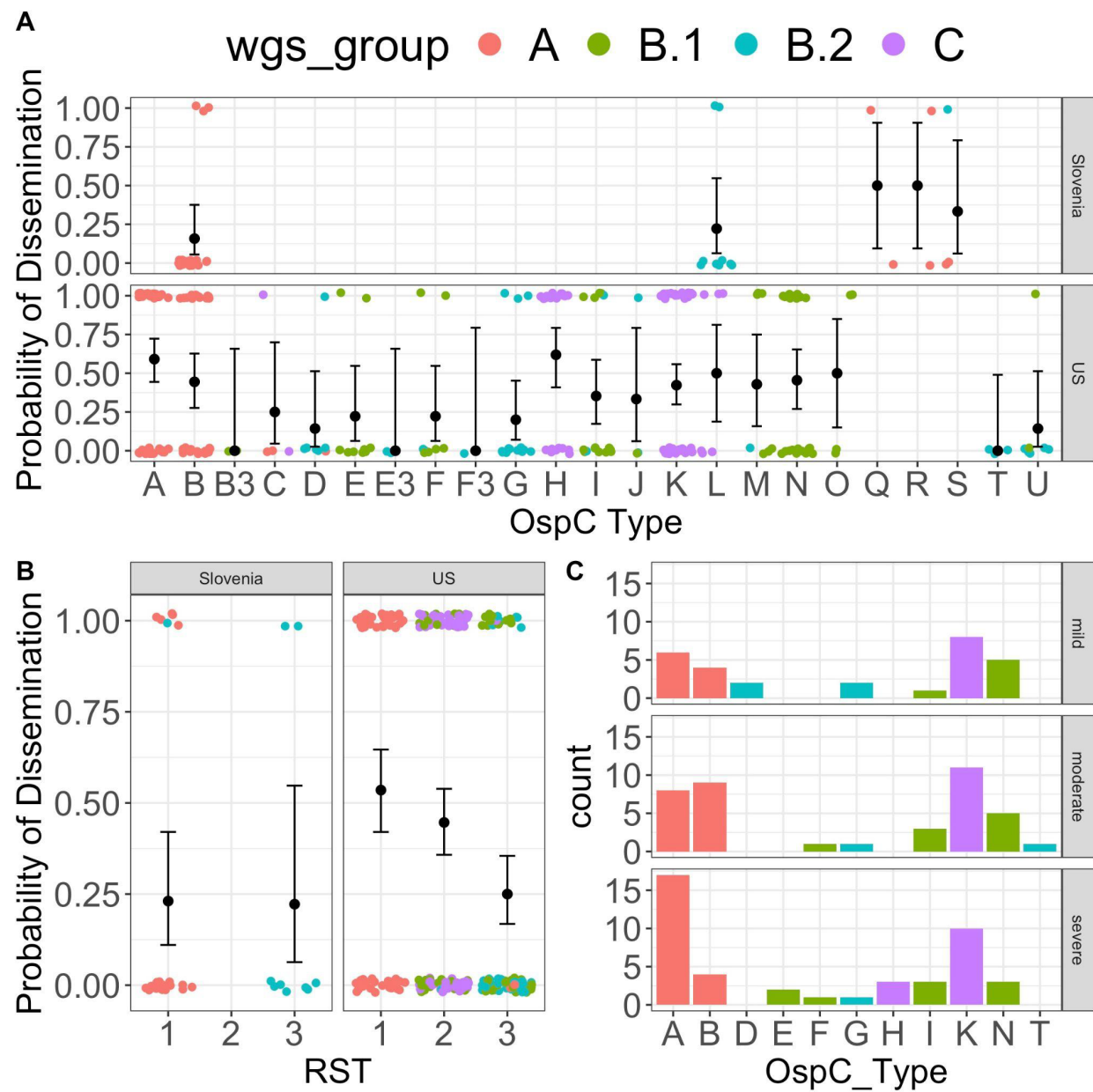

**S4 Fig**

Supplement: S4 Fig — Probability of dissemination by (A) OspC type and (B) RST. C. Severity of Lyme disease by OspC type with WGS group shown by color. (PDF) [file ppat.1011243.s017.pdf]

**A**

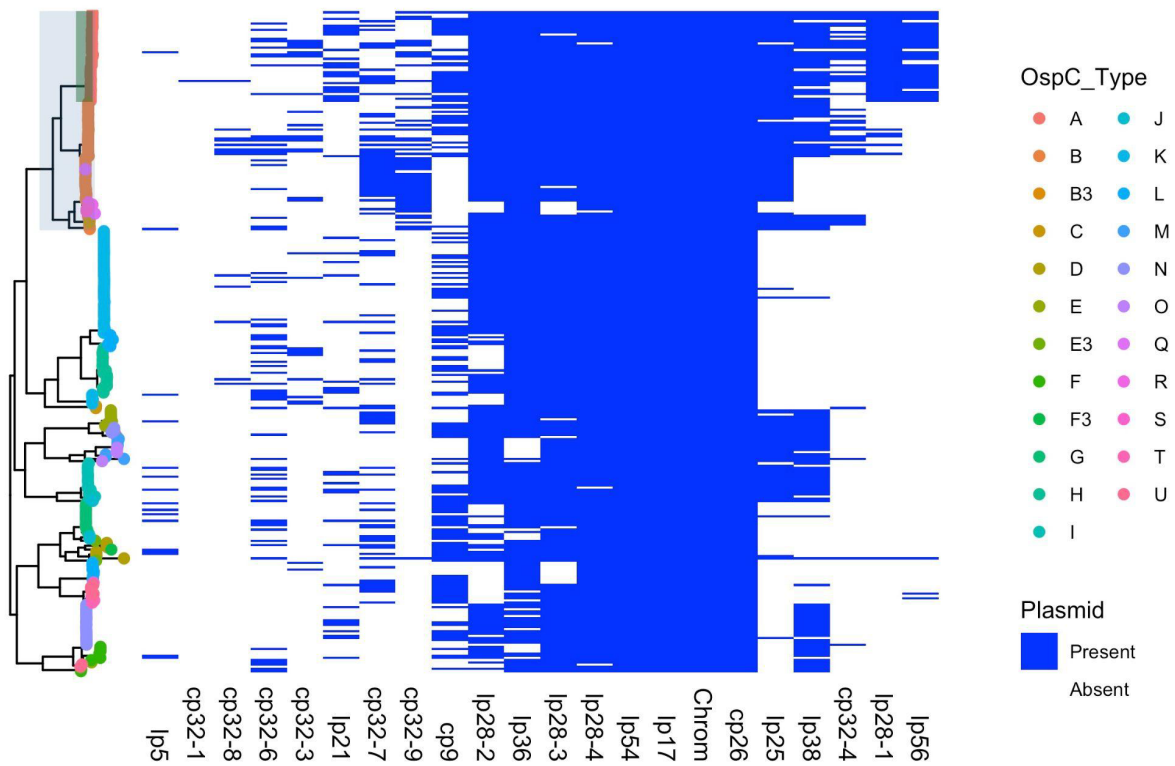

**B**

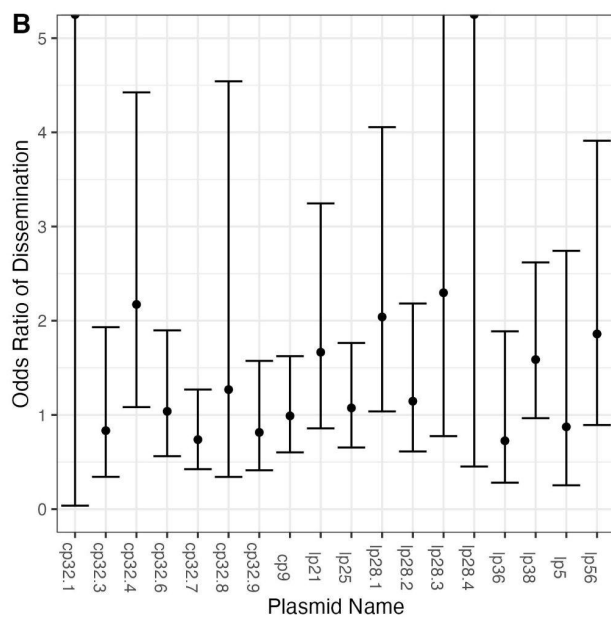

**C**

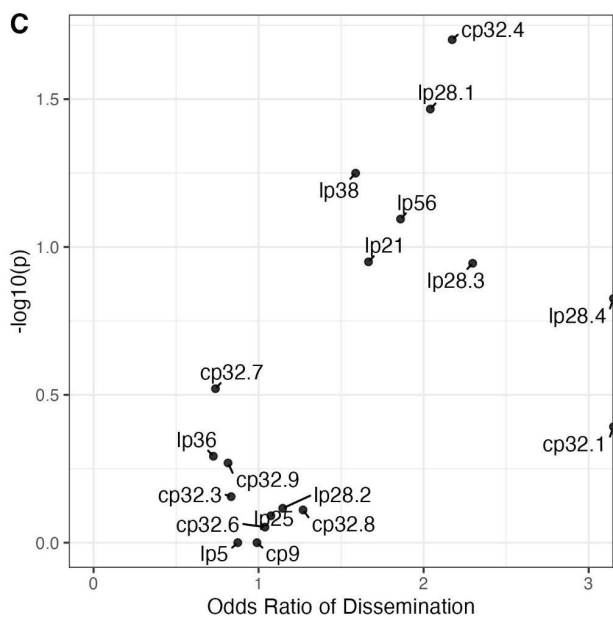

**S5 Fig**

Supplement: S5 Fig — A plasmid is inferred as ‘present’ in the isolate if > 50% of the length is covered by aligned contigs in the de novo assembly for the genome of the corresponding isolate. The clade corresponding to RST1 is shaded in light blue and the clade corresponding to OspC type A is shaded in green. B. Odds ratio of dissemination and confidence interval by plasmid, inferred by PFam32 sequences. C. Volcano plot displaying the—log10 P value (as calculated using Fisher’s exact test) and the odds ratio of dissemination for each plasmid, inferred by alignment of assembled contigs to the B31 reference sequence. (PDF) [file ppat.1011243.s018.pdf]

A

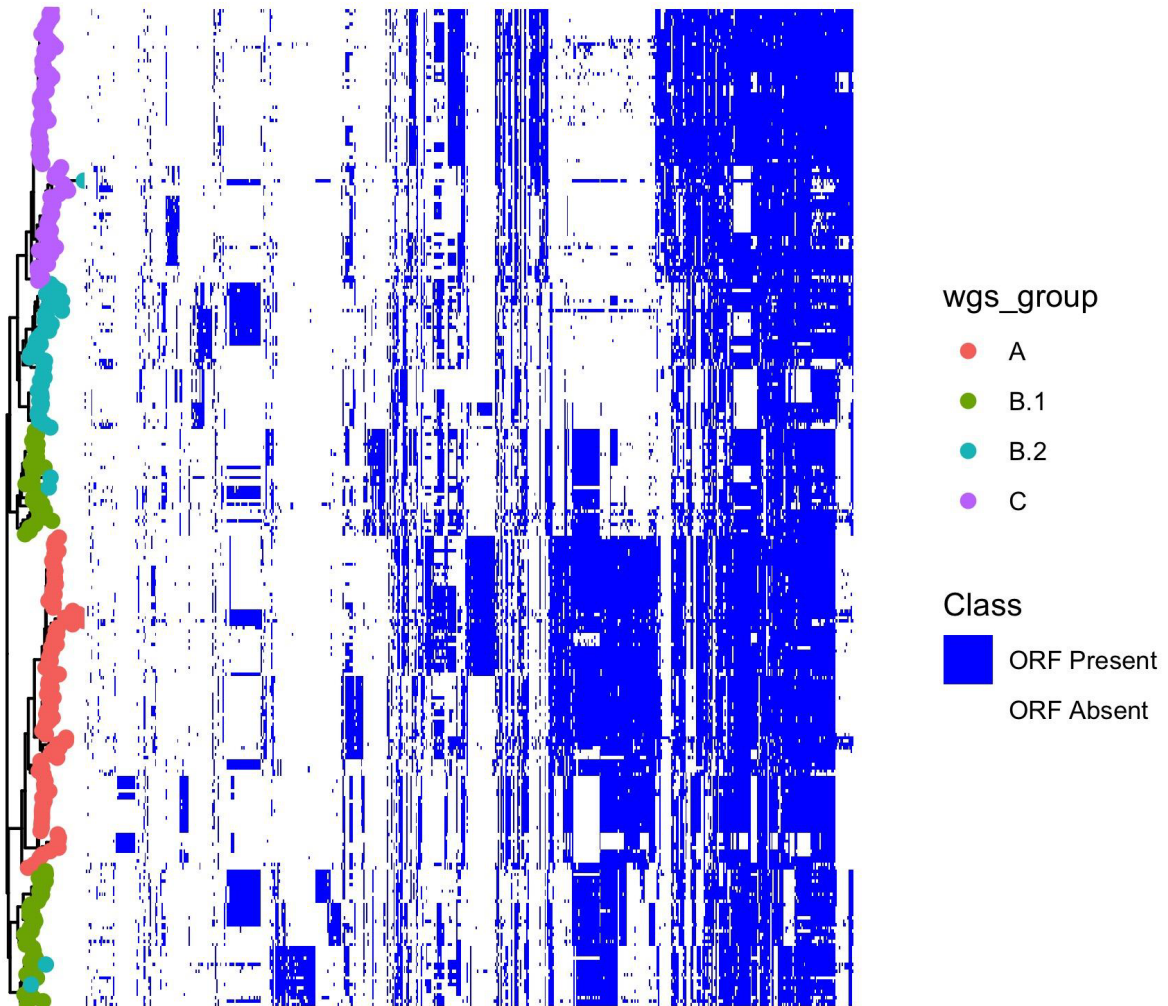

B

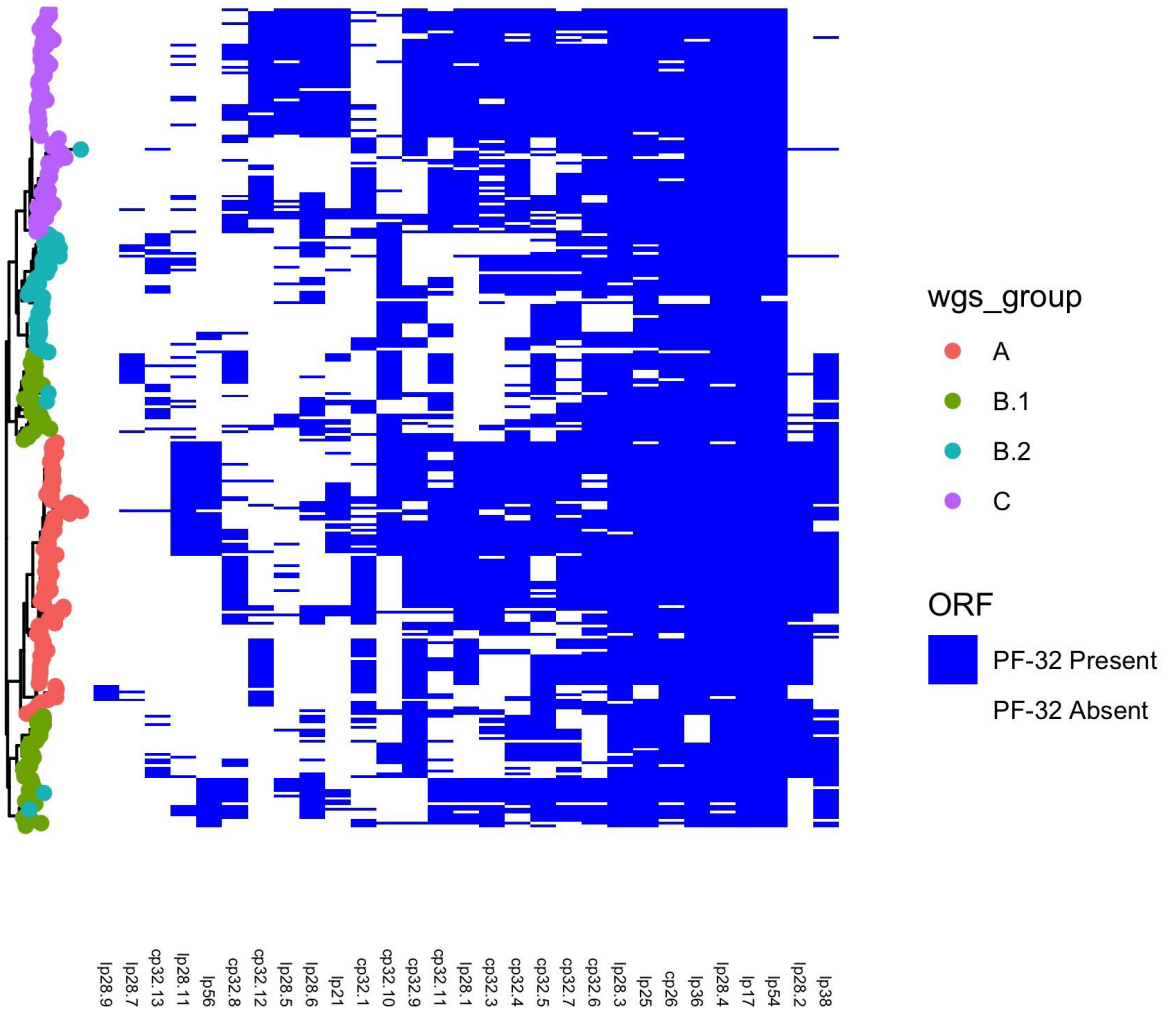

S6 Fig

Supplement: S6 Fig — A. Phylogenetic tree created from the accessory genome using Roary with accessory genome elements plotted according to their presence/absence in individual strains. B. Phylogenetic tree created from the accessory genome with PFam32 plasmid compatibility sequences plotted according to the presence/absence in individual strains. (PDF) [file ppat.1011243.s019.pdf]

A

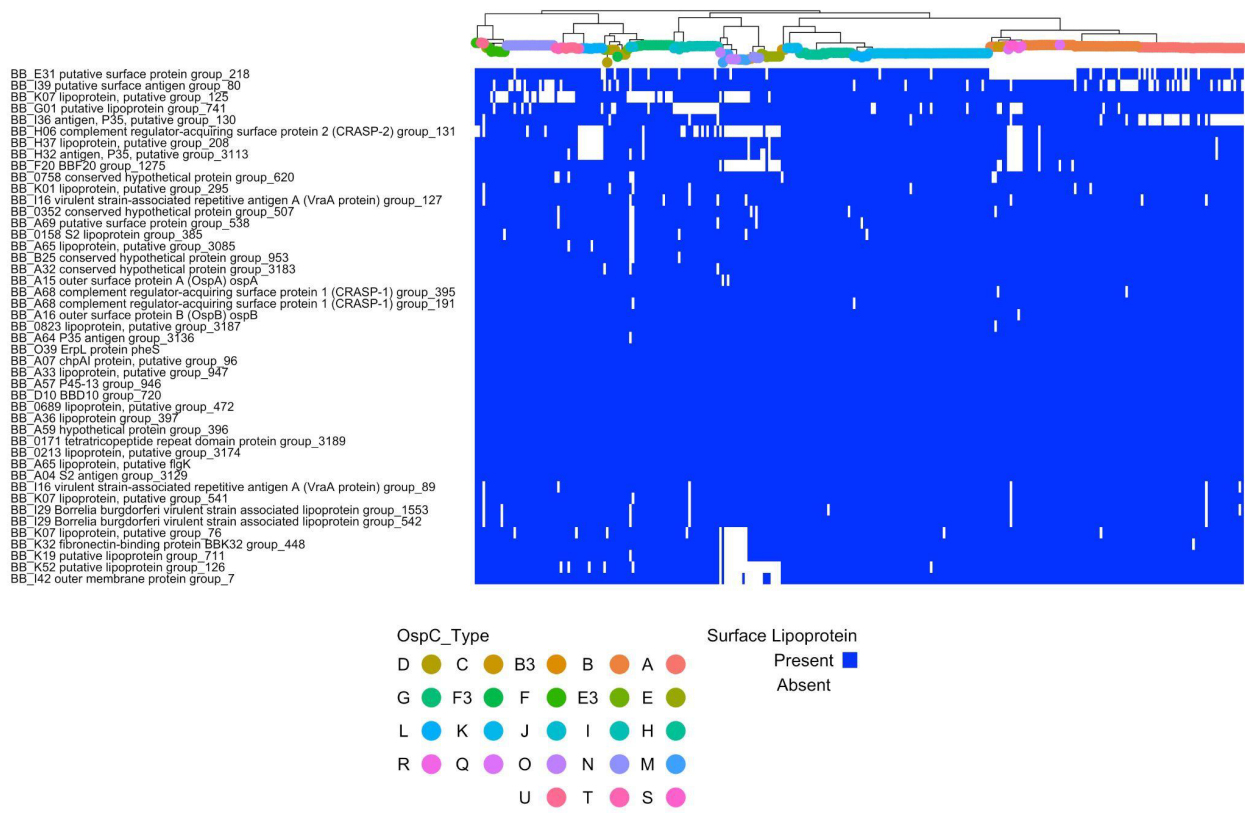

B

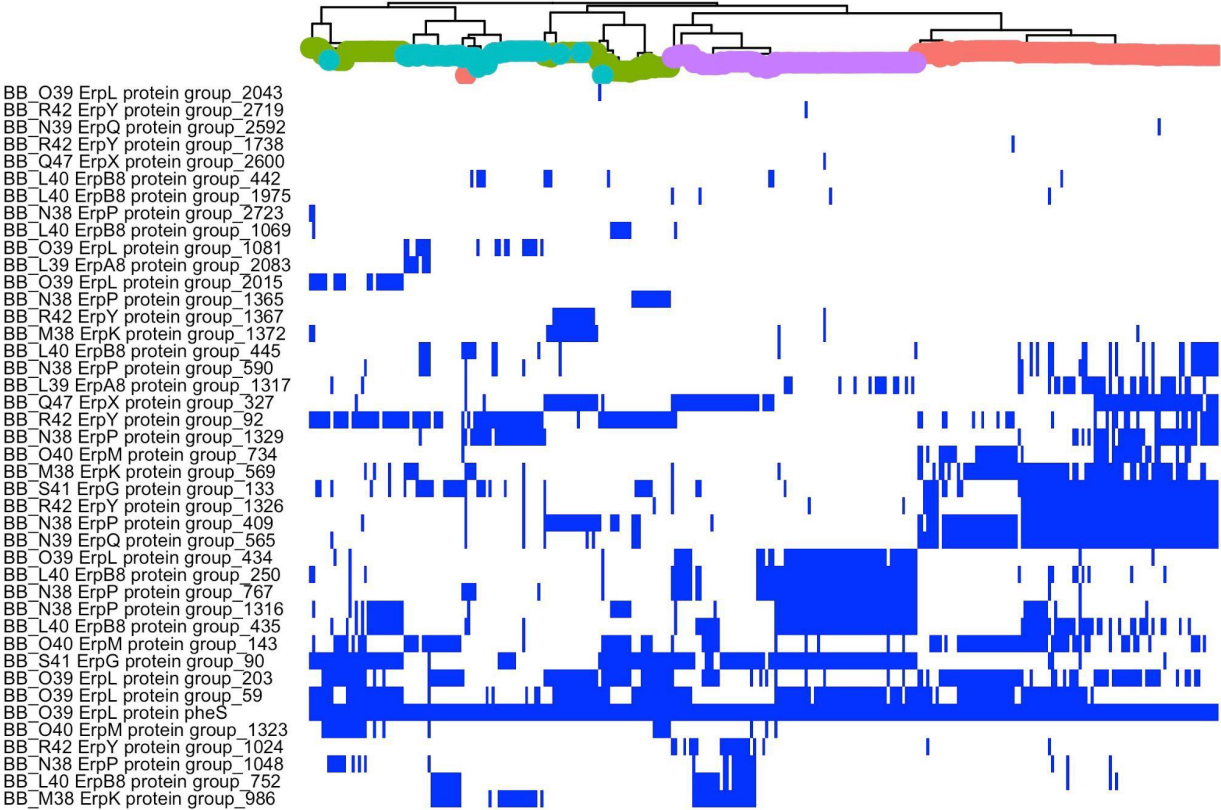

| WGS Group | Surface Lipoprotein |
|-----------|---------------------|
| A         | Present             |
| B.1       | Absent              |
| B.2       |                     |
| C         |                     |

C

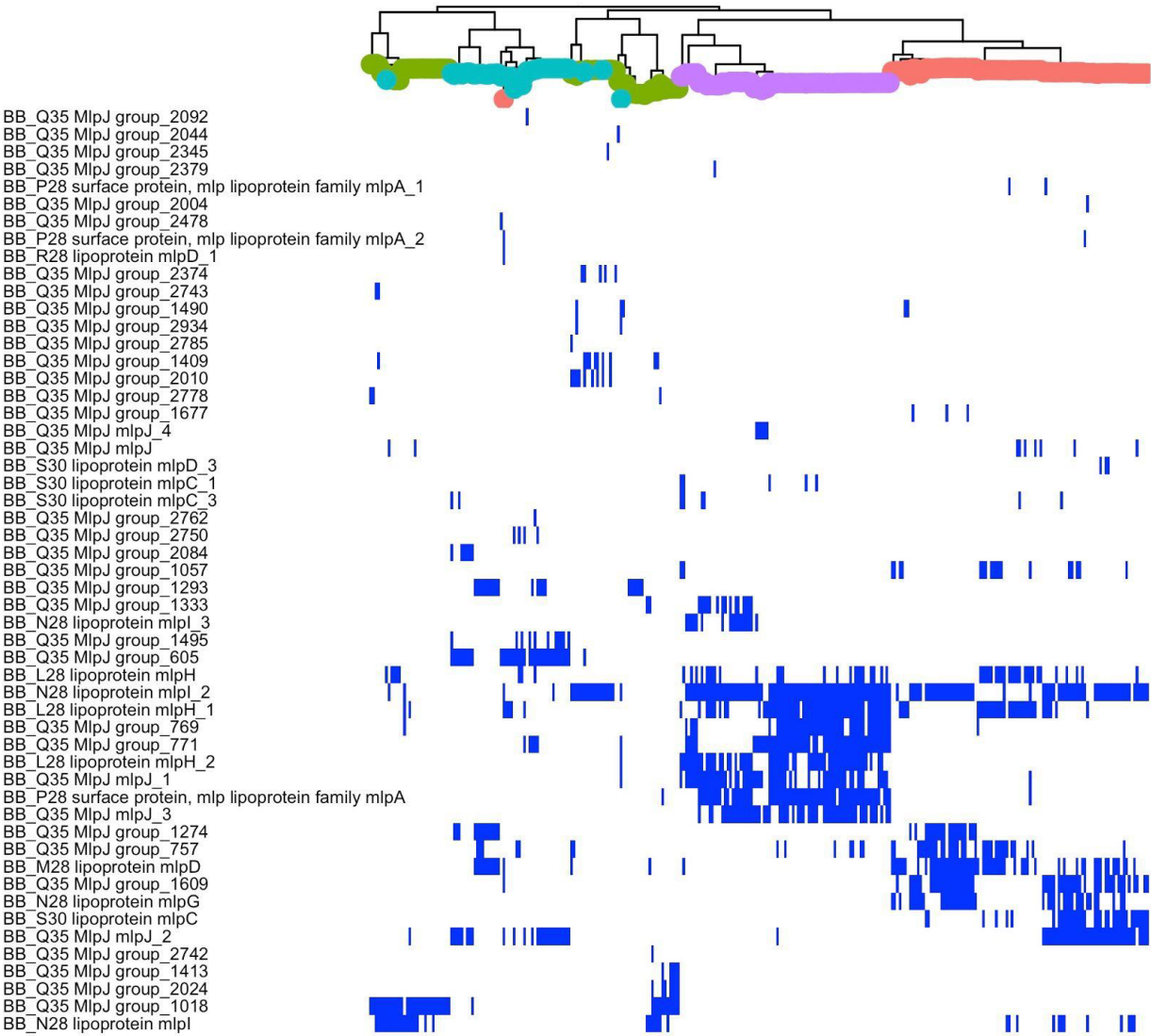

D

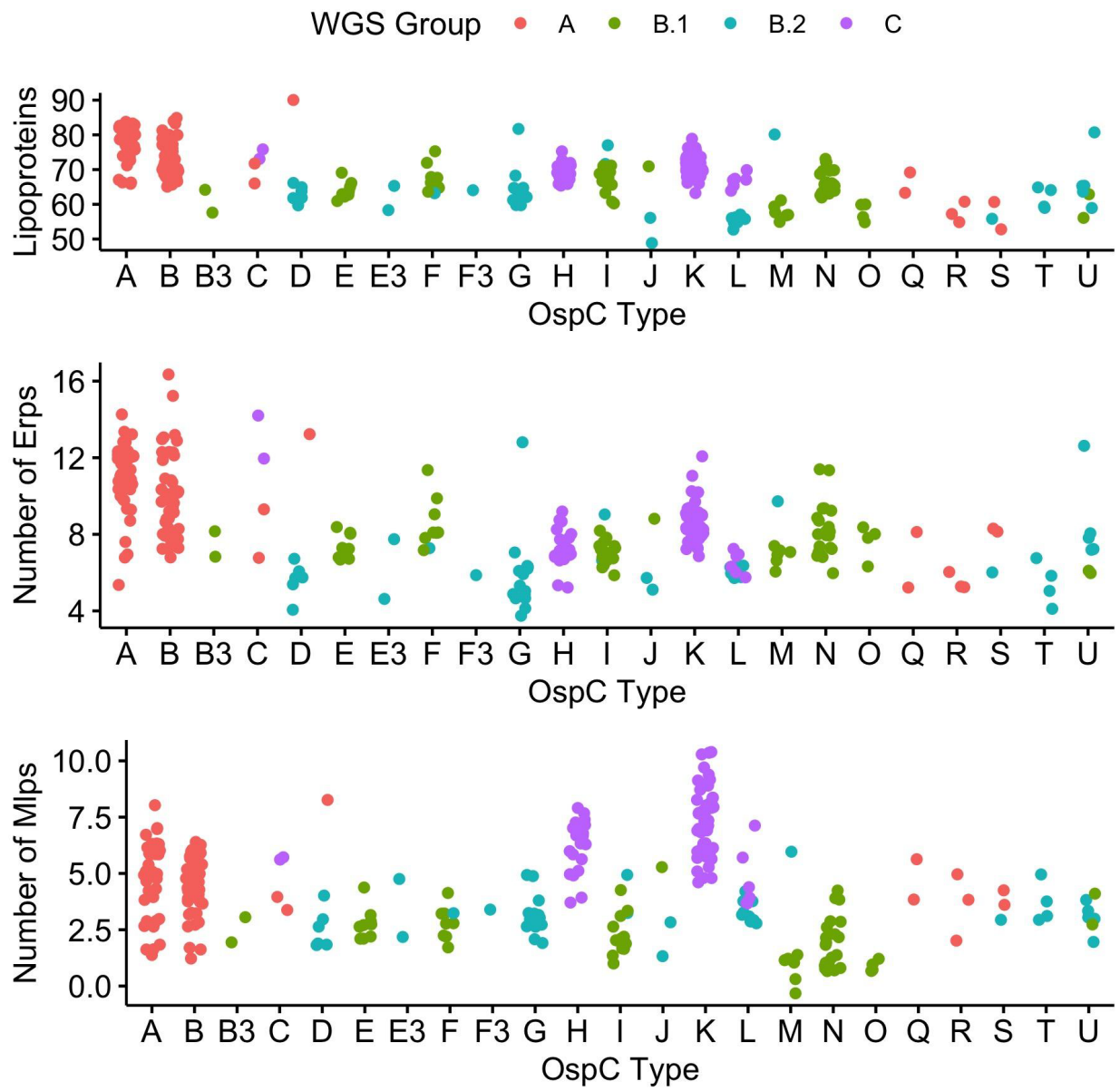

**E**

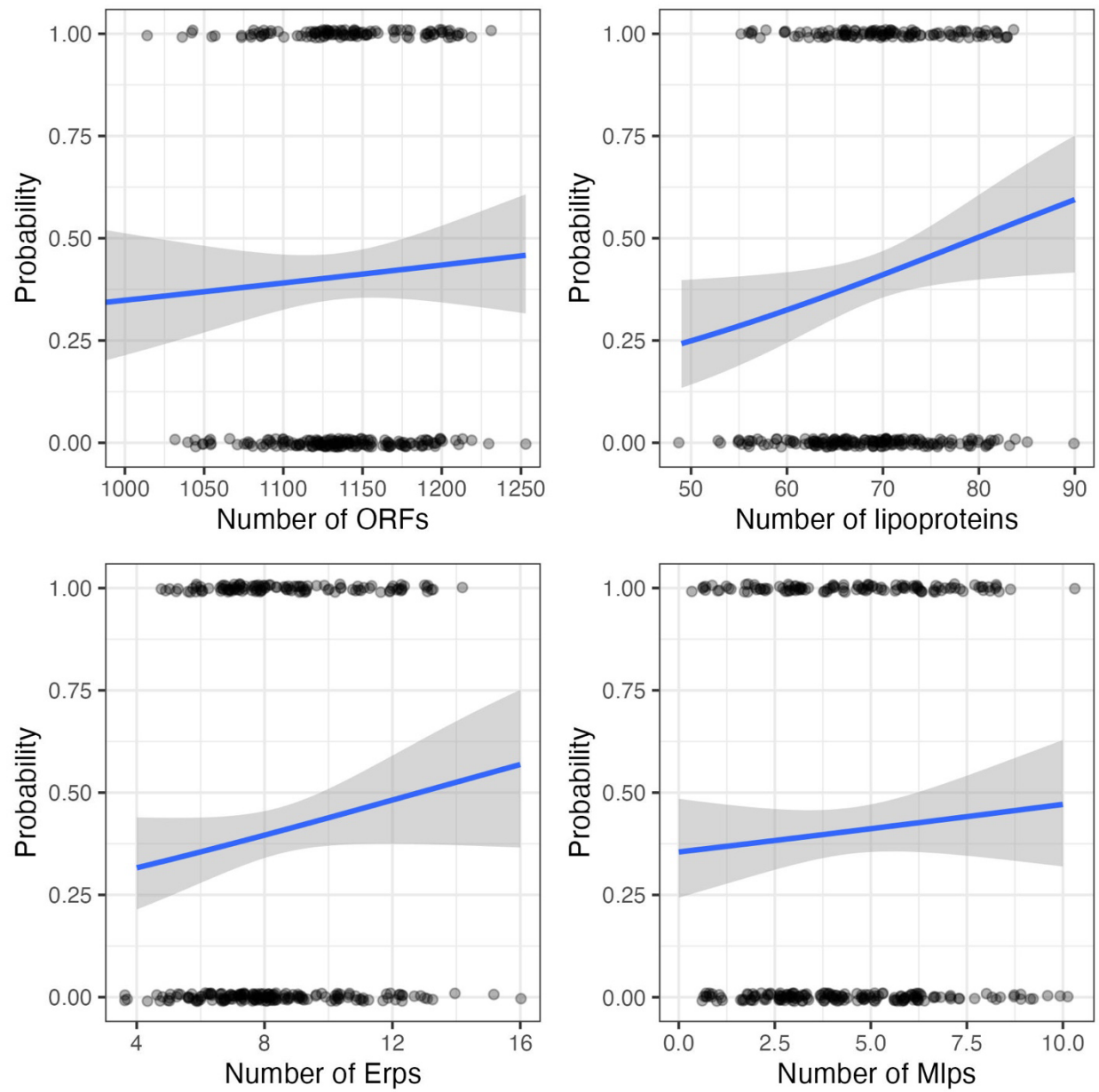

F

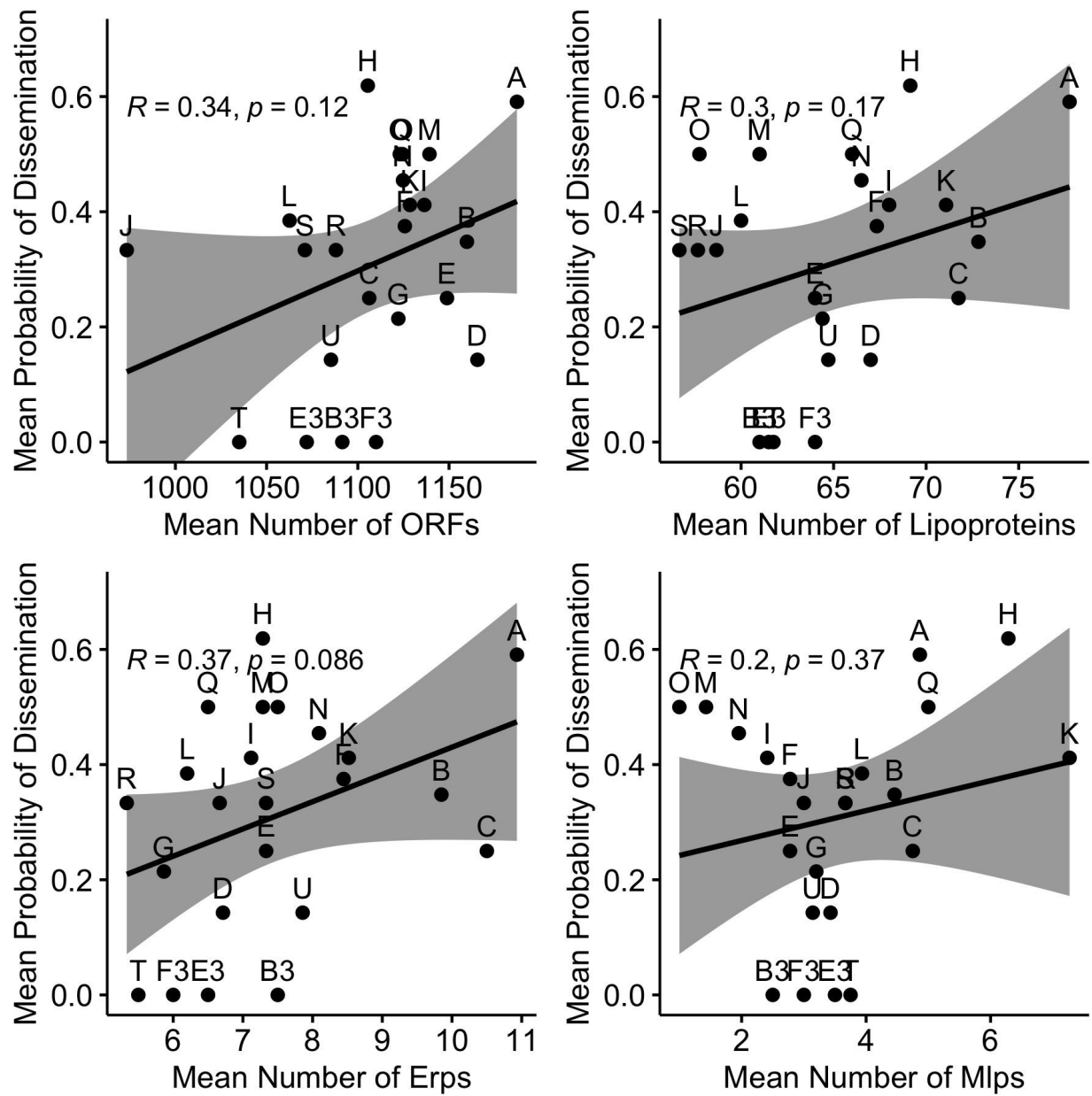

S7 Fig

Supplement: S7 Fig — A. Bb core surface lipoproteome: Core genome phylogeny with tips colored by OspC type (colored according to the scheme in Fig 5) with a matrix of presence (blue) or absence (white) for surface lipoproteins. Surface-exposed lipoproteins present in at least 80% of strains were considered to be part of the core lipoproteome. B and C. Core genome phylogeny with presence/absence of Erp (C) homology groups and Mlp (D) homology group. D. The number of surface-exposed lipoproteins (top panel), Erps (middle panel), and Mlps (bottom panel) by OspC type. E. Logistic regression modeling the probability of dissemination by number of ORF (top left, regression coefficient for slope, β1 = 0.002 +/- 0.002, p = 0.450), number of surface-exposed lipoproteins (top right, β1 = 0.037 +/- 0.017, p = 0.03, logistic regression), number of Erps (bottom left, β1 = 0.087 +/- 0.053, p = 0.10, logistic regression), and number of Mlps (bottom right, β1 = 0.048 +/- 0.055 p = 0.38, logistic regression). The observed data used to build the regression model are plotted. Each isolate is a point whose y-value has been assigned 1 to denote a disseminated phenotype or 0 to denote a non-disseminated phenotype. A small amount of noise has been added to the y-coordinate to display overlapping points. F. For each OspC type, mean probability of dissemination vs mean number of ORF (top left), mean number of surface-exposed lipoproteins (top right), mean number of Erps (bottom left), and mean number of Mlps (bottom right). (PDF) [file ppat.1011243.s020.pdf]

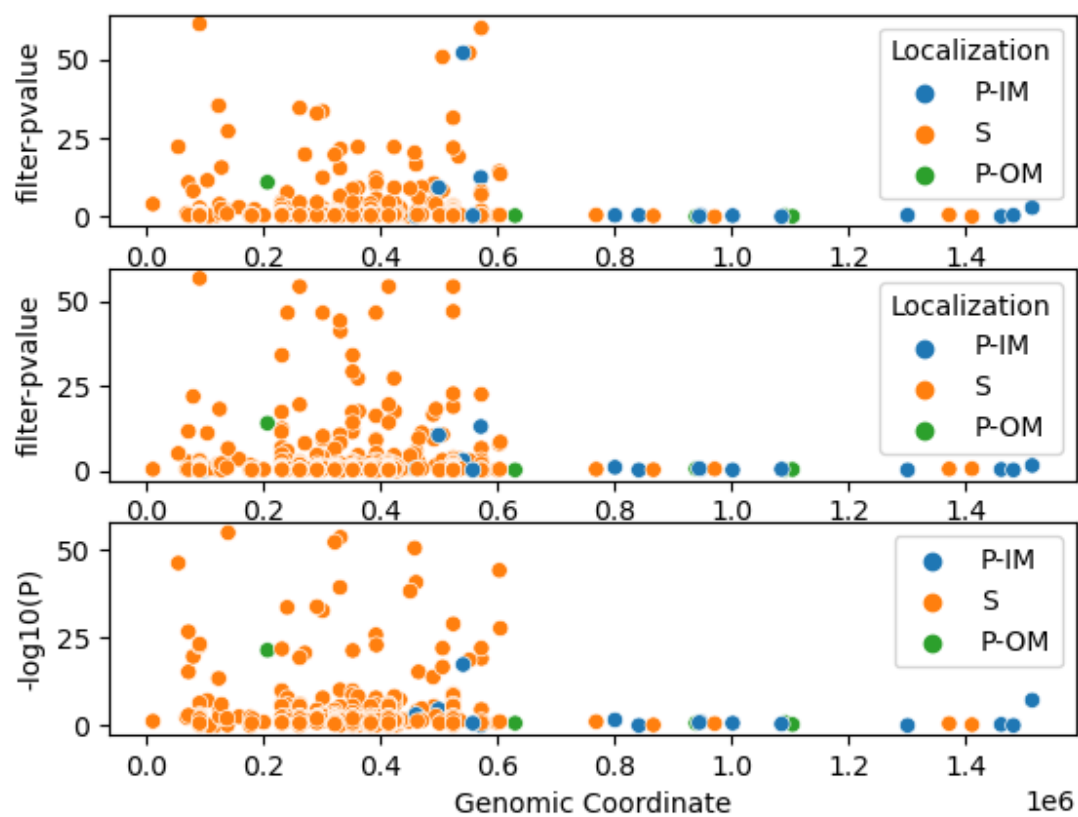

**S8 Fig**

Supplement: S8 Fig — Manhattan Plots showing the association of individual lipoproteins with OspC type A (top panel), Osp C type K (middle panel), and RST1 (bottom panel). Individual lipoproteins are annotated by their localization. The scale is in 1,000,000 base pairs, with the ordering of plasmids and the chromosome as in Fig 7. P-IM: Periplasmic inner membrane. POM: Periplasmic outer membrane. S: surface. (PDF) [file ppat.1011243.s021.pdf]
