# Supplementary material for: Whole genome sequencing of human Borrelia burgdorferi isolates reveals linked blocks of accessory genome elements located on plasmids and associated with human dissemination
Source: PLoS Pathog. 2023 Aug 31;19(8):e1011243. doi: 10.1371/journal.ppat.1011243 (PMC10470944; doi:10.1371/journal.ppat.1011243)
Supplement: Attachment — Submitted filename: Response to review.pdf [file ppat.1011243.s023.pdf]

Part I - Summary
Please use this section to discuss strengths/weaknesses of study, novelty/significance, general execution and scholarship.

Reviewer #1: The manuscript by Lemieux et al entitled “Whole genome sequencing of Borrelia burgdorferi isolates reveals linked clusters of plasmid-borne accessory genome elements associated with virulence” presents a monumental effort to sequence genomes of human derived isolate. This will be a powerful resource for researchers in the field going forward. The primary hypothesis stated in the title and abstract suggests that the authors identified genetic loci associated with dissemination in vertebrate hosts. However, the authors presented a large number of additional analyses that are tangentially related to this hypothesis. There are several major and minor aspects of this work that could use the authors attention in order to make the greatest impact on scientific progress.

Reviewer #2: The manuscript by Lemieux et al performed genome-wide association study of human virulence of Lyme strains using ~300 clinical B. burgdorferi isolates collected from across North America and Europe. While the study conclusions (invasiveness of RST-1/OspC-A strains) are largely confirmatory of earlier studies using much fewer isolates and fewer genetic loci, the study is much larger in the genome scale, in the number of clinical samples, as well as in the geographic coverage. The sampling design is as comprehensive as one could amass (except maybe the exclusion of Bbss from Asia).

The analytical methods are rigorous, going beyond regular phylogenetic reconstruction. I'm most gratified to see the lineage correction necessary to control for the effect of tight genome-wide linkage.

The authors have meticulously laid out limitations of the study, including a lack of full plasmid assembly (due to short reads), sampling biases, and spurious association due to strong linkage.

I have some minor suggestions regarding data interpretation, methodolgy, and data sharing.

Reviewer #3: Lemieux et al. have sequenced and analysed the genomes of 299 B. burgdorferi s.s. human clinical isolates and used genome-wide association analysis methods to provide insights into replicons and ORFs that are correlated with disease severity, namely the frequency of dissemination. Overall this paper has been produced to a high standard and I have only few comments requiring attention by the authors. Pending these minor corrections I strongly recommend this manuscript for publication in PLOS Pathogens.

Part II – Major Issues: Key Experiments Required for Acceptance

Please use this section to detail the key new experiments or modifications of existing experiments that should be absolutely required to validate study conclusions.

Generally, there should be no more than 3 such required experiments or major modifications for a "Major Revision" recommendation. If more than 3 experiments are necessary to validate the study conclusions, then you are encouraged to recommend "Reject".

Reviewer #1: Major Aspects

1. In my opinion, there are too many hypotheses assessed and it is hard to figure how they relate to the “linked clusters of plasmid-borne accessory genome elements associated with virulence.” For example, there is quite a bit of space dedicated to comparing WGS typing with other typing methods. Important work, no doubt, but distracts from the main thesis. In fact, there are 4 pages of results prior to any data addressing the correlation between loci and dissemination. ¬This, along with very dense and often confusing prose and some figures that are difficult to interpret, will diminish the impact of this work.

We thank the reviewer for pointing this out. We have revised the main text in several places to clarify the hypothesis tested. The introduction includes a sentence: “We hypothesized that genetic variation in *Bb* open reading frames (ORFs) and plasmids among strains was associated with differences in dissemination in humans.” The conclusion now includes a sentence, “We hypothesized that specific genetic elements were associated with human dissemination in patients.”

We have also cut and/or reduced sections that were unrelated to this hypothesis. For example, we have supplemental Note 1 that discussed divergence times. We have also removed most of the discussion of phylogeography. We believe these changes have improved the clarity and focus of the manuscript, and we plan to investigate the topics of ancestral divergence times and phylogeography in greater detail in a future analysis.

2. It is not entirely clear if the authors support their primary hypothesis. I apologize in advance if I have misinterpreted the data/explanation. In my reading, the authors show that there is strong genetic linkage tight within regions and pretty strong linkage among regions. The show that genetic linkage blocks associate with dissemination but also that the linkage blocks are basically whole genomes. If this is the case, it is not clear that the WGS typing system offers additional information about human dissemination probability than the other typing systems. Also due to the tight genetic linkage, no loci can be correlated with dissemination as GWAS type studies require some degree of reassortment.

The reviewer highlights important complexities of the analysis and we have taken the opportunity to clarify parts of the paper in response to this comment.

- We have revised Figure 5A to include all ORF groups, not only strain variable ones, to demonstrate that the linkage blocks do not represent the whole genome, but rather a subset of the genome.
- We have added a paragraph to the discussion explicitly on this topic. “Our findings support this hypothesis by identifying groups of genes associated with dissemination in humans, but due to the clonal population of *Bb,* it is not possible to resolve the specific genetic elements within these groups without further investigation. Using unadjusted, univariate associate models, virtually all dissemination-associated genes were found on plasmids. However, after correction for spirochete genetic structure due to lineage, only weak locus-specific associations were observed. Distinguishing causal alleles from non-causal, linked alleles requires statistical reassortment, usually in the form of recombination, and/or experimental data. Because reassortment does occur (the correlation between alleles is not perfect), larger sample sizes can help narrow the list of potential causal loci. Improved statistical models that explicitly incorporate the joint distribution of covariates among isolates would also help.”

3. The idea that there is a dissemination phenotype, as opposed to a HUMAN dissemination phenotype, appears to be assumed. I do not think is assumption is well supported. It seems more likely that all strains disseminate in some vertebrate species in order to increase the efficacy of the tick-host-tick cycle.

We agree and have revised the text to clarify in multiple places that we are investigating human dissemination.

4. There are several issues that could use additional clarity.
a. The majority of strains were cultured from EMs. My understanding is that many EMs contain multiple strains identified by direct PCR and OspC or RST typing but cultures from EMs tend to have fewer types. If this is the case, it is not clear if the strains sequenced here are the strains causing the disseminated infections.

We agree and have added a line to the Discussion section, "FFurther, because we did not genotype blood isolates for this study, we cannot rule out that the strain that disseminated to blood was different than those cultured from the EM skin biopsies. However, based on past experience at NYMC, where skin and blood cultures were frequently obtained from the same patient, the majority (>90%) of *Bb* genotypes recovered from blood matched those in skin (personal communication: I.S. and G.W.)."

b. Aligning short reads to B31 genome may result in biases in gene content analyses. It is likely that more closely related strains will align better to the reference genome and thus will have an artificially inflated number of ORFs. This may be the reason the authors observe that WGS type A strains have generally higher ORF content.

This may not have been clear in our initial manuscript, but the ORF counts were obtained from the *de novo* assemblies. We have added a line to the text which reads, "These differences are not attributable to reference genome bias because the ORFs counts were derived from annotated *de novo* assemblies."

c. Short read sequencing, also used in this work, was noted as an issue with Bbss genomic analyses due to the many plasmids and repeat sequences "The sheer number of plasmids and their extreme homology has made sequencing and assembly of complete Bbss genomes a major challenge, particularly with widely-used short read sequencing methods [13]." It is not clear how the authors overcame this challenge.

We thank the reviewer for pointing out that this was not clear. We have added caveats, such as describing the plasmid association maps as provisional given the limitations of short reads. We have added clarifying text to the conclusion to explain that our methods overcome this limitation in some ways but not in others. "The complex structure of the *Bb* genome further complicates the identification of causal loci because the genes in dissemination-associated clusters are predominantly found on plasmids. Integrating plasmid maps with associations at the level of individual ORFs provides a clearer view of the potential determinants of distinct phenotypes. While we cannot yet resolve the causative loci on lp28-1 or lp56 that enhance the pathogenicity of OspC type A strains, we highlight candidate loci and quantify the statistical evidence for each locus considered. Because of strong linkage among multiple loci, identifying causal loci requires additional information. ORFs on these plasmids such as BB_Q67 (which encodes a restriction enzyme modification system [91,92]), BB_Q09, BB_Q05, BB_Q06, BB_Q07, and other plasmids such as BB_J31, BB_J41 (Supplemental Table 8) are among tightly linked to the OspC type A genotype and are candidates for further experimental examination. However, without complete plasmid sequences, the spatial context of these associations and the physical structure of linkage are not resolved. Long-read sequencing will be necessary to define these relationships and establish a definitive map of plasmids because of the frequent, complex exchanges of genes and gene blocks among plasmids [14,16]."

d. I do not fully understand the hypothesis being tested with the plasmid content analyses. It seems the assumption is that plasmid presence/absence correlates with gene presence/absence and it is these genes that affect the human dissemination phenotype. But it has been shown that plasmids differ among strains but gene content is pretty similar as genes move around on plasmids.

We have added clarification in the introduction and conclusions on the hypothesis tested and what we found. This includes a new paragraph in the conclusion, which states, “We hypothesized that specific genetic elements were associated with dissemination in patients. Our findings support this hypothesis by identifying groups of genes associated with dissemination in humans, but due to the clonal population of *Bb,* it is not possible to resolve the specific genetic elements within these groups without further investigation.”

We have also added additional references, such as to Qiu, …, Casjens BMC Genomics 2018 showing the frequency of exchanges between plasmids that complicate these analyses. As noted above, we describe our plasmid presence/absence maps as provisional, requiring confirmation with long-read sequencing.

e. There are several statistical tests that were not well explained in the methods that I do not understand.

We have added additional detail to the methods, improved the annotation of figures, and clarified figure legends in several places to better describe the statistical methods used. We have also added a table (Supplemental Table 4) that summarizes the contingency tables used for testing the association between genotype and dissemination.

Reviewer #2: 1. Data interpretation & conclusion.

The authors conclude the particular invasiveness of OspC Type A (RST1) and possibly associated genetic elements (the lp28-1 and lp56, dbpA). The authors focused on presence/absence of genomic loci/plasmids. Other types of genomic variations might just be as important, if not more, considering a general lack of presence/absence of lineage-specific genes or plasmids.

For example, contribution of gene copy numbers and in multi-copy paralogous loci (e.g., vlsE and cspA). The authors have identified single-locus ortholgous groups, but haven't performed gain/loss analysis of paralogous copies. This is not a requirement for additional analysis, just a reminder for future work.

Further, the tightly shared pan-genomes among the strains point to the importance of allelic differences at lipoprotein loci (including dbpA. ospC, vlsE, and many other hypervariable host-interacting genes) as contributors to human invasiveness.

We thank the reviewer for pointing out that this was not clearly stated. We have added text to the Discussion which reads: “We do not study all types of genetic variation. In particular, copy number variants (CNVs) and single nucleotide polymorphisms (SNPs) are not studied here. Short read methods are not ideal for the study of CNVs. SNPs are incorporated indirectly through the measure of overall sequence similarity (BLAST identity) used to split ortholog clusters, but a detailed association study of SNPs requires a larger sample size.”

We have also rewritten portions of the conclusion to be more clear on these topics. For example, the Discussion now contains two rewritten paragraphs on these issues, “Both gene dosage and allelic variation among lipoproteins present in the same quantity may be important. For example, at the level of allelic variation, distinct homology groups of OspC and DbpA are associated with the OspC type A genotype in this study. Previous experimental work has shown that specific allelic variants of DpbA promote dissemination and alter tissue tropism in a mouse model of Lyme disease [80]. And allelic variation in OspC alters binding to extracellular matrix components, promotes joint invasion, and modulates joint colonization [81]; OspC has also been shown to bind to plasminogen [82,83], promote resistance in serum killing assays [84], and its role in causing infection can be, under certain circumstances, partially complemented by other surface

lipoproteins [85,86]. Homology groups of DbpA (BB_A24), and specific members of the Erp (BB_M38, BB_L39) and Mlp (BB_Q35) (supplemental data file 2, Figures 6C and 6D) families are associated with dissemination, and the genetic differences among these homology groups represent potential candidates for evaluation in follow-up studies.

At the level of gene dosage, differences were particularly notable among multi-copy gene families such as Erps and Mlp proteins. The statistically-significant relationship between lipoprotein number and probability of dissemination in humans and the borderline-significant relationships for copy number of Erps and Mlps (Figure S7E-F) suggest that varying the amount and diversity of linked clusters of surface lipoproteins—which, individually or in combination, may promote survival in the presence of immune defenses, binding to mammalian host tissues and through other pathogenic mechanisms — may be a general mechanism to facilitate vertebrate infection and, consequently, may underlie strain-specific virulence of *Bb* in humans. Erps are divided into three families that each bind to distinct host components (extracellular matrix, complement component, or complement regulatory protein) [65,87–90]; it is possible that the strain-variable clusters of Erps (Figure S7C, Figure S7E-F) may influence clinical manifestations by modulating strain-specific properties of tissue adhesion or resistance to complement-mediated killing of spirochetes. The functions of Mlp proteins and many other strain-variable lipoproteins remain largely unknown."

We agree with the reviewer on the importance and hope to conduct them in a future study with additional isolates and greater statistical power for these and other types of analysis.

2. Data & code sharing. I found the data and code sharing is not up to the standards for reproducibility. The github link is not yet public. The NCBI project page contains only BioSamples, without SRA, not to say contigs, or genome assemblies. The SRAs, data tables, and codes are necessary for proper study replication and future development.

We apologize for the delays in data and code sharing. The github link is now public. Genbank initially rejected our submission of fasta files for assembled genomes but we are working with them to correct this issue. We have submitted the fastq files to SRA and are available under PRJNA923804.

Reviewer #3: (No Response)

Part III – Minor Issues: Editorial and Data Presentation Modifications
Please use this section for editorial suggestions as well as relatively minor modifications of existing data that would enhance clarity.

Reviewer #1:

Below are my notes written as I was reading the manuscript (>> precedes my notes below quotes from the ms). I apologize if they are curt, or the manuscript later addressed them. Page numbers are from the submitted pdf.

Thank you for this incredible genomic resource.
Dustin

We thank the reviewer for the supportive feedback as well the detailed comments. We have responded to each specific point, as outlined below, and feel that the changes have greatly improved the manuscript.

Page: 5
define the individual plasmids¬

>>the genes move around the plasmids?

Page: 8

For example, using RST and OspC genotyping we previously showed that RST1 OspC type A strains have greater proclivity to disseminate, are more immunogenic, are associated with more symptomatic early infection, and with a greater frequency of post-infectious Lyme arthritis.

>>several areas like this that would benefit from specific citations

We have added citations.

The isolates were collected primarily from patients with EM, the initial skin lesion of the infection, over three decades across Northeastern and Midwestern US and Central Europe. We carried out phylogenetic and phylogeographic analysis, and identified particular Bbss genomic groups, plasmids, and individual open reading frames (ORFs) associated with tissue invasive (disseminated) human disease.

>>used EM isolates to identify dissemination correlated genes? Seems odd. Also set up in intro that only EM strains reason why this type of study not possible

We have clarified this. We added a sentence that reads, “Although most isolates were from skin (the site from which *Bb* is most commonly isolated), we assessed dissemination using established methods [7,34] that incorporate clinical signs of dissemination as well as the presence of *Bb* at extra-cutaneous sites as assessed by a positive blood PCR or a positive blood culture (see Methods).”

Page: 10

A measure of bloodstream dissemination was available for 212/299 (70.9%) of isolates, with blood PCR available for 106/299 (35.4%) and blood culture available for a disjoint set of 106/299 (35.4%) of all isolates

>>evidence that the disseminated strains were the ones that were cultured? Regularly find multiple OspC types in EMs by direct PCR, but cultures often have one or at least fewer OspC types.

had a positive PCR

>>PCR from blood? There is some specificity needed

We have clarified that this PCR is from blood.

Short-read next-generation sequencing (NGS) library construction was performed using the 165 Nextera XT Library Prep Kit (Illumina, San Diego, CA).

>>how tell which plasmids exist? Intro said short reads were a problem with this type of study

We have attempted to clarify this with additional and modified text in the introduction and discussion.

We first aligned the contigs to the B31 reference and quantified a plasmid as present or absent if greater than 50% of the reference genome plasmid was covered by contigs.

>>this does not mean the plasmid is present. See Casjen's "genome in flux" paper. There are others by Qiu too.

We have added a phase to note this, “Because homology alone does not necessarily indicate that a plasmid is present [14]”

profile against the assemblies to identify PFam32 genes.

>>Pfam genes are partition factors I think. So they tell you the number of plasmids, but not the plasmid content. The gene content on plasmids differ among strains as I understand it.

There is some ambiguity here. PFam32 genes are partition factors, but they are also used to name plasmids, and some plasmids contain multiple/duplicated PFam32, only one of which is the “real” PFam32. For this reason, we have used a curated list froM Sherwood Casjens of the PFam32 genes used in the Casjens et al. papers. In response to this and other reviewer feedback, we now refer to the plasmid presence/absence maps as provisional and emphasize that long-read sequencing is needed to establish definitive maps.

if a match with <5% identity was present in the list of annotated PFam32 genes, we marked the isolate as having a copy of the closest-matching PFam32 based on sequence identity.

>>confusing. If identity was less than 5% you said it was present?

Thank you for catching this. This was a typo. We meant to write >95% identity. We have corrected it.

Page: 13

Figure 2).

>>There is a much better way to present Fig 2 A-C and E. One phylogeny with 4 columns next to it with the colored labels. No good reason to show the same phylogeny 4 times and obscure the tips with the dots. You have something like this in fig 5 A and B sort of.
Page: 14

Thanks for these helpful suggestions. We have revised and simplified Figure 2 accordingly.

OspC types were monophyletic on the WGS tree (Figure 2E) and on a tree built from OspC sequences (Figure 2F),

>>what is the support on this tree. I expect it is very low. If it is not supported, I am not sure you can make claims about "closely related" OspC sequences

We have annotated the posterior support at internal nodes in the comparison between the WGS tree and OspC tree. Although the support for some fo the OspC tree nodes is weaker, as the reviewer suggests,

many of the nodes are strongly supported, and there are exchanges between well-supported branches of both trees.

For example, the OspC type L isolates from the Midwestern US and Slovenia are on different branches of the core genome phylogenetic tree (Figure S2H).

>>this is probably good evidence of recombination. Please show that type L is supported as a group, which I expect it is

The phylogenetic support for both Slovenian and US Midwest OspC Type L is now visible on the tree in Figure 2. These are both well-supported, and we agree with the reviewer that this is clear evidence of recombination.

Page: 15

OspC sequence distance does not correlate with genome-wide distance between isolates

>>I am not sure that the data support or refute this conclusion. It is technically correct - "does not correlate" - but the inference is that the data are strong enough to say there is no correlation. I am not sure that is the case.

We agree that the use of the term "correlate" in this case is confusing. We have revised the text to say, "the frequency of recombination at the OspC locus means that there are instances in which the genetic distances between OspC sequences is a poor measure of core genome distance." We have also modified the figures showing OspC trees to include node support. We have also added detail to the methods about how OspC trees were constructed.

Population geographic structure:

>>in general, I feel there is too much in one paper. The title/abstract/intro are about genetic elements associated with human dissemination. I am really not sure how this section fits in.

We considered removing this information from the paper entirely, but feel that because the various typing methods are considered together in some analyses, it would be confusing for the reader not to have it referenced somewhere. We have however simplified the analyses presented in the main text. We plan to analyze these relationships in greater detail in future work focused specifically on the topic of genotyping methods.

We next explored the relationship between genetic markers and geography. WGS group was strongly associated with broad geographic region (US Northeast, US Midwest, EU Slovenia) (Fisher's exact test, p < 1 x 10-6), similar to the findings with previously evaluated genetic markers including RST (Fisher's exact test, p < 1 x 10-6) and OspC type (Fisher's exact test, p < 1 x 10-6) (counts by geographic region are shown in Figures 1A-B).

>> I do not understand how these statistical analyses were set up. It seems they should be done with an F statistic (usually reported as Fst) for categorical data and AMOVA for sequence data. I do not know how one can use a 2x2 contingency table with these data (typical for Fisher's Exact), but I also think contingency tables

(rxc) are not appropriate to test these types of hypotheses. Can you please cite the statistical paper that verifies the assumptions of the test with these types of hypotheses.

We thank the reviewer for pointing out that this was confusing. We have modified the analysis to explicitly provide the contingency table for the counts we were using and to provide test statistics on the table counts (now included as Supplemental Table 4). We are treating genotypes as independent and testing for associations between genotypes and numbers of disseminated isolates. Although a more sophisticated analysis would take into account the dependency between genotypes, this is not easy to do without incorporating a measure of relatedness between genotypes. We also feel that it is not needed for the purposes of this analysis, which is to conduct a simple analysis of independent genotypes for use as a crude comparison to the phylogenetic methods in the rest of the paper.

The number of ORFs in the genome differed significantly by region within a given WGS group (Figure 3A).

>> I wonder if this is an artifact. All reads were aligned to a NE US strain, which could lead to higher numbers of matches. This can be seen a bit in the 3B as WGS group A generally has the most ORFs - could be true, could be bc the reference strain was WGS group A

We annotated de novo assemblies, so we do not believe this is an artifact of reference bias. We have clarified this in the methods.

Page: 16

We attempted to define the timing of these exchanges by inferring 284 a time-stamped phylogeny using BEAST (Supplemental Note 1). Together, these models demonstrate a remote (hundreds of thousands to tens of millions of years) TMRCA for human-infectious strains of Bbss, consistent with previous estimates [52]. Precise timing requires more accurate knowledge of the mutation rate in Bbss.

>>I suggest removing this. It is not central to the story and the data do not really support any particular conclusion.

We have removed this as suggested.

We scored isolates as either disseminated or localized based on certain clinical characteristics of the patients from whom they were obtained, particularly having multiple vs 1 EM skin lesion and having neurologic Lyme disease as well as having positive culture or PCR results for Bbss in blood.

>>but did the strain that is sequenced actually disseminate?

We thank the reviewer for raising this point. While we do not report them here (we plan to report these pairs in the future), there are skin/blood pairs available from the NYMC isolates. Based on our experience in limited number of paired skin and blood samples the majority (>90%) of the time the Bb genotype recovered from blood matches the genotype recovered from skin. We include a line in the discussion reporting this, "based on past experience at NYMC, where skin and blood cultures were frequently obtained from the same patient, the majority (>90%) of *Bb* genotypes recovered from blood matched those in skin (personal communication: I.S. and G.W.)."

Page: 17

Figure 3D

no 3D in the ms

Thank you for catching this. We have corrected this to 3C.

Page: 18

Several plasmids, including cp26, lp54, lp36, lp25, lp28-4, lp28-3 are found in nearly all isolates (Figure 4A-B) and others such as cp32-7, cp32-5, cp32-6, cp32-9, and cp32-3 are found in most strains.

the PFam32 is broad, but I'm am not sure that means the plasmid (meaning the gene content) is the same

We have added the term "provisional" to these assignments in the main text. We have added the following text to the conclusion: "Until complete assemblies are available, we regard plasmid assignment for each strain as provisional because both of the methods we used to infer the presence/absence of plasmids have limitations related to the extensive homology among plasmids and the imperfect linkage between PFam32 sequences and the other genes on the plasmid[16]."

Page: 19

confirming that cp26, lp54, lp17, lp28-3, lp28-4 and lp36 were present in nearly all strains whereas other plasmids were more variable.

>>confirms the genes and the PFam were present, but not necessarily together

We have changed the word "confirming" to "suggesting" and included the additional text above about the limitations of these methods in the conclusion.

suggesting that they contain individual genetic elements that may underlie distinct disease phenotypes.

>>weird statement. It says that the phenotype of dissemination in humans is genetically controlled. I am not sure that was ever in doubt.

We agree that dissemination in humans is likely to be a genetic phenotype. We have added the word spirochetal to emphasize that these data support the presence of bacterial genetic determinants of human dissemination rather than human genetic determinants (although we think both are likely important).

Page: 20

The most invasive genotype (WGS A) was associated with the largest pan-genome, whereas the less invasive groups (WGS Group B and C) were associated with smaller genomes (Figure 3A,B).

>>artifact of using WGS A as a reference genome?

We have clarified in the text that this analysis was done using *de novo* assemblies and thus should not be affected by reference bias.

Figure 6A)

>>not much useful information here

We agree that this panel is less informative than the others in Figure 6. In an effort to focus the story in the main text, we have moved it to supplemental Figure 7A.

Page: 21

Aggregating mean effects by OspC types (Figure S7E) showed similar trends.

>>similar to what?

We have added the clause, “to individual isolates, i.e. OspC types with greater numbers of lipoproteins were more likely to disseminate”

Moreover, this finding suggests that the selective forces acting on lp28-3 may differ in Europe and the US.

>>I do not think the data suggest this

We have removed the sentence, which we agree is speculative and not clearly demonstrated by the data.

Page: 22

Figure 7A).

I do not understand this analysis. Are individual SNPs of homologs assessed? It says orthologs, but everything analyzed is the same species. Also, the x-axis is weird for a Manhattan plot.

Thank you for pointing out that this was confusing. We have edited the figure axis for clarity. We have also added the language (Figure 7): “The Y axis plots the P-value for tests of association between each ortholog group and the phenotype of dissemination are shown. For ORFs that aligned to the B31 reference genome, the x axis denotes the annotated position in the genome.” Figure 8: “The Y axis plots the P-value for tests of association between each ortholog group and the lineage marker are shown. For ORFs that aligned to the B31 reference genome, the x axis denotes the annotated position in the genome.”

We have changed to the term “homology groups” for “orthologs” because these are groups within the same species, and some may contain paralogous sequences.

Page: 24

but the relationships among these markers and specific Bbss genes that cause phenotypic differences had not yet been studied due to limitations of existing typing systems and a lack of human isolates.

>>and also linkage disequilibrium, which is present here and also limits the ability to correlate phenotypes with specific genes

We agree and have added text to the conclusion emphasizing this point, "...due to the clonal population structure of the *B. burgdorferi,* with strong linkage between genetic loci*,* it is not possible to resolve the specific genetic elements without further investigation."

Page: 25

Using two different methods to infer the presence or absence of plasmids, we provide the first plasmid presence / absence maps of a large collection of human clinical isolates.

>>not sure this is true. Casjens 2000 and 2012 suggest considerable rearrangement of genes among plasmids. Strains may have the same genes, but on different plasmids (Casjens et al 2012)

We have added the modifier "provisional" to the plasmid presence / absence maps and cited the Casjens et al. 2012 article.

are tightly linked to the OspC type A genotype and are candidates for further experimental study.

>>maybe. Or maybe it is just the linkage to another gene that is causing the correlation with dissemination.

We agree with the reviewer. To clarity, we have added a sentence, "Because of strong linkage among multiple loci, identifying causal loci requires additional information." We have also reworded the sentence to read "among the genes tightly linked" to indicate that there are others also worthy of investigation.

For example, In OspC type A strains, DbpA is strongly linked to OspC type A.

>>is there another option? Only one of the two genes has any variation

We have clarified in the text that this is one homology group of DbpA which is linked to OspC type A.

Page: 26

matrix components,

>>binds plasminogen as well

Thank you for pointing this out. We have added this to the text with references.

The statistically-significant relationship between lipoprotein number and probability of dissemination and the borderline significant relationships for copy number of Erps and Mlps (Figure S7D-E) suggest that varying the amount and diversity of linked clusters of surface lipoproteins—which, individually or in combination, may promote survival in the presence of immune defenses, binding to mammalian host tissues and other pathogenic mechanisms— may be a general mechanism for strain specific virulence of Bbss.

>>This is a tough hypothesis to follow bc it suggests that strains that have fewer lipoproteins have a lower probability of infecting ANY vertebrate host. It seems that the strains that do not infect humans, which are an evolutionary dead end anyway, probably infect other species. Why do they not need lipoproteins to infect chipmunks?

> We thank the reviewer for pointing out that this is confusing. We have reworded the sentence, which now ends, "...may be a general mechanism to facilitate vertebrate infection and, consequently, may underlie strain-specific virulence of *Bb* in humans."

Genes are inherited in blocks; the inheritance pattern of genes within these blocks is strongly correlated such that only infrequently are genes from within a block found in isolates that are outside the block. This pattern is also seen in plasmids, and plasmids are a natural mechanism for this pattern of inheritance.

>>this is one of the most confusing ways of saying that Bbss lineages are clonal (mostly) that I have read. I think you are trying to say that there is very little HGT among strains.
Page: 27

> We thank the reviewer for pointing out that this was confusing. We removed this text and rewritten the conclusion section based on the reviewers feedback. The conclusion now reads, "Our findings support this hypothesis by identifying groups of genes associated with dissemination in humans, but due to the near-clonal population structure of *Bb,* it is not possible to resolve the specific genetic elements within these groups without further investigation. Using unadjusted, univariate associate models, virtually all dissemination-associated genes were found on plasmids. However, after correction for spirochete genetic structure due to lineage, only weak locus-specific associations were observed. Distinguishing causal alleles from non-causal, linked alleles requires statistical reassortment, usually in the form of recombination, and/or experimental data. Because reassortment does occur (lineages are not perfectly clonal), larger sample sizes can help narrow the list of potential causal loci."

beyond identifying genomic elements or groups of correlated genes associated with a phenotype,

>>does this just mean that all we can really say is that certain lineages have relatively stable genome content over generations. So really we can just say that some lineages are associated with dissemination?

> Thank you for raising that this is not clear. The WGS studies add value over lineage alone because of homplasy, but the near-clonal population structure means that the value is not enough to resolve causal alleles. In addition to the conclusion paragraph on the near-clonal population structure, we have added the following sentences clarifying the value added by WGS, "While single-locus markers capture much of the genetic variation among *Bb* human isolates, the presence of homoplasy among a subset of accessory genome elements (i.e. genes that are present or absent in multiple branches of the phylogeny in Figure 5B) means that single-locus markers are an imperfect proxy for strain-specific genetic differences. Thus, association studies linking genotype to phenotype benefit from WGS typing."

Third, our analysis highlights how evolutionary history, geography, and differences in strain genetic diversity interact in complex ways to contribute to clinical heterogeneity in Lyme disease.

>>I am not seeing this.

Thank you for pointing out that this statement needs revision. We have modified as, “our analysis highlights how strain genetic diversity, which is shaped by geographical isolation and evolutionary history, contributes to clinical heterogeneity in Lyme disease”.

Page: 28

In this regard, WGS serves 571 as a gold standard against which other typing methods can be compared, facilitated here by our sequenced and fully-typed set of isolates

>>I do not see what WGS has added here. Not the researchers’ fault. Just that everything is in genetic linkage.

We have removed this language and have modified the text as described above to more accurately balance the benefits and limitations of WGS.

Our PFam32 analysis is limited by an uncertainty as to which gene sequences are contained on the plasmid associated with the PFam32 sequence.

worth citing Casjens and Qiu

We have added a reference to Casjens, .., Qiu 2018

Page: 33

Figure 2: A-B.

>>some indication of node support is needed on all trees. Branch length indicaters too

We have added node support for MCC trees from BEAST and used these trees in the main text. Node support is not readily available for trees constructed by fasttree, so we used IQtree and the ultrafast bootstrap method it implements to quantify node support for ML trees. We branch length scale bars to all trees.

F. OspC tree with tips colored by OspC type. G. OspC tree with tips colored by WGS group. H. WGS tree (left) and OspC tree with identical tips connected by strain lines, colored by OspC type.

>>I do not understand the point of the OspC trees. Recombination within OspC is crazy. I am not even understand how they were made. All prior OspC trees look like starbursts with no resolution.

We have simplified the presentation of Figure S2 and added additional detail to the methods to describe the construction of OspC trees.

Page: 58

D. Probability of dissemination by number

>>I do not understand these plots. The probability of dissemination is above 1 and below 0 in many cases. If each strain is a point and thus either a disseminated or non-disseminated, then the axis should not be PROBABILITY. I assume this is correct bc you used a logistic regression

We have edited the y-axis as suggested and added additional to figure legend to increase clarity.

Page: 61

(Figure 2E-F). We also ran models with a fixed rate across a variety of reasonable values (1e- 10 to 1e-8) (Figure 2G).

>>I do not understand the reference to these figs. The trees in Fig 2 are ML trees, not BEAST MCCs

We have removed this analysis based on the feedback that it was confusing and not definite based on the wide uncertainty ranges.

Reviewer #2:

Fig 2. Could be better shown (without overlapping colored dots) with circular trees?

We have redrawn Figure 2 as suggested. We thank the reviewer for this suggestion, which has improved the clarity of the figure.

Fig 4D. add data points so plasmids could be labeled properly without overlaps

Thanks for this suggestion. We have added the data points to Fig 4D as well as Figure S5C.

Reviewer #3:

Abstract

- Minor grammatical corrections in the attached PDF

Thank you for the suggestions in the attached PDF. We have incorporated the changes as suggested.

Introduction
- Introduction is well written and provides enough background info to understand the context of the study. Some concepts are over explained somewhat. Minor grammatical corrections and questions in the attached PDF.
Methods
- Minor grammatical correction in attached PDF.
- The criteria used to classify disseminated vs localised infection uses multiple clinical parameters but does not take into account time since infection, or time from infection to clinical presentation. This factor could bias your phenotypic classification as the frequency of dissemination would surely increase with time since infection?

We thank the reviewer for this comment. Based on past experience in comparing American andEuropean strains we did not see a clear association of dissemination with time. For example, the median duration from tick-bite to EM in the US is 4-6 days and in Europe it is 10-14 days, yet the US Bbss strains disseminate more frequently within this shorter timeframe. In addition, it should be noted that the dissemination in this manuscript is defined based on the clinical features or laboratory evidence evaluated at the time that the skin biopsy was taken for Bb culture. It thus reflects the ability of each recovered Bb genotype to disseminate within this time frame. Since patients are then treated with antibiotic therapy, it is not possible to predict if certain genotypes would have disseminated given more time. This is a concept of interest for us and with a larger number of isolates we hope to tease it out in detail in future studies.

- The bioinformatics analysis seems on the face of it robust, and used well established tools that are appropriate to answer your questions. However, many of these tools have important parameters that can heavily influence the output. For example, the choice of kmers and assembly mode for SPAdes, and the choice of model of FastTree. I strongly recommend these paraments be included in a supp file or you may link to a git repo that holds the code (or example code).

The code to generate all figures in the manuscript is now public github.com/jacoblemieux/borreliaseq, including the commands to execute all analyses.

The attached PDF includes the following comment, which we wanted to address further. “If genome assemblies are incomplete, how can you be sure that all PF32 genes have been assembled?

Perhaps a more robust method would be to use a package such as Scoary, to statistically identify gene presence/absence with genotyping and dissemination profiles. As plasmid genes are strongly linked and inherited you could then trace back plasmid presence/absence from this data. This may also identity other plasmids encoded genes associated with genotypes or dissemination profiles.”

The association studies we have conducted are very similar to those implemented in Scoary. We have run Scoary as suggested, confirming this (although we have not included the Scoary analysis in the revised manuscript because the results are similar and we we think the mixed-effect logistic regression models implemented in pyseer are better-suited to this problem). We have not yet put together a model in which the presence or absence of linked genes predicts plasmid presence / absence. We think this is feasible but requires a larger training dataset than is currently available because of the recombination and exchange of gene blocks between plasmids.

Results

- Genome completeness. Throughout the MS you refer to your genome as ‘complete genomes’, ‘nearly-complete genomes’, and ‘genome assemblies’. As you used short read data only, I would recommend referring to your data as ‘genome assemblies’. Whole genomes (at least to me) indicate complete gap free replicons.

We have updated the terminology used to “genome assemblies” as suggested.

- Providing some summary statistics on the completeness of your genomes would be helpful at the start of the results. The info in Supp. Table 3 is excellent, but too detailed to provide a quick take-away for the reader.

We have added additional summary information from the supplemental table to the paragraph on genome quality in the text. We have not included a metric of completeness in the text because we do not think the percent of the assembly that aligns to B31 is a good measure of completeness.

- Time-stamped phylogeny:

o Define TMRCA

In response to reviewer feedback, we have removed the portion of the manuscript that estimates TMRCA (see below), so the term has been removed.

o Given the huge estimated time ranges, is this at all informative for the paper? I think this adds very little to the overall story and could be removed.

We have removed this section.

Discussion

- No comments, overall very well written.

Thank you.
