# Supplementary material for: Whole genome sequencing of human Borrelia burgdorferi isolates reveals linked blocks of accessory genome elements located on plasmids and associated with human dissemination
Source: PLoS Pathog. 2023 Aug 31;19(8):e1011243. doi: 10.1371/journal.ppat.1011243 (PMC10470944; doi:10.1371/journal.ppat.1011243)
Supplement: Peer review history

# Peer review: Decision Letter 0

**DOI:** 10.1371/journal.ppat.1011243.r001

**Authors:** D. Scott Samuels, David Skurnik

9 Apr 2023

Jake,

Thank you very much for submitting your manuscript "Whole genome sequencing of *Borrelia burgdorferi* isolates reveals linked clusters of plasmid-borne accessory genome elements associated with virulence" for consideration at PLOS Pathogens. As with all papers reviewed by the journal, your manuscript was reviewed by members of the editorial board and by several independent reviewers. The reviewers and I are impressed with the immense effort. In light of the reviews (below), we would like to invite the resubmission of a significantly revised version that takes into account the reviewers' comments. Please address all of the Major Issues highlighted by Reviewers 1 and 2, especially refocus your hypotheses (and reconsider your title) as well as consider the caveats about genome-wide association studies in a species with high genetic linkage. In addition, there are extensive comments and suggestions from all three reviewers (including an attached PDF version from Reviewer 3) that you need to incoroporate. Lastly, the term "*B. burgdorferi* sensu stricto” (and abbreviation “*Bbss*") is dated and awkward: the other Lyme disease spirochetes (formerly *B. burgdorferi* sensu lato) are all their own species now, so you can just use "*B. burgdorferi*". Furthermore, whether you agree with renaming the genus (*Borreliella*), the name should at least be mentioned and (perhaps briefly addressed).

We cannot make any decision about publication until we have seen the revised manuscript and your response to the reviewers' comments. Your revised manuscript may also be sent to reviewers for further evaluation.

When you are ready to resubmit, please upload the following:

[1] A letter containing a detailed list of your responses to the review comments and a description of the changes you have made in the manuscript. Please note while forming your response, if your article is accepted, you may have the opportunity to make the peer review history publicly available. The record will include editor decision letters (with reviews) and your responses to reviewer comments. If eligible, we will contact you to opt in or out.

[2] Two versions of the revised manuscript: one with either highlights or tracked changes denoting where the text has been changed; the other a clean version (uploaded as the manuscript file).

Important additional instructions are given below your reviewer comments.

Please prepare and submit your revised manuscript within 60 days. If you anticipate any delay, please let us know the expected resubmission date by replying to this email. Please note that revised manuscripts received after the 60-day due date may require evaluation and peer review similar to newly submitted manuscripts.

Thank you again for your submission. We hope that our editorial process has been constructive so far, and we welcome your feedback at any time. Please don't hesitate to contact us if you have any questions or comments.

Best wishes,

Scott

D. Scott Samuels

Academic Editor

PLOS Pathogens

David Skurnik

Section Editor

PLOS Pathogens

Kasturi Haldar

Editor-in-Chief

PLOS Pathogens

​orcid.org/0000-0001-5065-158X

Michael Malim

Editor-in-Chief

PLOS Pathogens

orcid.org/0000-0002-7699-2064

***********************

The term "B. burgdorferi sensu stricto” (and abbreviation “Bbss") is dated and awkward: the other Lyme disease spirochetes (formerly B. burgdorferi sensu lato) are all their own species now, so you can just use "B. burgdorferi". Furthermore, whether you agree with renaming the genus (Borreliella), the name should at least be mentioned and (perhaps briefly addressed).

Reviewer's Responses to Questions

**Part I - Summary**

Please use this section to discuss strengths/weaknesses of study, novelty/significance, general execution and scholarship.

Reviewer #1: The manuscript by Lemieux et al entitled “Whole genome sequencing of Borrelia burgdorferi isolates reveals linked clusters of plasmid-borne accessory genome elements associated with virulence” presents a monumental effort to sequence genomes of human derived isolate. This will be a powerful resource for researchers in the field going forward. The primary hypothesis stated in the title and abstract suggests that the authors identified genetic loci associated with dissemination in vertebrate hosts. However, the authors presented a large number of additional analyses that are tangentially related to this hypothesis. There are several major and minor aspects of this work that could use the authors attention in order to make the greatest impact on scientific progress.

Reviewer #2: The manuscript by Lemieux et al performed genome-wide association study of human virulence of Lyme strains using ~300 clinical B. burgdorferi isolates collected from across North America and Europe. While the study conclusions (invasiveness of RST-1/OspC-A strains) are largely confirmatory of earlier studies using much fewer isolates and fewer genetic loci, the study is much larger in the genome scale, in the number of clinical samples, as well as in the geographic coverage. The sampling design is as comprehensive as one could amass (except maybe the exclusion of Bbss from Asia).

The analytical methods are rigorous, going beyond regular phylogenetic reconstruction. I'm most gratified to see the lineage correction necessary to control for the effect of tight genome-wide linkage.

The authors have meticulously laid out limitations of the study, including a lack of full plasmid assembly (due to short reads), sampling biases, and spurious association due to strong linkage.

I have some minor suggestions regarding data interpretation, methodolgy, and data sharing.

Reviewer #3: Lemieux et al. have sequenced and analysed the genomes of 299 B. burgdorferi s.s. human clinical isolates and used genome-wide association analysis methods to provide insights into replicons and ORFs that are correlated with disease severity, namely the frequency of dissemination. Overall this paper has been produced to a high standard and I have only few comments requiring attention by the authors. Pending these minor corrections I strongly recommend this manuscript for publication in PLOS Pathogens.

**********

**Part II – Major Issues: Key Experiments Required for Acceptance**

Please use this section to detail the key new experiments or modifications of existing experiments that should be absolutely required to validate study conclusions.

Generally, there should be no more than 3 such required experiments or major modifications for a "Major Revision" recommendation. If more than 3 experiments are necessary to validate the study conclusions, then you are encouraged to recommend "Reject".

Reviewer #1: Major Aspects

1. In my opinion, there are too many hypotheses assessed and it is hard to figure how they relate to the “linked clusters of plasmid-borne accessory genome elements associated with virulence.” For example, there is quite a bit of space dedicated to comparing WGS typing with other typing methods. Important work, no doubt, but distracts from the main thesis. In fact, there are 4 pages of results prior to any data addressing the correlation between loci and dissemination. ¬This, along with very dense and often confusing prose and some figures that are difficult to interpret, will diminish the impact of this work.

2. It is not entirely clear if the authors support their primary hypothesis. I apologize in advance if I have misinterpreted the data/explanation. In my reading, the authors show that there is strong genetic linkage tight within regions and pretty strong linkage among regions. The show that genetic linkage blocks associate with dissemination but also that the linkage blocks are basically whole genomes. If this is the case, it is not clear that the WGS typing system offers additional information about human dissemination probability than the other typing systems. Also due to the tight genetic linkage, no loci can be correlated with dissemination as GWAS type studies require some degree of reassortment.

3. The idea that there is a dissemination phenotype, as opposed to a HUMAN dissemination phenotype, appears to be assumed. I do not think is assumption is well supported. It seems more likely that all strains disseminate in some vertebrate species in order to increase the efficacy of the tick-host-tick cycle.

4. There are several issues that could use additional clarity.

a. The majority of strains were cultured from EMs. My understanding is that many EMs contain multiple strains identified by direct PCR and OspC or RST typing but cultures from EMs tend to have fewer types. If this is the case, it is not clear if the strains sequenced here are the strains causing the disseminated infections.

b. Aligning short reads to B31 genome may result in biases in gene content analyses. It is likely that more closely related strains will align better to the reference genome and thus will have an artificially inflated number of ORFs. This may be the reason the authors observe that WGS type A strains have generally higher ORF content.

c. Short read sequencing, also used in this work, was noted as an issue with Bbss genomic analyses due to the many plasmids and repeat sequences “The sheer number of plasmids and their extreme homology has made sequencing and assembly of complete Bbss genomes a major challenge, particularly with widely-used short read sequencing methods [13].” It is not clear how the authors overcame this challenge.

d. I do not fully understand the hypothesis being tested with the plasmid content analyses. It seems the assumption is that plasmid presence/absence correlates with gene presence/absence and it is these genes that affect the human dissemination phenotype. But it has been shown that plasmids differ among strains but gene content is pretty similar as genes move around on plasmids.

e. There are several statistical tests that were not well explained in the methods that I do not understand.

Reviewer #2: 1. Data interpretation & conclusion. The authors conclude the particular invasiveness of OspC Type A (RST1) and possibly associated genetic elements (the lp28-1 and lp56, dbpA). The authors focused on presence/absence of genomic loci/plasmids. Other types of genomic variations might just be as important, if not more, considering a general lack of presence/absence of lineage-specific genes or plasmids.

For example, contribution of gene copy numbers and in multi-copy paralogous loci (e.g., vlsE and cspA). The authors have identified single-locus ortholgous groups, but haven't performed gain/loss analysis of paralogous copies. This is not a requirement for additional analysis, just a reminder for future work.

Further, the tightly shared pan-genomes among the strains point to the importance of allelic differences at lipoprotein loci (including dbpA. ospC, vlsE, and many other hypervariable host-interacting genes) as contributors to human invasiveness.

2. Data & code sharing. I found the data and code sharing is not up to the standards for reproducibility. The github link is not yet public. The NCBI project page contains only BioSamples, without SRA, not to say contigs, or genome assemblies. The SRAs, data tables, and codes are necessary for proper study replication and future development.

Reviewer #3: (No Response)

**********

**Part III – Minor Issues: Editorial and Data Presentation Modifications**

Please use this section for editorial suggestions as well as relatively minor modifications of existing data that would enhance clarity.

Reviewer #1: Below are my notes written as I was reading the manuscript (>> precedes my notes below quotes from the ms). I apologize if they are curt, or the manuscript later addressed them. Page numbers are from the submitted pdf.

Thank you for this incredible genomic resource.

Dustin

Page: 5

define the individual plasmids¬

>>the genes move around the plasmids?

Page: 8

For example, using RST and OspC genotyping we previously showed that RST1 OspC type A strains have greater proclivity to disseminate, are more immunogenic, are associated with more symptomatic early infection, and with a greater frequency of post-infectious Lyme arthritis.

>>several areas like this that would benefit from specific citations

The isolates were collected primarily from patients with EM, the initial skin lesion of the infection, over three decades across Northeastern and Midwestern US and Central Europe. We carried out phylogenetic and phylogeographic analysis, and identified particular Bbss genomic groups, plasmids, and individual open reading frames (ORFs) associated with tissue invasive (disseminated) human disease.

>>used EM isolates to identify dissemination correlated genes? Seems odd. Also set up in intro that only EM strains reason why this type of study not possible

Page: 10

A measure of bloodstream dissemination was available for 212/299 (70.9%) of isolates, with blood PCR available for 106/299 (35.4%) and blood culture available for a disjoint set of 106/299 (35.4%) of all isolates

>>evidence that the disseminated strains were the ones that were cultured? Regularly find multiple OspC types in EMs by direct PCR, but cultures often have one or at least fewer OspC types.

had a positive PCR

>>PCR from blood? There is some specificity needed

Short-read next-generation sequencing (NGS) library construction was performed using the 165 Nextera XT Library Prep Kit (Illumina, San Diego, CA).

>>how tell which plasmids exist? Intro said short reads were a problem with this type of study

We first aligned the contigs to the B31 reference and quantified a plasmid as present or absent if greater than 50% of the reference genome plasmid was covered by contigs.

>>this does not mean the plasmid is present. See Casjen's "genome in flux" paper. There are others by Qiu too.

profile against the assemblies to identify PFam32 genes.

>>Pfam genes are partition factors I think. So they tell you the number of plasmids, but not the plasmid content. The gene content on plasmids differ among strains as I understand it.

if a match with <5% identity was present in the list of annotated PFam32 genes, we marked the isolate as having a copy of the closest-matching PFam32 based on sequence identity.

>>confusing. If identity was less than 5% you said it was present?

Page: 13

Figure 2).

>>There is a much better way to present Fig 2 A-C and E. One phylogeny with 4 columns next to it with the colored labels. No good reason to show the same phylogeny 4 times and obscure the tips with the dots. You have something like this in fig 5 A and B sort of.

Page: 14

OspC types were monophyletic on the WGS tree (Figure 2E) and on a tree built from OspC sequences (Figure 2F),

>>what is the support on this tree. I expect it is very low. If it is not supported, I am not sure you can make claims about "closely related" OspC sequences

For example, the OspC type L isolates from the Midwestern US and Slovenia are on different branches of the core genome phylogenetic tree (Figure S2H).

>>this is probably good evidence of recombination. Please show that type L is supported as a group, which I expect it is

Page: 15

OspC sequence distance does not correlate with genome-wide distance between isolates

>>I am not sure that the data support or refute this conclusion. It is technically correct - "does not correlate" - but the inference is that the data are strong enough to say there is no correlation. I am not sure that is the case.

Population geographic structure:

>>in general, I feel there is too much in one paper. The title/abstract/intro are about genetic elements associated with human dissemination. I am really not sure how this section fits in.

We next explored the relationship between genetic markers and geography. WGS group was strongly associated with broad geographic region (US Northeast, US Midwest, EU Slovenia) (Fisher’s exact test, p < 1 x 10-6), similar to the findings with previously evaluated genetic markers including RST (Fisher’s exact test, p < 1 x 10-6) and OspC type (Fisher’s exact test, p < 1 x 10-6) (counts by geographic region are shown in Figures 1A-B).

>> I do not understand how these statistical analyses were set up. It seems they should be done with an F statistic (usually reported as Fst) for categorical data and AMOVA for sequence data. I do not know how one can use a 2x2 contingency table with these data (typical for Fisher's Exact), but I also think contingency tables (rxc) are not appropriate to test these types of hypotheses. Can you please cite the statistical paper that verifies the assumptions of the test with these types of hypotheses.

The number of ORFs in the genome differed significantly by region within a given WGS group (Figure 3A).

>> I wonder if this is an artifact. All reads were aligned to a NE US strain, which could lead to higher numbers of matches. This can be seen a bit in the 3B as WGS group A generally has the most ORFs - could be true, could be bc the reference strain was WGS group A

Page: 16

We attempted to define the timing of these exchanges by inferring 284 a time-stamped phylogeny using BEAST (Supplemental Note 1). Together, these models demonstrate a remote (hundreds of thousands to tens of millions of years) TMRCA for human-infectious strains of Bbss, consistent with previous estimates [52]. Precise timing requires more accurate knowledge of the mutation rate in Bbss.

>>I suggest removing this. It is not central to the story and the data do not really support any particular conclusion.

We scored isolates as either disseminated or localized based on certain clinical characteristics of the patients from whom they were obtained, particularly having multiple vs 1 EM skin lesion and having neurologic Lyme disease as well as having positive culture or PCR results for Bbss in blood.

>>but did the strain that is sequenced actually disseminate?

Page: 17

Figure 3D

no 3D in the ms

Page: 18

Several plasmids, including cp26, lp54, lp36, lp25, lp28-4, lp28-3 are found in nearly all isolates (Figure 4A-B) and others such as cp32-7, cp32-5, cp32-6, cp32-9, and cp32-3 are found in most strains.

the PFam32 is broad, but I'm am not sure that means the plasmid (meaning the gene content) is the same

Page: 19

confirming that cp26, lp54, lp17, lp28-3, lp28-4 and lp36 were present in nearly all strains whereas other plasmids were more variable.

>>confirms the genes and the PFam were present, but not necessarily together

suggesting that they contain individual genetic elements that may underlie distinct disease phenotypes.

>>weird statement. It says that the phenotype of dissemination in humans is genetically controlled. I am not sure that was ever in doubt.

Page: 20

The most invasive genotype (WGS A) was associated with the largest pan-genome, whereas the less invasive groups (WGS Group B and C) were associated with smaller genomes (Figure 3A,B).

>>artifact of using WGS A as a reference genome?

Figure 6A)

>>not much useful information here

Page: 21

Aggregating mean effects by OspC types (Figure S7E) showed similar trends.

>>similar to what?

Moreover, this finding suggests that the selective forces acting on lp28-3 may differ in Europe and the US.

>>I do not think the data suggest this

Page: 22

Figure 7A).

I do not understand this analysis. Are individual SNPs of homologs assessed? It says orthologs, but everything analyzed is the same species. Also, the x-axis is weird for a Manhattan plot.

Page: 24

but the relationships among these markers and specific Bbss genes that cause phenotypic differences had not yet been studied due to limitations of existing typing systems and a lack of human isolates.

>>and also linkage disequilibrium, which is present here and also limits the ability to correlate phenotypes with specific genes

Page: 25

Using two different methods to infer the presence or absence of plasmids, we provide the first plasmid presence / absence maps of a large collection of human clinical isolates.

>>not sure this is true. Casjens 2000 and 2012 suggest considerable rearrangement of genes among plasmids. Strains may have the same genes, but on different plasmids (Casjens et al 2012)

are tightly linked to the OspC type A genotype and are candidates for further experimental study.

>>maybe. Or maybe it is just the linkage to another gene that is causing the correlation with dissemination.

For example, In OspC type A strains, DbpA is strongly linked to OspC type A.

>>is there another option? Only one of the two genes has any variation

Page: 26

matrix components,

>>binds plasminogen as well

The statistically-significant relationship between lipoprotein number and probability of dissemination and the borderline significant relationships for copy number of Erps and Mlps (Figure S7D-E) suggest that varying the amount and diversity of linked clusters of surface lipoproteins—which, individually or in combination, may promote survival in the presence of immune defenses, binding to mammalian host tissues and other pathogenic mechanisms— may be a general mechanism for strain specific virulence of Bbss.

>>This is a tough hypothesis to follow bc it suggests that strains that have fewer lipoproteins have a lower probability of infecting ANY vertebrate host. It seems that the strains that do not infect humans, which are an evolutionary dead end anyway, probably infect other species. Why do they not need lipoproteins to infect chipmunks?

Genes are inherited in blocks; the inheritance pattern of genes within these blocks is strongly correlated such that only infrequently are genes from within a block found in isolates that are outside the block. This pattern is also seen in plasmids, and plasmids are a natural mechanism for this pattern of inheritance.

>>this is one of the most confusing ways of saying that Bbss lineages are clonal (mostly) that I have read. I think you are trying to say that there is very little HGT among strains.

Page: 27

beyond identifying genomic elements or groups of correlated genes associated with a phenotype,

>>does this just mean that all we can really say is that certain lineages have relatively stable genome content over generations. So really we can just say that some lineages are associated with dissemination?

Third, our analysis highlights how evolutionary history, geography, and differences in strain genetic diversity interact in complex ways to contribute to clinical heterogeneity in Lyme disease.

>>I am not seeing this.

Page: 28

In this regard, WGS serves 571 as a gold standard against which other typing methods can be compared, facilitated here by our sequenced and fully-typed set of isolates

>>I do not see what WGS has added here. Not the researchers’ fault. Just that everything is in genetic linkage.

Our PFam32 analysis is limited by an uncertainty as to which gene sequences are contained on the plasmid associated with the PFam32 sequence.

worth citing Casjens and Qiu

Page: 33

Figure 2: A-B.

>>some indication of node support is needed on all trees. Branch length indicaters too

F. OspC tree with tips colored by OspC type. G. OspC tree with tips colored by WGS group. H. WGS tree (left) and OspC tree with identical tips connected by strain lines, colored by OspC type.

>>I do not understand the point of the OspC trees. Recombination within OspC is crazy. I am not even understand how they were made. All prior OspC trees look like starbursts with no resolution.

Page: 58

D. Probability of dissemination by number

>>I do not understand these plots. The probability of dissemination is above 1 and below 0 in many cases. If each strain is a point and thus either a disseminated or non-disseminated, then the axis should not be PROBABILITY. I assume this is correct bc you used a logistic regression

Page: 61

(Figure 2E-F). We also ran models with a fixed rate across a variety of reasonable values (1e- 10 to 1e-8) (Figure 2G).

>>I do not understand the reference to these figs. The trees in Fig 2 are ML trees, not BEAST MCCs

Reviewer #2: Fig 2. Could be better shown (without overlapping colored dots) with circular trees?

Fig 4D. add data points so plasmids could be labeled properly without overlaps

Reviewer #3: Abstract

- Minor grammatical corrections in the attached PDF

Introduction

- Introduction is well written and provides enough background info to understand the context of the study. Some concepts are over explained somewhat. Minor grammatical corrections and questions in the attached PDF.

Methods

- Minor grammatical correction in attached PDF.

- The criteria used to classify disseminated vs localised infection uses multiple clinical parameters but does not take into account time since infection, or time from infection to clinical presentation. This factor could bias your phenotypic classification as the frequency of dissemination would surely increase with time since infection?

- The bioinformatics analysis seems on the face of it robust, and used well established tools that are appropriate to answer your questions. However, many of these tools have important parameters that can heavily influence the output. For example, the choice of kmers and assembly mode for SPAdes, and the choice of model of FastTree. I strongly recommend these paraments be included in a supp file or you may link to a git repo that holds the code (or example code).

Results

- Genome completeness. Throughout the MS you refer to your genome as ‘complete genomes’, ‘nearly-complete genomes’, and ‘genome assemblies’. As you used short read data only, I would recommend referring to your data as ‘genome assemblies’. Whole genomes (at least to me) indicate complete gap free replicons.

- Providing some summary statistics on the completeness of your genomes would be helpful at the start of the results. The info in Supp. Table 3 is excellent, but too detailed to provide a quick take-away for the reader.

- Time-stamped phylogeny:

o Define TMRCA

o Given the huge estimated time ranges, is this at all informative for the paper? I think this adds very little to the overall story and could be removed.

Discussion

- No comments, overall very well written.

**********

PLOS authors have the option to publish the peer review history of their article (what does this mean?). If published, this will include your full peer review and any attached files.

If you choose “no”, your identity will remain anonymous but your review may still be made public.

**Do you want your identity to be public for this peer review?** For information about this choice, including consent withdrawal, please see our Privacy Policy.

Reviewer #1: **Yes: **Dustin Brisson

Reviewer #2: **Yes: **Weigang Qiu

Reviewer #3: No

Figure Files:

While revising your submission, please upload your figure files to the Preflight Analysis and Conversion Engine (PACE) digital diagnostic tool, https://pacev2.apexcovantage.com. PACE helps ensure that figures meet PLOS requirements. To use PACE, you must first register as a user. Then, login and navigate to the UPLOAD tab, where you will find detailed instructions on how to use the tool. If you encounter any issues or have any questions when using PACE, please email us at figures@plos.org.

Data Requirements:

Please note that, as a condition of publication, PLOS' data policy requires that you make available all data used to draw the conclusions outlined in your manuscript. Data must be deposited in an appropriate repository, included within the body of the manuscript, or uploaded as supporting information. This includes all numerical values that were used to generate graphs, histograms etc.. For an example see here on PLOS Biology: http://www.plosbiology.org/article/info%3Adoi%2F10.1371%2Fjournal.pbio.1001908#s5.

Reproducibility:

To enhance the reproducibility of your results, we recommend that you deposit your laboratory protocols in protocols.io, where a protocol can be assigned its own identifier (DOI) such that it can be cited independently in the future. Additionally, PLOS ONE offers an option to publish peer-reviewed clinical study protocols. Read more information on sharing protocols at https://plos.org/protocols?utm_medium=editorial-email&utm_source=authorletters&utm_campaign=protocols

Attachment Submitted filename: PPATHOGENS-D-23-00364_reviewer.pdf Click here for additional data file.

# Author comment: Author response to Decision Letter 0

**DOI:** 10.1371/journal.ppat.1011243.r002

5 Jun 2023

Attachment Submitted filename: Response to review.pdf Click here for additional data file.

# Editor report: Decision Letter 1

**DOI:** 10.1371/journal.ppat.1011243.r003

**Authors:** D. Scott Samuels, David Skurnik

13 Jun 2023

Jake,

We are pleased to inform you that your manuscript 'Whole genome sequencing of human *Borrelia burgdorferi *isolates reveals linked blocks of accessory genome elements located on plasmids and associated with human dissemination' has been provisionally accepted for publication in PLOS Pathogens.

Before your manuscript can be formally accepted you will need to complete some formatting changes, which you will receive in a follow up email. A member of our team will be in touch with a set of requests.

Please note that your manuscript will not be scheduled for publication until you have made the required changes, so a swift response is appreciated.

IMPORTANT: The editorial review process is now complete. PLOS will only permit corrections to spelling, formatting or significant scientific errors from this point onwards. Requests for major changes, or any which affect the scientific understanding of your work, will cause delays to the publication date of your manuscript.

Should you, your institution's press office or the journal office choose to press release your paper, you will automatically be opted out of early publication. We ask that you notify us now if you or your institution is planning to press release the article. All press must be co-ordinated with PLOS.

Thank you again for supporting Open Access publishing; we are looking forward to publishing your work in PLOS Pathogens.

Best wishes,

Scott

D. Scott Samuels

Academic Editor

PLOS Pathogens

David Skurnik

Section Editor

PLOS Pathogens

Kasturi Haldar

Editor-in-Chief

PLOS Pathogens

​orcid.org/0000-0001-5065-158X

Michael Malim

Editor-in-Chief

PLOS Pathogens

orcid.org/0000-0002-7699-2064

***********************************************************

Reviewer Comments (if any, and for reference):

# Editor report: Acceptance letter

**DOI:** 10.1371/journal.ppat.1011243.r004

**Authors:** D. Scott Samuels, David Skurnik

7 Aug 2023

Dear Dr. Lemieux,

We are delighted to inform you that your manuscript, "Whole genome sequencing of human <i>Borrelia burgdorferi<i/> isolates reveals linked blocks of accessory genome elements located on plasmids and associated with human dissemination," has been formally accepted for publication in PLOS Pathogens.

We have now passed your article onto the PLOS Production Department who will complete the rest of the pre-publication process. All authors will receive a confirmation email upon publication.

The corresponding author will soon be receiving a typeset proof for review, to ensure errors have not been introduced during production. Please review the PDF proof of your manuscript carefully, as this is the last chance to correct any scientific or type-setting errors. Please note that major changes, or those which affect the scientific understanding of the work, will likely cause delays to the publication date of your manuscript. Note: Proofs for Front Matter articles (Pearls, Reviews, Opinions, etc...) are generated on a different schedule and may not be made available as quickly.

Soon after your final files are uploaded, the early version of your manuscript, if you opted to have an early version of your article, will be published online. The date of the early version will be your article's publication date. The final article will be published to the same URL, and all versions of the paper will be accessible to readers.

Thank you again for supporting open-access publishing; we are looking forward to publishing your work in PLOS Pathogens. 

Best regards,

Kasturi Haldar

Editor-in-Chief

PLOS Pathogens

​orcid.org/0000-0001-5065-158X

Michael Malim

Editor-in-Chief

PLOS Pathogens

orcid.org/0000-0002-7699-2064
